# CSI-Otter: isogeny-based (partially) blind signatures from the class group action with a twist

**DOI:** 10.1007/s10623-024-01441-7

**Published:** 2024-07-17

**Authors:** Shuichi Katsumata, Yi-Fu Lai, Jason T. LeGrow, Ling Qin

**Affiliations:** 1 PQShield, Ltd., 267 Banbury Road, Oxford, OX2 7HQ UK; 2 AIST, 2-3-26, Aomi, Koto-Ku, Tokyo, 135-0064 Japan; 3 Department of Mathematics, University of Auckland, 38 Princes Street, Auckland, 1010 New Zealand; 4 Department of Computer Science, Ruhr-University Bochum, Universitätsstraße 150, 44801 Bochum, Germany; 5 Department of Mathematics, Virginia Polytechnic Institute and State University, 225 Stanger Street, Blacksburg, VA 24061 USA

**Keywords:** Isogeny-based cryptography, Blind signatures, Group action-based cryptography, Post-quantum cryptography, 11T71, 13P25, 14K02, 94A60, 94A62

## Abstract

In this paper, we construct the first provably-secure isogeny-based (partially) blind signature scheme. While at a high level the scheme resembles the Schnorr blind signature, our work does not directly follow from that construction, since isogenies do not offer as rich an algebraic structure. Specifically, our protocol does not fit into the *linear identification protocol* abstraction introduced by Hauck, Kiltz, and Loss (EUROCYRPT’19), which was used to generically construct Schnorr-like blind signatures based on modules such as classical groups and lattices. Consequently, our scheme is provably secure in the random oracle model (ROM) against poly-logarithmically-many concurrent sessions assuming the subexponential hardness of the group action inverse problem. In more detail, our blind signature exploits the *quadratic twist* of an elliptic curve in an essential way to endow isogenies with a strictly richer structure than abstract group actions (but still more restrictive than modules). The basic scheme has public key size 128 B and signature size 8 KB under the CSIDH-512 parameter sets—these are the smallest among all provably secure post-quantum secure blind signatures. Relying on a new *ring* variant of the group action inverse problem ($\textsf{rGAIP}$), we can halve the signature size to 4 KB while increasing the public key size to 512 B. We provide preliminary cryptanalysis of ${\textsf{rGAIP}}$ and show that for certain parameter settings, it is essentially as secure as the standard $\textsf{GAIP}$. Finally, we show a novel way to turn our blind signature into a partially blind signature, where we deviate from prior methods since they require hashing into the set of public keys while hiding the corresponding secret key—constructing such a hash function in the isogeny setting remains an open problem.

## About

An extended abstract of this work was published in CRYPTO 2023 [56]. This is a full version of that paper. In particular, in this work we present additional explanation of the framework of Kastner et al. [55] in the context of our work; provide complete security proofs for the blind signature scheme of Sect. 5; present proofs of correctness and blindness for the partially blind signature scheme of Sect. 6; provide proofs of correctness and the honest verifier zero-knowledge property of the sigma protocol of Sect. 7, and; provide a proof of blindness of the blind and partially blind signatures of Sect. 7 with special attention to the setting of adversarially-generated public keys, which may be malformed. In Sect. 5.1 we also provide additional discussion of the sigma protocol which underlies the blind and partially blind signatures of Sects. 5 and 6, while in Sects. 8.2 and 8.3 we provide additional analysis the problem of sampling appropriate primitive roots of unity in $\mathbb {Z}_N^\times$, which is required for our optimizations in Sect. 7.

## Introduction

Blind signatures, introduced by Chaum [27], allow a user to obtain a signature on a message from a signer, while the signer is *blind* to the message it signed. One can think of the physical analogy where a user puts a letter—acting as the message—to be signed into a special carbon paper envelope. The signer can sign the envelope without opening it; his signature is transferred to the letter by the carbon paper, and the letter is never visible to the signer. In practice, it is sometimes necessary to consider the extension of *partially* blind signatures, introduced by Abe and Fujisaki [3], that further allow embedding a message agreed on by both the signer and the user into the signature. The messages can now be divided into public and private parts, where the public part can include, for instance, the expiration date of the signature. While (partially) blind signatures^1^ were originally used to construct e-cash [27, 30, 66], anonymous credentials [20, 22], and e-voting [28, 44], the notion has recently seen renewed interest due to applications in blockchains [21, 85] and privacy-preserving authentication tokens [51, 84].

Currently, the most promising class of efficient blind signatures known to withstand quantum attacks is those based on lattices. We have recently encountered significant progress in lattice-based blind signatures, such as [5, 37, 50, 64], where the signature size currently sits around 50 KB to 10 MB. However, this is still an order of magnitude larger than their classical counterparts, with a signature size ranging from a few hundred bytes to 1 KB. As we see a continuous surge of interest in post-quantum security and better user privacy, we aim to investigate a post-quantum blind signature with a smaller signature size.

One potentially promising path to a post-quantum blind signature with a short signature is to rely on *isogeny*-based constructions. This is because while their signing and verification times are less efficient, standard isogeny-based signature schemes [12, 35, 36] are known to produce comparable or even smaller signatures compared to lattices. In fact, for a more advanced form of signature schemes such as ring signatures and group signatures, isogenies can produce much shorter signatures compared to their lattice counterparts [13, 14].

Unfortunately, at first glance this path seems difficult to follow. Very roughly, there are two approaches to constructing a blind signature. The first approach is based on the Schnorr blind signature [29]. This approach builds on a sigma (or an identification) protocol with a “nice” algebraic property and boosts it into a blind signature by appropriately randomizing the interaction. This nice algebraic property has recently been stated informally to be *modules* [49, 50], where isogenies are not known to be endowed with: isogenies are only *group actions* that are strictly less structured than modules (see Sect. 2.2 for more details). The second approach is based on the generic construction proposed by Fischlin [41] that requires proving, at the minimum, possession of a valid signature of a standard signature scheme using a non-interactive zero-knowledge proof (NIZK). While del Pino and Katsumata [37] and Agrawal et al. [5] recently used this approach to construct more efficient lattice-based blind signatures than were previously known, this seems impractical to translate to the isogeny setting due to the lack of efficient NIZKs for such complex languages.

In summary, while isogenies have the potential to produce the shortest post-quantum blind signatures, it is unclear how we can leverage known approaches to build them. This brings us to the main question of this work:

Can we construct an efficient post-quantum (partially) blind signature scheme from isogenies?

### Our contribution

In this work, we answer the above question in the affirmative through four contributions. Our first contribution is to construct the first post-quantum blind signature based on isogenies (or CSIDH group actions to be more specific) called $\mathsf {CSI\text {-}Otter}$, short for CSI-fish with Or-proof Twisted ThreE-Round protocol. The construction is akin to the Schnorr blind signature [29] but follows a slightly different approach. Unlike previous constructions that required the underlying mathematical tool to be a module [49, 50], we bypass this requirement. The crux of our construction is to effectively use the *quadratic twist* of an elliptic curve, or in layman’s terms, we use the fact that isogenies are *slightly* more expressive than a group action. We build a basic blind signature with public key size 128 B and signature size 8 KB based on the standard group action inverse problem ($\textsf{GAIP}$) over the CSIDH-512 parameter sets. We formally prove that our basic blind signature is secure in the (classical) random oracle model with poly-logarithmically many concurrent signing sessions following the recent work by Kastner et al. [55], assuming the subexponential hardness of the group action inverse problem (or a constant number of concurrent sessions assuming only polynomial hardness). That is, the security proof permits a poly-logarithmic number of signatures to be issued per public key in a concurrent manner. We note that extension to polynomially-many concurrent sessions is impossible, as demonstrated in work of Katasumata et al. [57].

Our second contribution is to provide an optimization of our basic blind signature using a new hardness assumption called the $\zeta _d$-*ring* group action inverse problem ($\zeta _d$-$\textsf{rGAIP}$), where $\zeta _d$ denotes a *d-th primitive root of unity* over $\mathbb {Z}_N$. Informally, $\zeta _d$-$\textsf{rGAIP}$ asserts that given $( [\mathfrak {g}^{s \cdot \zeta ^j_d }] *E_0 )_{j \in [d]}$ for a random exponent $s \overset{_{ \$}}{\leftarrow } \mathbb {Z}_N$ and base elliptic curve $E_0:y^2 = x^3 + x$, it is difficult to solve for *s*. Note that when $d = 2$, we have $\zeta _2 = -1$ and we recover the standard $\textsf{GAIP}$, where $[\mathfrak {g}^{-s}] *E_0$ is the (efficiently computable) quadratic twist of $[\mathfrak {g}^{s}] *E_0$. At a high level, $\zeta _d$-$\textsf{rGAIP}$ allows us to use a larger challenge space for the underlying sigma protocol by increasing the public key. This in turn implies that the number of parallel repetitions can be lowered compared to our basic blind signature, and effectively, we obtain a public key size of $(128 \cdot d)$ B and signature size of roughly $(8/\log _2 d)$ KB based on $\zeta _d$-$\textsf{rGAIP}$. Our construction is generic and works for any group actions for which the $\zeta _d$-$\textsf{rGAIP}$ is hard, however, we must show that such group actions exist for it to be useful.

Our third contribution complements our second contribution: we provide a preliminary cryptanalysis on the hardness of $\zeta _d$-$\textsf{rGAIP}$ for the CSIDH-512 parameter sets. We first show that the set of values $\{ \textsf{gcd}(\zeta _d^i-1,N) \}_{i \in [d]}$ relates to the hardness of $\zeta _d$-$\textsf{rGAIP}$. Informally, we create new $\textsf{GAIP}$ instances over a series of subgroups of the class group, where the size of these subgroups relate to each $\textsf{gcd}(\zeta _d^i-1,N)$. Using known attacks against $\textsf{GAIP}$ in a Pohlig-Hellman manner, we can break this newly generated $\textsf{GAIP}$ instances that has a smaller order compared to the $\textsf{GAIP}$ with CSIDH-512. For instance with CSIDH-512, when $d = 7$ or 8, this attack shows that $\zeta _d$-$\textsf{rGAIP}$ only has half the security of ${\textsf{GAIP}}$ over CSIDH-512. On the other hand, for other values of *d* such as $d = 2, 3, 4, 5, 9, \dots$, this attack is no more effective than trying to break $\textsf{GAIP}$ over CSIDH-512. In fact, when $\textsf{gcd}(\zeta _d^i-1,N) = N/\textsf{poly}(n)$ for *n* the security parameter, we show a reduction from the $\zeta _d$-$\textsf{rGAIP}$ to $\textsf{GAIP}$, thus establishing the optimality of our attack for certain parameters such as $d = 3, 5, 9, \ldots$. In the end, due to other correctness constraints, we are only able to instantiate the above optimized blind signature with $d = 4$, which leads to a public key of size 512 B and signature size of 4 KB. While our preliminary cryptanalysis shows that $\zeta _4$-$\textsf{rGAIP}$ is presumably as hard as ${\textsf{GAIP}}$ over CSIDH-512, we leave further cryptanalysis for future work as it is not covered by our reduction to $\textsf{GAIP}$.

Our final contribution is extending our basic blind signature into a *partially* blind signature. While it is straightforward to construct a partially blind signature from a Schnorr-style blind signature in the classical group or the lattice settings, this approach fails in the isogeny setting.^2^ For example, Abe and Okamoto [4] constructed the first partially blind signature, where the main idea was to hash the public message (also known as a *tag*) $\textsf{info}$ to a group element $h_\textsf{info}\in \mathbb {G}$ and let the signer prove that it knows either the exponent of its public key $h = g^a$ or the hashed tag $h_\textsf{info}$. In particular, the underlying sigma protocol proves a 1-out-of-2 (or an OR) relation. In the security proof, the reduction samples $a_\textsf{info}\overset{_{ \$}}{\leftarrow } \mathbb {Z}_p$, programs the random oracle so that $h_\textsf{info}= g^{a_\textsf{info}}$, and uses $a_\textsf{info}$ to simulate the signing algorithm. Unfortunately, this approach is inapplicable in the isogeny setting since we do not know how to map into the set of elliptic curves while simultaneously hiding the exponent. Note that if the exponent is known, any real-world adversary can use the reduction algorithm to forge a signature, thus rendering the scheme insecure.

To this end, we provide a new general approach to constructing partially blind signatures that may be of an independent interest. At the core of our approach is devising a sigma protocol for a *2-out-of-3* relation and embedding the tag $\textsf{info}$ into the signature differently. Since the sigma protocol must also be compatible with the blind signature, we are not able to rely on any 2-out-of-3 sigma protocols for threshold relations such as Cramer-Damg$\mathring{\text {a}}$rd-Schnoemakers’ sigma protocol [33] using Shamir’s secret-sharing scheme [81]. One downside of our partially blind signature is that compared to our blind signature, it requires a signature size roughly three times as large. However, we note that even then, we still achieve a smaller signature size than the lattice-based counterparts.

### Technical overview

We now explain our contributions in detail. We first review the Schnorr blind signature and see where it fails when translating the construction to the isogeny setting. We then explain our basic blind signature $\mathsf {CSI\text {-}Otter}$ that uses the quadratic twist and further show how to extend it to the partially blind setting. Finally, we explain the optimization using the newly introduced $\textsf{rGAIP}$ assumption.

#### Reviewing the Schnorr blind signature

We first recall the Schnorr sigma/identification protocol between a prover with $(\textsf{pk}, \textsf{sk}) = (h = g^a, a) \in \mathbb {G}\times \mathbb {Z}_p$ and a verifier with $\textsf{pk}$. The prover samples $y \overset{_{ \$}}{\leftarrow } \mathbb {Z}_p$ and sends $Y = g^y$ to the verifier. The verifier sends a random challenge $c \overset{_{ \$}}{\leftarrow } \mathbb {Z}_p$ to the prover, where the prover replies with $r = y - a\cdot c$. The verifier is convinced that it was communicating with a prover in possession of $\textsf{sk} = a$ if $g^r \cdot h^c = Y$. Here, if the verifier sets the challenge as $c = \textsf{H}(Y \Vert \textsf{M})$ for a message $\textsf{M}$ and a hash function $\textsf{H}$ modeled as a random oracle, then $\sigma = (c, r)$ serves as a signature based on the Fiat–Shamir transform [40], where the prover is the *signer* and the verifier is the *user with* $\textsf{M}$.

Clearly, this interactive signing protocol does not satisfy *blindness*, which roughly stipulates that a signature cannot be traced back to a specific signing session. In particular, when the user outputs the pair $(\textsf{M}, \sigma )$, the signer will know in which session it signed $\sigma$—or equivalently, the signature $\sigma$ can be traced back to the user—by simply checking when the hash value *c* included in $\sigma$ was used.

The main idea of the Schnorr blind signature [29] is to let the user randomize the interaction so the session transcript becomes independent of the final signature. More explicitly, the user randomizes the interaction so that the final signature becomes $\sigma ' = (c + d, r + z)$, where (*d*, *z*) is uniform over $\mathbb {Z}_p^2$ from the view of the signer. The Schnorr blind signature accomplishes this as follows: When the user receives *Y* as the first-sender message, it samples $(d, z) \overset{_{ \$}}{\leftarrow } \mathbb {Z}_p^2$ and sets $Y' \mathrel {\mathop :}=g^z \cdot Y \cdot h^d$. It then computes $c' = \textsf{H}(Y' \Vert \textsf{M})$ and sends $c \mathrel {\mathop :}=c' - d$ to the signer, where the signer replies with $r = y - a\cdot c$ as before. Since we have $g^r \cdot h^c = Y$, the user can multiply $g^z$ and $h^d$ on each side to obtain $g^{r + z} \cdot h^{c + d} = Y'$. Thus, $\sigma ' = (c', r') \mathrel {\mathop :}=(c + d, r + z)$ is a valid signature for the message $\textsf{M}$. Moreover, it can be checked that this satisfies (perfect) blindness since any signature $\sigma ' = (c', r')$ has an equal chance of being generated from a transcript (*Y*, *c*, *r*), where the probability is taken over the randomness sampled by the user.

#### Difficulty with group actions

In the above, the user is implicitly using a specific structure of the underlying Schnorr sigma protocol to randomize the interaction. Specifically, it is using the fact that $\mathbb {G}$ is a $\mathbb {Z}_p$-*module*. This allows the user to randomize the first-signer message $Y \in \mathbb {G}$ by multiplying it with the generator $g \in \mathbb {G}$ raised to the power of $z \in \mathbb {Z}_p$ and the public key $h = g^a \in \mathbb {G}$ lifted to the power of $d \in \mathbb {Z}_p$. This property has been more formally abstracted as a *linear identification protocol* [49, 50], which covers schemes based on classical groups and lattices.

Unfortunately, this does not extend to the isogeny setting since isogenies are only a *group action*. Concretely, the CSIDH group action is defined as $*: G \times \mathcal {E}\hspace{-2.35pt}\ell \hspace{-1.30pt}\ell \rightarrow \mathcal {E}\hspace{-2.35pt}\ell \hspace{-1.30pt}\ell$, where *G* is an ideal class group and $\mathcal {E}\hspace{-2.35pt}\ell \hspace{-1.30pt}\ell$ is a set of elliptic curves, and we further assume the structure of *G* is known and can be expressed as $G=\langle [\mathfrak {g}] \rangle \cong \mathbb {Z}_N$ for some $N\in \mathbb {N}$, where $\mathfrak {g}$ is the generator [12]. Let us make an attempt to construct an isogeny-based Schnorr-style blind signature where the public key is $\textsf{pk} = A = [\mathfrak {g}^a] *E_0 \in \mathcal {E}\hspace{-2.35pt}\ell \hspace{-1.30pt}\ell$ for a random $a \overset{_{ \$}}{\leftarrow } \mathbb {Z}_N$ and a fixed curve $E_0$. While the analogy of setting the first-signer message as $Y = [\mathfrak {g}^y] *E_0$ for $y \overset{_{ \$}}{\leftarrow } \mathbb {Z}_N$ works, it seems this is as far as we can get. Unlike the Schnorr blind signature, the user can only randomize *Y* *once from the left side*. That is, while computing $[\mathfrak {g}^z] *Y$ for a random $z \in G$ is possible, combining *Y* with $[\mathfrak {g}^d] *A$ is not possible since they are both set elements. We note that in the Schnorr blind signature setting, the former and latter correspond to $g^z \cdot Y$ and $Y\cdot h^d$, respectively. Since the blindness of the Schnorr blind signature hinged on the fact that the first-sender message *Y* can be randomized *twice*; one randomness *d* to hide the challenge *c* and another randomness *z* to hide the second-signer message *r*, it is unclear how to use isogenies to construct a blind signature while having only one way to randomize *Y*.

#### Using the quadratic twist

Our main observation to overcome this problem is to rely on the property that isogenies are slightly more expressive than a group action due to the *quadratic twist*. Given any $A = [\mathfrak {g}^a] *E_0$ for an unknown $a \in \mathbb {Z}_N$, we can efficiently compute its quadratic twist $[\mathfrak {g}^{-a}] *E_0$, which we denote^3^ by $A^{-1}$.

We first explain the underlying isogeny-based sigma protocol, where we assume for now that the challenge space is $\mathcal {C} = \{ -1, 1 \}$. As above, the prover sends $Y = [\mathfrak {g}^y] *E_0$ for $y \overset{_{ \$}}{\leftarrow } \mathbb {Z}_N$. The verifier then sends a random challenge $c \overset{_{ \$}}{\leftarrow } \{ -1, 1 \}$, and the prover replies with $r = y - a \cdot c$. The verifier then verifies the “signature” $\sigma = (c, r)$ by checking whether $[\mathfrak {g}^r] *A^c = Y$, where note that $A^c$ is well-defined for $c \in \{ -1, 1 \}$ even though *A* comes from the set of elliptic curves. For an honest execution of the protocol, we have $[\mathfrak {g}^r] *A^c = [\mathfrak {g}^r] *([\mathfrak {g}^{a\cdot c}]*E_0) = [\mathfrak {g}^{r +a \cdot c}]*E_0= Y$ as desired.^4^

Our idea is to randomize this sigma protocol so that the signature $\sigma = (c, r)$ becomes $\sigma ' = (c \cdot d, r\cdot d + z )$, where (*d*, *z*) is uniform over $\{ -1, 1 \} \times \mathbb {Z}_N$ from the view of the signer. Concretely, given the first-sender message *Y*, the user randomizes *Y* by sampling random $(d, z)\overset{_{ \$}}{\leftarrow } \{ -1, 1 \} \times \mathbb {Z}_N$ and sets $Y' \mathrel {\mathop :}=[\mathfrak {g}^z] *Y^d$. It then computes $c' = \textsf{H}(Y' \Vert \textsf{M})$ and sends $c \mathrel {\mathop :}=c' \cdot d$. The signer replies with $r = y - a \cdot c$ as before. Since we have $[\mathfrak {g}^r] *A^c = Y$, the user can first compute $[\mathfrak {g}^{r \cdot d}] *A^{c \cdot d} = Y^{d}$. Namely, it performs nothing if $d = 1$, and computes the quadratic twist of both sides if $d = -1$. It then acts by $[\mathfrak {g}^z]$ to obtain $[\mathfrak {g}^{r\cdot d + z}] *A^{c \cdot d} = [\mathfrak {g}^z] *Y^d$. Since the right-hand side is $Y'$, $\sigma ' = (c', r') \mathrel {\mathop :}=(c \cdot d, r\cdot d + z )$ is a valid signature for the message $\textsf{M}$ as desired. Moreover, it can be checked that we have perfect blindness since *c* and *r* are both randomized; the (multiplicative) randomness $d \in \{ -1, 1 \}$ hides the challenge *c* and the (additive) randomness $z \in \mathbb {Z}_N$ hides the response *r*. Put differently, any signature $\sigma ' = (c', r')$ has an equal chance of being generated from a transcript (*Y*, *c*, *r*), where the probability is taken over the randomness sampled by the user.

Finally, to turn this basic idea into a secure blind signature, we enlarge the challenge space to be exponentially large, i.e., $\mathcal {C} = \{ -1, 1 \}^n$ where $n$ is the security parameter. All the above arguments naturally extend to this enlarged challenge space by running the protocol $n$ times in parallel.

#### Formal security proof

A knowledgeable reader may recall that the Schnorr blind signature is not known to be secure in the random oracle model [11]. This is also the case for our described isogeny-based blind signature. The Schnorr blind signature has been generalized by Pointcheval and Stern [71, 72] and Abe and Okamoto [4] in similar but different ways to have a security proof in the random oracle model. The latter Abe-Okamoto blind signature is compatible with our isogeny-based construction, where the public key is modified to a tuple $\textsf{pk} = (A_0, A_1) = ([\mathfrak {g}^a_0] *E_0, [\mathfrak {g}^a_1] *E_0) \in \mathcal {E}\hspace{-2.35pt}\ell \hspace{-1.30pt}\ell ^2$ for a random $(a_0, a_1) \overset{_{ \$}}{\leftarrow } \mathbb {Z}^2_N$, and the secret key to $\textsf{sk} = (\delta , a_\delta )$ for a random $\delta \overset{_{ \$}}{\leftarrow } \{ 0,1 \}$. The construction uses the OR composition of the underlying sigma protocol and works well with our idea using the quadratic twist. While the original proof of Abe and Okamoto [4] contained a subtle but non-trivially fixable bug, Kastner et al. [55] recently provided a somewhat generic proof for Abe-Okamoto style blind signatures. The security proof of our blind signature is established by adapting their result to our setting.

#### Turning it partially blind

As explained in Sect. 2.1, there is no analog of the Abe-Okamoto *partially* blind signature in the isogeny setting. The only reason why we could replicate the Abe-Okamoto (non-partial) blind signature in the isogeny setting was that both $(A_0, A_1)$ in $\textsf{pk}$ were set up in a way that the user did not know the secret exponents. Generating $A_1 \in \mathcal {E}\hspace{-2.35pt}\ell \hspace{-1.30pt}\ell$ as a hash of the tag $\textsf{info}$, i.e., $A_1 = \textsf{H}(\textsf{info})$, would have failed in the isogeny setting since we cannot do so without letting the computation of $\textsf{H}(\cdot )$ reveal the secret exponent $a_1$. If $a_1$ is public, then the scheme becomes trivially forgeable.

Our main approach in constructing a partially blind signature is to keep the same public key $\textsf{pk} = (A_0, A_1)$ as before but to generate another curve $A_2 = \textsf{H}(\textsf{info})$ *with the secret exponent* $a_2$. We then modify the signer to prove that it knows at least *two of the three* exponents of $(A_0, A_1, A_2)$. The reduction will be able to extract either a secret key pair $(a_0, a_2)$, $(a_1, a_2)$, or $(a_0, a_1)$ from the forgery: we can rely on the proof for the standard blind signature that the first two pairs occur with an almost equal probability independent of the secret key used by the reduction, and the third case always allows the reduction to win.

The question is then how to construct a base sigma protocol for this 2-out-of-3 relation that is compatible with the above randomization technique using the quadratic twist. For instance, we cannot use the well-known Cramer-Damg$\mathring{\text {a}}$rd-Schnoemakers’ sigma protocol [33] using Shamir’s secret-sharing scheme [81] since the challenge space $\mathcal {C} = \{ -1, 1 \}$ is used as a multiplicative group in our construction, rather than a field as required by Shamir’s secret-sharing scheme.^5^ To this end, we use a 2-out-of-3 *multiplicative* secret-sharing scheme as follows: Given a secret $c \in \{ -1, 1 \}$, sample $(c_0, c_1, c_2) \in \{ -1, 1 \}^3$ uniformly random conditioned on $c_0 \cdot c_1 \cdot c_2 = c$. We then view $(c_0, c_1)$, $(c_1, c_2)$, and $(c_2, c_0)$ as the three shares. One can check that any two of the three shares allow reconstructing *c*, while *c* is information-theoretically hidden when only one share is known.

We now construct a sigma protocol for a 2-out-of-3 relation using this secret-sharing scheme as follows: the high-level idea is to assign the secret shares $(c_0, c_1)$, $(c_1, c_2)$, and $(c_2, c_0)$ to the exponents $a_0$, $a_1$, and $a_2$, respectively. In more detail, assume the prover knows the exponents $a_0$ and $a_2$. It first samples two shares $(c_1, c_2) \overset{_{ \$}}{\leftarrow } \{ -1, 1 \}^2$ and runs the honest-verifier zero-knowledge simulator to simulate the knowledge of the unknown exponent $a_1$. Specifically, it samples $(r_{1, 0}, r_{1, 1}) \overset{_{ \$}}{\leftarrow } \mathbb {Z}^2_N$ and sets $(Y_{1, 0}, Y_{1, 1}) = ([\mathfrak {g}^{r_{1, 0}}] *A_1^{c_1}, [\mathfrak {g}^{r_{1, 1}}] *A_1^{c_2})$. It then sets $(Y_{b, 0}, Y_{b, 1}) = ([\mathfrak {g}^{y_{b, 0}}] *A_b, [\mathfrak {g}^{y_{b, 1}}] *A_b)$ for $b \in \{ 0, 2 \}$ by sampling the *y*’s as before. Upon receiving $(Y_{b, 0}, Y_{b, 1})_{b \in \{ 0, 1, 2 \}}$, the verifier returns a random $c \in \{ -1, 1 \}$. The prover sets the final share $c_0 = c \cdot c_1 \cdot c_2$ and computes $(r_{0, 0}, r_{0, 1}) = (y_{0, 0} - a_{0} \cdot c_{0}, y_{0, 1} - a_{0} \cdot c_{1})$ and $(r_{2, 0}, r_{2, 1}) = (y_{2, 0} - a_{2} \cdot c_{2}, y_{2, 1} - a_{2} \cdot c_{0})$, where recall $a_2$ is the publicly known exponent associated with the tag $\textsf{info}$. Finally, the prover replies with $(r_{b, 0}, r_{b, 1})_{b \in \{ 0, 1, 2 \}}$. The verifier can check the validity of the proof by a similar check as before and will be convinced that the prover knows at least two secret exponents of $\textsf{pk} = (A_0, A_1, A_2)$.

Building on a similar argument using the quadratic twist, we turn this 2-out-of-3 sigma protocol into a partially blind signature by allowing the user to appropriately randomize the first-signer message *Y*’s. The user samples three randomness from $\{ -1, 1 \}$ to randomize the challenge $(c_0, c_1, c_2)$ and six randomness from $\mathbb {Z}_N$ to randomize the second-signer message $(r_{b, 0}, r_{b, 1})_{b \in \{ 0, 1, 2 \}}$. We show that the proof of Kastner et al. [55] can be slightly modified to work for this partially blind signature.

#### Optimization using higher degree roots of unity

Finally, we show how to optimize our blind signature. One of the implicit reasons why the randomization of the sigma protocol worked was because the challenge space $\mathcal {C} = \{ -1, 1 \}$ was a multiplicative subgroup of the ring $\mathbb {Z}_N$. We generalize this observation and consider a larger challenge space $\mathcal {C}_d = \{ \zeta ^j_d \}_{j \in [d]}$, where $\zeta _d$ is the *d*-th primitive root of unity over $\mathbb {Z}_N$,^6^ i.e., $\zeta _d^d = 1$ and $\zeta _d^j \ne 1$ for any $j \in [d-1]$. $\mathcal {C}_d$ is indeed a larger multiplicative subgroup of the ring $\mathbb {Z}_N$, where setting $d = 2$ recovers the challenge space $\mathcal {C}_2 = \mathcal {C}$. The goal of the optimized scheme remains the same: we want to randomize the signature $\sigma = (c, r) \in \mathcal {C}_d \times \mathbb {Z}_N$ by $\sigma ' = (c \cdot d, r \cdot d + z)$ for a random $(d, z) \overset{_{ \$}}{\leftarrow } \mathcal {C}_d \times \mathbb {Z}_N$. However, unfortunately, when we use a larger challenge space $\mathcal {C}_d$ for $d > 2$, the underlying sigma protocol no longer satisfies correctness. Recall in the most simple sigma protocol, the verifier receives $Y = [\mathfrak {g}^y] *E_0$, outputs a challenge $c \in \{ -1, 1 \}$, receives $r = y - a \cdot c$ and checks if $[\mathfrak {g}^r] *A^c = Y$. The final check by the verifier was computable since computing the quadratic twist (i.e., $A^{-1}$) was efficient. This is no longer the case for a more general $c \in \mathcal {C}_d$ since we do not know how to compute $A^j \mathrel {\mathop :}=[\mathfrak {g}^{a\cdot \zeta ^{j}_d}] *E_0$ given only the curve $A = [\mathfrak {g}^a] *E_0 \in \mathcal {E}\hspace{-2.35pt}\ell \hspace{-1.30pt}\ell$, $j \in [d- 1]$, and $\zeta _d$ with $d \ge 3$. To this end, we extend the public key to $\textsf{pk} = (A^{j})_{j \in [d]}$ to aid the verifier’s computation and modify the sigma protocol to address this extension. This is where we rely on the new $\zeta _d$-*ring* group action inverse problem ($\zeta _d$-$\textsf{rGAIP}$) which states that given $\textsf{pk}$, it is difficult to recover the exponent $a \in \mathbb {Z}_N$. Before getting into the hardness of $\zeta _d$-$\textsf{rGAIP}$, we finish the overview of our optimized blind signature below.

Although we are now able to construct a sigma protocol with a larger challenge space, it does not yet naturally extend to blind signatures due to the extra structure. In particular, the main issue is that when the signer sends $Y = [\mathfrak {g}^y] *E_0$ as the first message, our idea was to let the user randomize this by $[\mathfrak {g}^z] *Y^{w}$, where $Y^{w} \mathrel {\mathop :}=[\mathfrak {g}^{y \cdot \zeta _d^{w}}] *E_0$ for $(z, w) \overset{_{ \$}}{\leftarrow } \mathbb {Z}_N \times \mathcal {C}_d$. However, due to the same reason as above, this cannot be efficiently computed from only *Y*. To this end, we further extend the sigma protocol so that the prover includes all $(Y^{j})_{j \in [d]}$ in the first message. While this structure cannot be efficiently checked by the verifier/user, we modify the sigma protocol so that it performs some consistency checks on these $Y^j$’s. We show that this check is sufficient to argue blindness of the resulting blind signature even when the malicious signer is using a malformed public key, i.e., $( A^j )_{j \in [d]}$ does not have the correct ring structure.

#### Cryptanalysis of $\zeta _d$-$\textsf{rGAIP}$

We have explained how to construct an optimized blind signature assuming the hardness of $\zeta _d$-$\textsf{rGAIP}$. We complement our result by providing a preliminary cryptanalysis of $\zeta _d$-$\textsf{rGAIP}$ for the CSIDH-512 parameter set. We provide an attack that exploits the additional structure of $\zeta _d$-$\textsf{rGAIP}$ for specific choices of *d*. The insight is the difference of each curves in the public key always has a factor of $(\zeta ^i_d-\zeta ^j_d)$ for distinct $i, j \in [d]$ which constitutes a non-injective endomorphism over the secret key space $\mathbb {Z}_N$. By investigating these differences, we can reduce an $\zeta _d$-$\textsf{rGAIP}$ instance to a $\textsf{GAIP}$ instance with a possibly smaller group than $\mathbb {Z}_N$ and recover partial information. Then, we can integrate these partial information in a Pohlig-Hellman sense. As a consequence, we can evaluate the upper bound security strength of $\zeta _d$-$\textsf{rGAIP}$ using known attacks against $\textsf{GAIP}$. For some choices of $\zeta _d$, $\zeta _d$-$\textsf{rGAIP}$ only has half the security of ${\textsf{GAIP}}$ for the CSIDH-512 parameters. On the other hand, for some instances of $\zeta _d$, we show that $\zeta _d$-$\textsf{rGAIP}$ is as hard as $\textsf{GAIP}$, which demonstrates that the upper bounds obtained via our cryptanalysis are also the lower bounds. There are some instances of $\zeta _d$-$\textsf{rGAIP}$ for which our attack does not apply while also having no reduction to $\textsf{GAIP}$. We leave analysis of such instances of $\zeta _d$-$\textsf{rGAIP}$ for the CSIDH-512 parameter set as an interesting future work.

#### Isogeny-based cryptography

The roots of isogeny-based cryptography can be traced back to a 1997 talk of Couveignes, later published online in 2006 [32] and independently rediscovered by Rostovstev and Stolbunov [76]. These works propose a post-quantum key establishment protocol—the CRS protocol—whose security is based on the difficulty of the “parallelization” problem for the class group action on the set of *ordinary* elliptic curves; that is, finding $[\mathfrak {a}][\mathfrak {b}] *E$ given $E, [\mathfrak {a}] *E, [\mathfrak {b}]*E$, where $E$ is an ordinary elliptic curve with endomorphism ring $\mathcal {O}$ and $[\mathfrak {a}], [\mathfrak {b}] \in \mathcal {C\ell (O)}$. This paralellization problem is the “Diffie-Hellman analogue” of the perhaps more natural “group action inversion” problem: given two ordinary curves $E$ and $E' = [\mathfrak {a}]*E$, find $[\mathfrak {a}]$. The CRS scheme suffered primarily from two flaws: first, it was impractically slow—requiring approximately 458 s to establish a key at the 128-bit security level [82]—and second, Childs, Jao, and Soukharev [31] demonstrated that the CRS protocol is vulnerable to a subexponential-time attack using Kuperberg’s algorithm [58], with later works [16, 19, 53] improving the attack to require only polynomial quantum space due to Regev’s improved version of Kuperberg’s algorithm [74].

These problems with ordinary isogeny-based protocols led researchers to instead consider protocols based on *supersingular* elliptic curves. The first such protocol was the hash function due to Charles et al. [26]. Later, De Feo, Jao, and Plût introduced the Supersingular Isogeny Diffie–Hellman (SIDH) key establishment protocol, which was later used to construct Supersingular Isogeny Key Establishment (SIKE) [52], which was a fourth round candidate in the NIST Post-Quantum Cryptography competition. Despite passive attacks on “unbalanced” variants [69, 73] and active attacks on static/ephemeral implementations [38, 45, 48], SIDH resisted cryptanalysis until 2022, when a series of papers [23, 65, 75] established that SIDH and SIKE could be broken in polynomial time. While there are proposals for countermeasures to these devastating attacks [42], the efficacy of these countermeasures has not yet been thoroughly studied.

Commutative Supersingular Isogeny Diffie-Hellman (CSIDH) was introduced in 2017 by Castryck et al. [24] as an alternative to SIDH. Unlike SIDH—which bears very little resemblance to CRS—CSIDH is very much a supersingular analogue of CRS. In CSIDH, the supersingularity of the curves involved is exploited to ensure that a torsion subgroup of very large smooth order is defined over $\mathbb {F}_{p^2}$, which allows approximately uniform random sampling and evaluation of complex multiplication to be performed very efficiently, making CSIDH orders of magnitude faster than CRS. As well, CSIDH is not known to be susceptible to any kind of adaptive attack, making it usable in the static/ephemeral setting.

The inability to uniformly sample elements of the ideal class group whose action can be computed efficiently (without knowing the relation lattice of the class group) makes it difficult to create CSIDH-based signatures. De Feo and Galbraith were the first to solve this problem in their protocol SeaSign [35], using rejection sampling to ensure that signing key information is never leaked. Later, Beullens, Kleinjung, and Vercauteren were able to compute the relation lattice of the class group used in the CSIDH-512 parameter set, and hence construct CSI-FiSh [12]: a CSIDH-based signature scheme without rejection sampling. Unfortunately, the best known classical algorithms to compute the relation lattice scale subexponentially in the CSIDH security parameter, and so it is not currently possible to extend CSI-FiSh to larger parameter sets. However, there are efficient quantum algorithms to compute these relation lattices, making CSI-FiSh a candidate for *post-post-quantum* cryptography [34]: cryptographic protocols which require a quantum computer to establish global parameters, but which are otherwise classical. A very recent work [39] shows a feasible manner to obtain the group structure using the oriented supersingular curves and imaginary quadratic orders with a large prime conductor. Though the isogeny evaluation has subexponential complexity in theory, they show a feasible result in practice by carefully choosing the parameters.

When the relation lattice of the class group is known, complex multiplication is an instance of what Couveignes [32] called a *hard homogeneous space*, and what is now often called a *cryptographic group action* [6]. While many CSIDH/CSI-FiSh-based protocols have been constructed using the group action abstractly, the CSIDH group action actually has slightly more structure than an abstract cryptographic group action. In particular, if $E/\mathbb {F}_p :y^2 = x^3 + Ax^2 +x$ has endomorphism ring $\mathcal {O}$ and $[\mathfrak {b}] \in \mathcal {C\ell (O)}$

$$\begin{aligned} ([\mathfrak {b}] *E)^{-1} = [\mathfrak {b}]^{-1} *E^{-1} \end{aligned}$$

where $E^{-1}$ has Montgomery form $E^{-1}:y^2 = x^3 - Ax^2 + x$. In particular, if we take $E = E_0 :y^2 = x^3 + x$ we have $([\mathfrak {b}] *E_0)^{-1} = [\mathfrak {b}]^{-1} *E_0$, and so given $[\mathfrak {b}] *E_0$, we have an efficient way of constructing $[\mathfrak {b}]^{-1} *E_0$. This additional structure turns out to be a powerful tool, which has led to the construction of a UC-secure isogeny-based oblivious transfer [60], provably-secure isogeny-based password authenticated key establishment [1] (which had been elusive for years [10, 83]) and new techniques for fault attack-resistance of static/ephemeral CSIDH [63]. It is also a useful tool used in [12, 61] to compress the signature or the proof size.

#### Post-quantum blind signatures

The most active area of post-quantum blind signatures is those based on lattices. The first lattice-based blind signature was proposed by Rükert [77], who followed a design paradigm similar to the classical Schnorr or Okamoto–Schnorr blind signatures [72, 79]. This approach has been optimized in subsequent works [7–9, 62, 67], where $\mathsf {BLAZE+}$ by Alkadri et al. [8] currently stands as the most efficient proposal. However, recently, Hauck et al. [50] showed that all constructions following the blueprint of Rükert’s blind signature contain the same bug in their security proof, and provided the first *provably secure* lattice-based blind signature following a similar template with a signature size of roughly 7.9 MB.

Recently, Lyubashevsky et al. [64] constructed a novel blind signature based on a new approach using one-time signatures and OR proofs. While they can support only a bounded polynomially many signatures per public key, the signature size is small as 150 KB. In a concurrent and independent work, del Pino and Katsumata [37] and Agrawal et al. [5] showed two different methods loosely following the generic blind signature construction by Fischlin [41]. The former has a signature size of roughly 100 KB under the SIS assumption and is the first scheme to have provable security in the *quantum* random oracle model. The latter has a signature size of roughly 50 KB under a newly introduced *one-more* SIS assumption. In an independent and concurrent work to ours, Beullens et al. [15] recently took this approach one step further and constructed a lattice-based blind signature with signature size of 22 KB. The construction relies on an $\textsf{NIZK}$ for proving relations of concrete hash functions.

Finally, there are a few blind signatures based on other post-quantum assumptions. Blazy et al. [17] constructs a code-based blind signature following the generic blind signature construction by Fischlin. The other is by Petzoldt et al. [70] that constructs a multivariate-based blind signature under a non-standard unforgeability notion.

## Background

### Notation

We denote the set of natural numbers and integers by $\mathbb {N}$ and $\mathbb {Z}$, respectively. We define the ring of integers modulo *N*, i.e., $\mathbb {Z}_N$, with representatives in $[-N/2,N/2)\cap \mathbb {Z}$. For a positive integer *k*, we let [*k*] denote the set $\{1,2,\ldots ,k\}$. For a vector $\overrightarrow{h}$, $h_i$ denotes its *i*-th entry and $\overrightarrow{h}_{[i]}$ denotes the vector of its first *i*-entries. For a distribution *D*, we write $x\overset{_{ \$}}{\leftarrow } D$ to denote *x* is sampled according to *D*. For a finite set *S*, we denote $x\overset{_{ \$}}{\leftarrow } S$ to sample *x* uniformly at random over *S*. We use $\odot$ to denote the component-wise multiplication of vectors in $\mathbb {R}$. We use $\Vert$ to denote the concatenation of two strings. For an element *g* and vector $\textbf{a}= (a_1, \ldots , a_n)$, we use $g^\textbf{a}$ as a shorthand for $(g^{a_1}, \ldots , g^{a_n})$. Moreover, for any operation $*$ defined between two elements *g* and *h* and vectors $\textbf{a}= (a_1, \ldots , a_n)$ and $\textbf{b}= (b_1, \ldots , b_n)$, we use $g^\textbf{a}*h^\textbf{b}$ as a shorthand for $(g^{a_1}*h^{b_1}, \ldots , g^{a_n} *h^{b_1})$.

### (Partially) Blind signatures

We define partially blind signatures consisting of three moves, which is sufficient to capture many known protocols, e.g., [4, 54, 55]. Below, we retrieve the standard definition of (three-move) blind signatures by ignoring the tag $\textsf{info}$ or alternatively setting $\textsf{info}$ to a predefined value.

#### Definition 1

(*Partially blind signature scheme*) A three-move *partially blind signature* $\textsf{PBS}= (\textsf{PBS}.\textsf{KGen}, \textsf{PBS}.\textsf{S}, \textsf{PBS}.\textsf{U}, \textsf{PBS}.\textsf{Verify})$ with an efficiently decidable public key space $\mathcal{P}\mathcal{K}$ consists of the following PPT algorithms:

$$\textsf{PBS}.\textsf{KGen}(1^n)\rightarrow (\textsf{pk}, \textsf{sk}):$$
On input the security parameter $1^n$, the key generation algorithm outputs a pair of public and secret keys $(\textsf{pk},\textsf{sk})$.
$$\textsf{PBS}.\textsf{S}= (\textsf{PBS}.\textsf{S}_1, \textsf{PBS}.\textsf{S}_2):$$
The interactive signer algorithm consists of two phases:
  $$\textsf{PBS}.\textsf{S}_1(\textsf{sk}, \textsf{info}) \rightarrow (\textsf{state}_\textsf{S}, \rho _{\textsf{S}, 1}):$$
On input a secret key $\textsf{sk}$ and a tag $\textsf{info}$, it outputs an internal signer state $\textsf{state}_\textsf{S}$ and a first-sender message $\rho _{\textsf{S}, 1}$.^7^
  $$\textsf{PBS}.\textsf{S}_2(\textsf{state}_\textsf{S}, \rho _\textsf{U})) \rightarrow \rho _{\textsf{S}, 2}:$$
On input a signer state $\textsf{state}_\textsf{S}$ and a user message $\rho _\textsf{U}$, it outputs a second-sender message $\rho _{\textsf{S}, 2}$.
$$\textsf{PBS}.\textsf{U}= (\textsf{PBS}.\textsf{U}_1, \textsf{PBS}.\textsf{U}_2):$$
The interactive user algorithm consists of two phases:
  $$\textsf{PBS}.\textsf{U}_1( \textsf{pk}, \textsf{info}, \textsf{M}, \rho _{\textsf{S}, 1}) \rightarrow (\textsf{state}_\textsf{U}, \rho _\textsf{U}):$$
On input a public key $\textsf{pk} \in \mathcal{P}\mathcal{K}$, a tag $\textsf{info}$, a message $\textsf{M}$, and a first-sender message $\rho _{\textsf{S}, 1}$, it outputs an internal user state $\textsf{state}_\textsf{U}$ and a user message $\rho _\textsf{U}$.
  $$\textsf{PBS}.\textsf{U}_2(\textsf{state}_\textsf{U}, \rho _{\textsf{S}, 2})) \rightarrow \sigma :$$
On input a user state $\textsf{state}_\textsf{U}$ and a second-signer message $\rho _{\textsf{S}, 2}$, it outputs a signature $\sigma$.
$$\textsf{PBS}.\textsf{Verify}(\textsf{pk}, \textsf{info}, \textsf{M}, \sigma ) \rightarrow 1 \text { or } 0:$$
In input a public key $\textsf{pk}$, a tag $\textsf{info}$, a message $\textsf{M}$, and a signature $\sigma$, the verification algorithm outputs 1 to indicate the signature is valid, and 0 otherwise.

If the partially blind signature only accepts a unique tag $\textsf{info}$, we drop the “partially” and simply call it a *blind signature* ($\textsf{BS}$) and omit $\textsf{info}$ from the syntax.

We require a partially blind signature to be complete, blind against malicious signer, and one-more unforgeable. We first define correctness.

#### Definition 2

(*Perfect correctness*) A three-move partially blind signature scheme $\textsf{PBS}$ is *perfectly correct* if for all public and secret key pairs $(\textsf{pk}, \textsf{sk}) \in \textsf{PBS}.\textsf{KGen}(1^n)$ and every tag and message pair $(\textsf{info}, \textsf{M})$, we have

$$\begin{aligned} \Pr \left[ \textsf{PBS}.\textsf{Verify}(\textsf{pk},\textsf{info}, \textsf{M},\sigma ) = 1 \big | \begin{array}{rl} (\textsf{state}_\textsf{S}, \rho _{\textsf{S}, 1})&{}\overset{_{ \$}}{\leftarrow } \textsf{PBS}.\textsf{S}_1(\textsf{sk},\textsf{info}) \\ (\textsf{state}_\textsf{U}, \rho _\textsf{U}) &{}\overset{_{ \$}}{\leftarrow } \textsf{PBS}.\textsf{U}_1(\textsf{pk}, \textsf{info}, \textsf{M}, \rho _{\textsf{S}, 1})\\ \rho _{\textsf{S}, 2}&{}\overset{_{ \$}}{\leftarrow } \textsf{PBS}.\textsf{S}_2(\textsf{state}_\textsf{S}, \rho _\textsf{U})\\ \sigma &{}\overset{_{ \$}}{\leftarrow } \textsf{PBS}.\textsf{U}_2(\textsf{state}_\textsf{U}, \rho _{\textsf{S}, 2}) \end{array}\right] = 1 \end{aligned}$$

The following definitions are taken from [54, 55]. Partial blindness roughly requires the transcript to be independent of the signature even if the signer choses the keys maliciously.

#### Definition 3

(*Partial blindness under chosen keys*) We define partial blindness of a three-move partially blind signature scheme $\textsf{PBS}$ via the following game between a challenger and an adversary $\mathcal {A}$:

Setup.: The challenger samples $\textsf{coin}\in \{0,1\}$ and runs $\mathcal {A}$ on input $1^n$.
Online Phase.: When $\mathcal {A}$ outputs a tag $\textsf{info}$, messages $\widetilde{\textsf{M}}_0$ and $\widetilde{\textsf{M}}_1$, and a public key $\textsf{pk} \in \mathcal{P}\mathcal{K}$, it assigns $(\textsf{M}_0, \textsf{M}_1):= (\widetilde{\textsf{M}}_\textsf{coin}, \widetilde{\textsf{M}}_{1- \textsf{coin}})$. $\mathcal {A}$ is then given access to oracles $\textsf{U}_1$, $\textsf{U}_2$, which behave as follows.
  Oracle $$\textsf{U}_1$$.
On input $b \in \{ 0,1 \}$, and a first-signer message $\rho _{{\textsf{S}, 1}, b}$, if the session *b* is not yet open, the oracle marks session *b* as opened and runs $\left( \textsf{state}_{\textsf{U}, b}, \rho _{\textsf{U}, b} \right) \overset{_{ \$}}{\leftarrow } {\textsf{PBS}.\textsf{U}}_1\left( \textsf{pk}, \textsf{info}, \textsf{M}_{b}, \rho _{{\textsf{S}, 1}, b} \right)$. It returns $\rho _{\textsf{U}, b}$ to $\mathcal {A}$.
  Oracle $$\textsf{U}_2$$.
On input $b \in \{ 0,1 \}$ and a second-signer message $\rho _{{\textsf{S}, 2}, b}$, if the session *b* is opened , the oracle creates a signature $\sigma _{b} \overset{_{ \$}}{\leftarrow } \textsf{PBS}. \textsf{U}_2\left( \textsf{state}_{\textsf{U}, b}, \rho _{{\textsf{S}, 2}, b} \right)$. It marks session *b* as closed . Oracle $\textsf{U}_2$ does not output anything.
Output Determination.: When both sessions are closed and $\textsf{PBS}.\textsf{Verify}(\textsf{pk}, \textsf{info}, \textsf{M}_{b}, \sigma _{b}) = 1$ for $b \in \{ 0,1 \}$, the oracle returns the two signatures $(\sigma _\textsf{coin}, \sigma _{1- \textsf{coin}})$ to $\mathcal {A}$, where note that $\sigma _\textsf{coin}$ (resp. $\sigma _{1- \textsf{coin}}$) is a valid signature for $\widetilde{\textsf{M}}_0$ (resp. $\widetilde{\textsf{M}}_1$) regardless of the choice of $\textsf{coin}$. $\mathcal {A}$ outputs a guess $\textsf{coin}^*$ for $\textsf{coin}$. We say $\mathcal {A}$ *wins* if $\textsf{coin}^*=\textsf{coin}$.

We say $\textsf{PBS}$ is *partially blind under chosen keys* if the advantage of $\mathcal {A}$ defined as $\Pr [\mathcal {A} \text { wins}]$ is negligible.

One-more unforgeability roughly ensures that at most one valid signature is generated after each execution of $\textsf{PBS}.\textsf{Sign}$. Formally, we have the following.

#### Definition 4

(*One-more-unforgeability*) We define $\ell$-one-more unforgeability ($\ell$-$\textsf{OMUF}$) for any $\ell \in \mathbb {N}$ of a three-move partially blind signature scheme $\textsf{PBS}$ via the following game between a challenger and an adversary $\mathcal {A}$:

Setup.: The challenger samples $(\textsf{pk},\textsf{sk})\overset{_{ \$}}{\leftarrow } \textsf{PBS}.\textsf{KGen}({1^n})$ and runs $\mathcal {A}$ on input $\textsf{pk}$. It further initializes $\ell _\textsf{closed} = 0$ and $\textsf{opened}_\textsf{sid} = \texttt {false}$ for all $\textsf{sid} \in \mathbb {N}$.
Online Phase.: $\mathcal {A}$ is given access to oracles $\textsf{S}_1$ and $\textsf{S}_2$, which behave as follows.
  Oracle $$\textsf{S}_1$$:
On input a tag $\textsf{info}$, the oracle samples a fresh session identifier $\textsf{sid}$. It sets $\textsf{opened}_\textsf{sid} \leftarrow true$ and generates $(\textsf{state}_{\textsf{S}, \textsf{sid}}, \rho _{\textsf{S}, 1})\overset{_{ \$}}{\leftarrow } \textsf{PBS}.\textsf{S}_1(\textsf{sk},\textsf{info})$. Then it returns $\textsf{sid}$ and the first-sender message $\rho _{\textsf{S}, 1}$ to $\mathcal {A}$.
  Oracle $$\textsf{S}_2$$:
On input a user message $\rho _\textsf{U}$ and a session identifier $\textsf{sid}$, if $\ell _\textsf{closed} \ge \ell$ or $\textsf{opened}_\textsf{sid} = \texttt {false}$, then it returns $\bot$. Otherwise, it sets $\ell _\textsf{closed}++$ and $\textsf{opened}_\textsf{sid}=\texttt {false}$. It then computes the second-signer message $\rho _{\textsf{S}, 2}\overset{_{ \$}}{\leftarrow } \textsf{PBS}.\textsf{S}_2(\textsf{state}_{\textsf{S}, \textsf{sid}}, \rho _\textsf{U})$ and returns $\rho _{\textsf{S}, 2}$ to $\mathcal {A}$.
Output Determination.: When $\mathcal {A}$ outputs distinct tuples $(\textsf{M}_1,\sigma _1,\textsf{info}_1),\ldots ,(\textsf{M}_k,\sigma _k,\textsf{info}_k)$, we say $\mathcal {A}$ *wins* if $k\ge \ell _\textsf{closed}+1$ and for all $i\in [k]$, $\textsf{PBS}.\textsf{Verify}(\textsf{pk},\textsf{info}_i, \textsf{M}_i, \sigma _i)=1$.

We say $\textsf{PBS}$ is $\ell$-*one-more unforgeable* if the advantage of $\mathcal {A}$ defined as $\Pr [\mathcal {A} \text { wins}]$ is negligible.

### Sigma protocols

#### Definition 5

(*Sigma protocol*) A sigma protocol $\Sigma$ for an **NP** relation *R* is a three-move public-coin interactive protocol with two pairs of PPT algorithms $\textsf{P}=(\textsf{P}_1,\textsf{P}_2), \textsf{V}$ with the following flow:

- The prover on input a statement and witness pair $(\textsf{X}, \textsf{W}) \in R$, runs $(\textsf{com}, \textsf{state}) \overset{_{ \$}}{\leftarrow } \textsf{P}_{1}(\textsf{X}, \textsf{W})$ and sends a *commitment* $\textsf{com}$ to the verifier.
- The verifier samples a random *challenge* $\textsf{ch} \overset{_{ \$}}{\leftarrow } \mathcal {C}$ from a specified challenge set, and sends $\textsf{ch}$ to the prover.
- The prover runs $\textsf{rsp} \overset{_{ \$}}{\leftarrow } \textsf{P}_{2}(\textsf{state}, \textsf{ch})$ and returns a *response* $\textsf{rsp}$ to the verifier.
- The verifier runs $\textsf{V}(\textsf{X}, \textsf{com}, \textsf{ch}, \textsf{rsp})$ and outputs 1 to indicate the prover is valid and 0 otherwise.

To be useful as an (implicit) building block for blind signatures, a sigma protocol must satisfy correctness, honest verifier zero-knowledge ($\textsf{HVZK}$), witness indistinguishability, and special soundness, defined below.

#### Definition 6

(*Perfect completeness*) A sigma protocol is *perfectly correct* if whenever the protocol is executed by an honest prover and verifier (that is, a prover and verifier who follow the specification of the protocol), the verifier will return “Accept” with probability 1.

#### Definition 7

(*Special soundness*) A sigma protocol has *special soundness* if there is an efficient (*i.e.,* polynomial-time) extractor $\textsf{Ext}$ which, given two accepting transcripts $\tau _1 = (\textsf{com}, \textsf{ch}_1, \textsf{rsp}_1)$ and $\tau _2 = (\textsf{com}, \textsf{ch}_2, \textsf{rsp}_2)$ for the same public key $\textsf{X}$, with $\textsf{ch}_1 \ne \textsf{ch}_2$, produces a witness $\textsf{W}$ to the statement $\textsf{X}$.

#### Definition 8

(*Honest verifier zero-knowledge*) A sigma protocol is *honest verifier zero-knowledge* (HVZK) if there is an efficient algorithm $\textsf{Sim}$—the *simulator*—which, given a statement $\textsf{X}$ outputs a transcript $\tau = (\textsf{com}, \textsf{ch}, \textsf{rsp})$ such that the distribution of outputs of $\textsf{Sim}$ is identical to the distribution of transcripts of honest executions of the protocol.

*Witness indistinguishability* is a weaker notion compared with $\textsf{HVZK}$, where we require the interactions between a prover using a witness $\textsf{W}_1$ or $\textsf{W}_2$ satisfying $(\textsf{X}, \textsf{W}_1), (\textsf{X}, \textsf{W}_2) \in R$ are indistinguishable. Namely, the interaction does not leak which witness is being used.

We also define a *hard instance generator* for the **NP** relation *R* as follows.

#### Definition 9

(*Hard instance generator*) An **NP** relation *R* is associated with an *instance generator* $(\textsf{IG})$ if $\textsf{IG}$, given as input the security parameter $1^n$, outputs a statement-witness pair $(\textsf{X}, \textsf{W}) \in R$. Moreover, we say the instance generator is *hard* if the following holds for any PPT adversary $\mathcal {A}$:

$$\begin{aligned} \Pr [ (\textsf{X}, \textsf{W}) \leftarrow \textsf{IG} (1^n), \textsf{W} ' \leftarrow \mathcal {A} (\textsf{X}) : (\textsf{X}, \textsf{W} ') \in R ] = \textsf{negl}. \end{aligned}$$

### Elliptic curves and isogenies

Let *E* denote an elliptic curve over a finite field $\mathbb {F}_{p}$ with *p* a large prime, and let $\textbf{0}_{E}$ be the point at infinity on *E*. The curve *E* is called supersingular if and only if $\# E\left( \mathbb {F}_{p}\right) =p+1$. Therefore, by using point counting or Schoof’s algorithm [80], one can verify the supersingularity of a given curve efficiently. Otherwise, the curve is called ordinary curve. Given two elliptic curves *E* and $E^{\prime }$, an isogeny $\phi$ is a morphism $\phi : E \rightarrow E^{\prime }$, namely, isogeny is a map given by rational functions and it is a group homomorphism such that $\phi \left( \textbf{0}_{E}\right) =\textbf{0}_{E^{\prime }}$. An isomorphism is an isogeny whose inverse over the algebraic closure is also an isogeny and two elliptic curves are isomorphic if and only if they have the same *j*-invariant. There is a one-to-one correspondence from finite subgroups of an elliptic curve to separable isogenies from said curve, up to post-composition with isomorphisms. To be more specific, any subgroup $S \subset E\left( \mathbb {F}_{p^{k}}\right)$ determines a (separable) isogeny $\phi : E \rightarrow E^{\prime }$ with ${\text {ker}} \phi =S$, i.e. $E^{\prime }=E / S$. Given subgroup *S*, the equation for $E^{\prime }$ and the isogeny $\phi$ can be computed using Vélu’s formulae using $O\left( \# S(k \log p)^{2}\right)$ bit-operations. As a result, only those isogenies who kernels are small subgroups *S* defined over extensions $\mathbb {F}_{p^k}$ of small degree *k* can be computed efficiently.

The ring of endomorphisms ${\text {End}}(E)$ consists of all isogenies from *E* to itself, and ${\text {End}}_{p}(E)$ denotes the ring of endomorphisms defined over $\mathbb {F}_{p}$.

When $E/ \mathbb {F}_p$ is supersingular, the endomorphism ring $\text {End}_p(E)$ is isomorphic to an order $\mathcal {O}$ of the quadratic field $\mathbb {Q}(\sqrt{-p})$ [24]. We recall that an order is a subring of $\mathbb {Q}(\sqrt{-p})$, which is also a finitely-generated $\mathbb {Z}$-module containing a basis of $\mathbb {Q}(\sqrt{-p})$ as a $\mathbb {Q}$-vector space. A fractional ideal $\mathfrak {a}$ of $\mathcal {O}$ is a finitely generated $\mathcal {O}$-submodule of $\mathbb {Q}(\sqrt{-p})$. We say that $\mathfrak {a}$ is invertible if there exists another fractional ideal $\mathfrak {b}$ of $\mathcal {O}$ such that $\mathfrak {a}\mathfrak {b}=\mathcal {O}$, and that it is principal if $\mathfrak {a}=\alpha \mathcal {O}$ for some $\alpha \in \mathbb {Q}(\sqrt{-p})$. The invertible fractional ideals of $\mathcal {O}$ form an Abelian group whose quotient by the subgroup of principal fractional ideals is finite. This quotient group is called the *ideal class group* of $\mathcal {O}$, and denoted by $\mathcal {C\ell (O)}$.

The ideal class group $\mathcal {C\ell (O)}$ acts freely and transitively on the set $\mathcal {E}\hspace{-2.35pt}\ell \hspace{-1.30pt}\ell _p(\mathcal {O},\pi )$, which contains all supersingular elliptic curves *E* over $\mathbb {F}_p$-modulo isomorphisms defined over $\mathbb {F}_p$—such that there exists an isomorphism between $\mathcal {O}$ and $\text {End}_p(E)$ mapping $\sqrt{-p} \in \mathcal {O}$ to the Frobenius endomorphism $\pi :(x,y) \mapsto (x^p,y^p)$. When no confusion will arise, we will abbreviate $\mathcal {E}\hspace{-2.35pt}\ell \hspace{-1.30pt}\ell _p(\mathcal {O},\pi )$ as $\mathcal {E}\hspace{-2.35pt}\ell \hspace{-1.30pt}\ell$.

The quadratic twist of a given elliptic curve $E: y^2=f(x)$ is $E^{-1}: d y^2=f(x)$ where $d \in \mathbb {F}_p^\times {\setminus }\mathbb {F}_p^{\times 2}$. When $p=3 \bmod 4$ and $E_0$ is of *j*-invariant 1728, then $E_0$ and $E_0^{-1}$ are $\mathbb {F}_p$-isomorphic. The quadratic twist can be efficiently computed in this setting. When $p=3 \bmod 4$, the quadratic twist $E^{\prime }:-y^2=x^3+A x^2+x$ of $E_A: y^2=x^3+A x^2+x$ is $\mathbb {F}_p$-isomorphic to $E_{-A}$ by considering $(x, y) \mapsto (-x, y)$. Further, $\left( [\mathfrak {a}] * E_0\right) ^{-1}=[\mathfrak {a}]^{-1} * E_0$ for any $[\mathfrak {a}] \in \mathcal {C\ell (O)}$. Therefore, for any curve $E \in \mathcal {E}\hspace{-2.35pt}\ell \hspace{-1.30pt}\ell _p(\mathcal {O}, \pi )$, we have, by the transitivity of the action,

$$\begin{aligned} ([\mathfrak {a}] * E)^{-1}= [\mathfrak {a}]^{-1} * E^{-1}. \end{aligned}$$

#### Remark 1

Throughout the rest of the paper, we consider the underlying prime $p=3 \bmod 4$. We assume the structure of the ideal class group $G=\langle [\mathfrak {g}] \rangle \cong \mathbb {Z}_N$, justified by the Cohen-Lenstra heuristic, is known for some $N\in \mathbb {N}$ and for each $i\in [N]$ the action $[\mathfrak {g}^i] *E$ can be efficiently evaluated. The setup is justified by [12].

Let $E_0\in \mathcal {E}\hspace{-2.35pt}\ell \hspace{-1.30pt}\ell$ be the supersingular curve of *j*-invariant 1728. Our cryptosystems rely on the following assumptions.

#### Definition 10

(*Group action inverse problem (GAIP)*) Given $(E_0, E') \in \mathcal {E}\hspace{-2.35pt}\ell \hspace{-1.30pt}\ell ^2$ where $E'=[\mathfrak {g}^s] *E_0$ and $s \overset{_{ \$}}{\leftarrow } [N]$, the *group action inverse problem* is to find $[\mathfrak {g}'] \in G$ such that $[\mathfrak {g}'] *E_0 =E'$.

The problem is equivalent to finding the exponent $s \bmod N$ by considering $f(m,n)=[\mathfrak {g}^m\mathfrak {g}'^n]\star E_0$ and applying the quantum period finding algorithm.

Recall that $G\cong \mathbb {Z}_N$ and $\mathbb {Z}_N$ is a ring. We introduce a generalized version of the group action inverse problem by considering a *d*-*th primitive root of unity*, denoted by $\zeta _d$, over $\mathbb {Z}_N$ such that $\zeta ^d_d=1$ and $\zeta ^j_d \ne 1$ for any $j \in [d-1]$. We define the ring group action inverse problem with respect to $\zeta _d$ as follows.

#### Definition 11

($\zeta _d$-*Ring group action inverse problem (rGAIP)*) Given $(E_0, S) \in \mathcal {E}\hspace{-2.35pt}\ell \hspace{-1.30pt}\ell ^{d + 1}$ where $S=( [\mathfrak {g}^{s\zeta ^j_d}] *E_0 )_{j \in [d]}$, $s \overset{_{ \$}}{\leftarrow } [N]$ and $d|\lambda (N)$ (here $\lambda$ is the Carmichael function), the $\zeta _d$-*ring group action inversion problem* ($\zeta _d$-$\textsf{rGAIP}$) is to recover *s*.

When the context is clear, we may remove *d* from the subscript or remove $\zeta _d$ entirely and call it ${\textsf{rGAIP}}$ for simplicity. This problem is a generalized version of $\textsf{GAIP}$, which is a $\zeta _2$-${\textsf{rGAIP}}$ with $\zeta _2=-1$. To see this, by taking the quadratic twist of a $\textsf{GAIP}$ instance $E'=[\mathfrak {g}^s] *E_0$, we have $( E',E'^{-1} )=( [\mathfrak {g}^s] *E_0,[\mathfrak {g}^{-s}] *E_0 )$. Such a $\zeta _d$ exists if *d* is a divisor of the Carmichael function $\lambda (N)$. Concretely, if $N=\Pi p^{e_i}_i$ where $p_i$ are distinct primes, we have $\lambda (N)= \textsf{lcm}_i(\lambda (p^{e_i}_i))$ where

$$\begin{aligned} \lambda (p^{e_i}_i)= \left\{ \begin{array}{ll} \frac{1}{2}\varphi (p_i^{e_i}) &{} \text{ if } p_i=2 \wedge e_i \ge 3\\ \varphi (p_i^{e_i}) &{} \text{ otherwise } \end{array} \right. \end{aligned}$$

where $\varphi$ is the Euler phi-function. Similar to $\textsf{GAIP}$ [18, 25, 43] having polynomial-time HSP algorithms for insecure group structures, the hardness of an $\zeta _d$-$\textsf{rGAIP}$ also relies on the underlying algebraic structure and the specific choice of $\zeta _d$. In Sect. 8.2, we provide a structural analysis on the $\zeta _d$-$\textsf{rGAIP}$ for CSIDH-512 and display a few weak and hard instances depending on $\zeta _d$. We show that for some carefully-chosen *d* (depending on $N$), $\zeta _d$-$\textsf{rGAIP}$ is essentially as hard as the original $\textsf{GAIP}$.

Finally, when constructing our optimized blind signatures in Sect. 7, we require *d* to satisfy a bit more requirement other than $\zeta _d$-$\textsf{rGAIP}$ being hard. Informally, we require $\eta _d = \textsf{lcm}_{i\in [d-1]}(\textsf{gcd}(\zeta ^i_d-1,N))$ to be small for the extractor of the underlying sigma protocol to be efficient. More details can be found in Sect. 7.

## Generic proofs for blind Schnorr-type signatures

In this section, we review the recent work of Kastner et al. [55] that provided a proof of the Abe-Okamoto (partially) blind signature [4]. The original security proof of the one-more unforgeability in [4] contained a leap of logic in the security proof (i.e., the scheme was correct but the security proof was not), and Kastner, Loss, and Xu provided a somewhat generic proof that works for many of the blind Schnorr-type signatures [29].^8^ While their focus was on the scheme by Abe and Okamoto, the proof is generic enough to capture other similar schemes (see for instance [55, Appendix F] that provides a proof sketch of [2]). Indeed, the constructions we propose fall under their generic proofs as well. To this end, we extract the minimal definitions and lemmas from [55] required to argue the security of our (partially) blind signatures. Here, we note that it is likely that one can rewrite [55] in a more generic fashion by borrowing the tools from [49]. However, we chose not to for better readability and since isogenies do not naturally endow a *linear* identification scheme as required by [49]. Finally, we emphasize that while this section is not contained in Sect. 3 (i.e., Background), we do not claim any technical novelty of it.

Below, we provide a brief overview of the proof by Kastner, Loss, and Xu and then introduce the key lemmas that need to be proven in this paper to apply their proof.

### Proof overview

Loosely speaking, a blind Schnorr-type signature is a type of blind signature that builds on top of a Schnorr-type sigma protocol [78]. The signer of the blind signature is identical to the prover in a sigma protocol, while the user of the blind signature modifies the verifier in the sigma protocol by appropriately adding blindness factors. In the proof of one-more unforgeability, the adversary (i.e., a malicious user) does not care if its forgeries are blind, and thus, how the blindness is achieved can be ignored for now.

At a high level, to argue one-more unforgeability, we would like the reduction to embed a hard problem into the public key of the blind signature and appeal to the special soundness of the underlying sigma protocol to extract a solution from the forgeries. However, unlike standard Fiat–Shamir-based signatures, the reduction cannot rely on $\textsf{HVZK}$ to simulate the signatures since the challenge is under the adversary’s control. To simulate the interaction between the adversary, we thus allow the public key to have *two* valid secret keys, e.g., $(\textsf{vk} = (E_0, [\mathfrak {g}^{a_0}] *E_0, [\mathfrak {g}^{a_1}] *E_0), \textsf{sk} =(\delta , a_\delta ))$ with $\delta \in \{ 0,1 \}$. The reduction embeds a hard problem into one of the secret keys while simulating with the other secret key.

What makes the security proof of blind Schnorr-type signatures tricky is that even if the adversary’s view is independent of the secret key being used, this alone does not complete the proof. This is because to argue that the secret key extracted via the special soundness of the underlying sigma protocol is unbiased, we need to argue that the algorithm (i.e., reduction) executing the extractor of the special soundness is unbiased. While this holds for standard Fiat–Shamir based signature schemes since the reduction can invoke $\textsf{HVZK}$, this is not the case for blind signatures. As we discussed above, since the adversary chooses the challenge, the reduction can only try to invoke witness indistinguishability. However, witness indistinguishability breaks when the reduction *rewinds* the adversary since the reduction needs to simulate two transcripts using the same first commitment of the sigma protocol. Thus, the reduction is not compatible with the definition of witness indistinguishability.

That being said since the view of the adversary (in each run) is independent of the secret key being used, intuition tells us that the extraction works: the only thing that’s not working is the security proof. To overcome this issue, Kastner et al. [55] provides a detailed analysis of the probability of the reduction succeeding while implicitly relying on witness indistinguishability. We note that Abe and Okamoto [4] also rely on the same proof approach but included a subtle but non-trivially fixable flaw to compute the probability.

### Key definitions, lemmas, and theorems

We extract the minimal definitions and lemmas from [55] in a self-contained manner so that the security of our (partially) blind signatures is established through several easy-to-state lemmas. For a more full exposition, we refer the readers to [55].

#### Preparation

We first assume the adversary against the one-more unforgeability game is restricted to make only $\ell + 1$ distinct hash queries to the random oracle, where $\ell + 1$ is the number of forgeries the adversary outputs. Moreover, as with any blind Schnorr-type signature, we assume each signature in the forgery is associated with a distinct hash query.^9^ We also assume the public key of the (partially) blind signature has exactly two corresponding secret keys. More specifically, we assume the underlying sigma protocol is for the **NP** OR-relation *R* defined with respect to another **NP** relation $R'$. That is, $(\textsf{X} \mathrel {\mathop :}=(\textsf{X} '_0, \textsf{X} '_1), \textsf{W} \mathrel {\mathop :}=(\delta , \textsf{W} '_\delta )) \in R$, where $(\textsf{X} _0', \textsf{W} _0'), (\textsf{X} _1', \textsf{W} _1') \in R'$, $\textsf{X}$ is the public key and $\textsf{W}$ is the secret key. Finally, we assume the adversary’s user-message $\rho _\textsf{U}$ queried to the signing algorithm $\textsf{PBS}.\textsf{S}_2$ satisfies $\rho _\textsf{U}\in \mathcal {C}$, where $\mathcal {C}$ is the challenge space of the underlying sigma protocol for relation *R* (and $R'$).

We first define the notion of *instances*. Roughly, an instance defines the signer’s key and randomness. We present a variant of the definition of instances in [55, Definition 4] that is agnostic to the underlying sigma protocol. We provide an explicit description of instances, analogous to [55, Definition 4], when we detail our construction of (partial) blind signatures.

##### Definition 12

(*Instances*) Assume the public key of a blind Schnorr-type signature has exactly two corresponding secret keys $\textsf{sk} _0 = (0, \textsf{W} '_0)$ and $\textsf{sk} _1 = (1, \textsf{W} '_1)$. We define two types of *instances* $\textbf{I}$: A **0**-side (resp. **1**-side) instance consists of $\textsf{sk} _0$ (resp. $\textsf{sk} _1$) and the randomness used by the honest signer algorithm when the secret key is fixed to $\textsf{sk} _0$ (resp. $\textsf{sk} _1$), i.e., randomness excluding those used by the key generation algorithm.

The main argument of Kastner, Loss, and Xu boils down to arguing that the output of the extraction algorithm (i.e., forking algorithm) explained above is independent of the instances.

Let $\overrightarrow{h}$ be the vector of responses returned by the random oracle, where $|{\overrightarrow{h}}| = \ell + 1$, and let $\textsf{rand}$ be the randomness used by the one-more unforgeability adversary. We define a deterministic wrapper algorithm $\mathcal {W}$ that simulates the interaction between the signer and the adversary given input $(\textbf{I}, \textsf{rand}, \overrightarrow{h})$. $\mathcal {W}$ invokes the signer and the adversary on inputs $\textbf{I}$ and $\textsf{rand}$, respectively, and uses $\overrightarrow{h}$ to answer the random oracle queries made by the adversary. We define $\mathcal {W} (\textbf{I}, \textsf{rand}, \overrightarrow{h})$ to output $\bot$ if the adversary aborts prematurely or fails to win the one-more unforgeability game, and otherwise, output what the adversary outputs. We then define the notion of *successful tuples* as follows.

##### Definition 13

(*Successful tuples*) We define the set of *successful tuples* as follows:

$$\begin{aligned} \textsf{Succ}:=\{(\textbf{I},\textsf{rand},\overrightarrow{h}) \mid \mathcal {W} (\textbf{I},\textsf{rand},\overrightarrow{h})\ne \bot \}. \end{aligned}$$

We next define a sufficient condition to invoke the extraction algorithm of the underlying sigma protocol. This is a standard definition (often implicitly) used even for Fiat-Shamir based signatures.

##### Definition 14

(*Successful Forking* [55, Definition 7]) We say two successful input tuples $(\textbf{I},\textsf{rand},\overrightarrow{h}),(\textbf{I},\textsf{rand},\overrightarrow{h} ')\in \textsf{Succ}$ $\text {fork}$ from each other at index $i\in [\ell +1]$ if $\overrightarrow{h} _{[i-1]}=\overrightarrow{h} '_{[i-1]}$ but $h_i\ne h^\prime _i$. We denote the set of hash vector pairs $(h_i,h^\prime _i)$ such that $(\textbf{I},\textsf{rand},\overrightarrow{h}),(\textbf{I},\textsf{rand},\overrightarrow{h} ')\in \textsf{Succ}$ $\text {fork}$ at index *i* as $\text {F} _i(\textbf{I},\textsf{rand})$.

We next define the notion of transcripts. A *query transcript* denotes the user messages queried to the signer. A *full transcript* denotes the entire transcript produced by the signer and the adversary, including the final forgery.

##### Definition 15

(*Query transcript*  [55, Definition 5]) Consider the wrapper $\mathcal {W}$ running on input $(\textbf{I},\textsf{rand},\overrightarrow{h})$. The *query transcript*, denoted $\overrightarrow{e} (\textbf{I},\textsf{rand},\overrightarrow{h})$, is the vector of user message ($\rho _U$) queries made to the signing algorithm $\textsf{PBS}.\textsf{S}_2$ (simulated by $\mathcal {W}$) by the adversary, ordered by $\textsf{sid}$.

##### Definition 16

(*Full transcript*  [55, Definition 6]) Consider the wrapper $\mathcal {W}$ running on input $(\textbf{I},\textsf{rand},\overrightarrow{h})$. The *full transcript*, denoted $\textsf{trans} (\textbf{I},\textsf{rand},\overrightarrow{h})$, is the transcript produced between the signer and the adversary, i.e., all messages sent between the signer and user played by the adversary, including the forgeries.

We now define *partners*, which plays a key role in the analysis of [4, 55]. Informally, two tuples $(\textbf{I},\textsf{rand},\overrightarrow{h}),(\textbf{I},\textsf{rand},\overrightarrow{h} ')\in \textsf{Succ}$ are partners at *i* if they fork at this index *i* and produce the same query transcript. Note that this does not nencessarily imply that each tuple results in the same full transcript.

##### Definition 17

(*Partners*  [55, Definition 8]) We say two successful tuples $(\textbf{I}, rand, \overrightarrow{h}),(\textbf{I}, rand, \overrightarrow{h} ^{\prime })$ are partners at index $i \in [\ell +1]$ if the followings hold:

- $(\textbf{I}, \textsf{rand}, \overrightarrow{h})$ and $(\textbf{I}, \textsf{rand}, \overrightarrow{h} ^{\prime })$ fork at index *i*.
- $$\overrightarrow{e} (\textbf{I}, \textsf{rand}, \overrightarrow{h})=\overrightarrow{e} (\textbf{I}, \textsf{rand}, \overrightarrow{h} ^{\prime })$$

We denote the set of $(\overrightarrow{h},\overrightarrow{h} ^\prime )$ such that $(\textbf{I}, \textsf{rand}, \overrightarrow{h})$ and $(\textbf{I}, \textsf{rand}, \overrightarrow{h} ^{\prime })$ are partners at index *i* by $\text {prt}_i(\textbf{I},\textsf{rand})$.

A *triangle* is another key tool introduced in [4, 55] in order to enhance the standard forking tuples with the nice properties of partners. A triangle consists of three vectors $\overrightarrow{h}, \overrightarrow{h} ', \overrightarrow{h} ''$ such that each two vectors fork at the same index, and additionally, $(\overrightarrow{h}, \overrightarrow{h} ')$ are partners.

##### Definition 18

(*Triangles* [55, Definition 9]) A triangle at index $i \in [\ell +1]$ with respect to $\textbf{I}$, $\textsf{rand}$ is a tuple of three successful tuples in the following set:

$$\begin{aligned} \triangle _i(\textbf{I}, \textsf{rand})=\left\{ \begin{array}{c|c} ((\textbf{I},\textsf{rand}, \overrightarrow{h}),&{}{(\overrightarrow{h},\overrightarrow{h} ^{\prime })\in \text {prt}_i(\textbf{I},\textsf{rand})}\\ (\textbf{I},\textsf{rand} \overrightarrow{h} ^{\prime }),&{}{(\overrightarrow{h},\overrightarrow{h} ^{\prime \prime })\in \text {F} _i(\textbf{I},\textsf{rand})}\\ (\textbf{I},\textsf{rand} \overrightarrow{h} ^{\prime \prime }))&{}{(\overrightarrow{h} ^{\prime },\overrightarrow{h} ^{\prime \prime })\in \text {F} _i(\textbf{I},\textsf{rand})} \end{array}\right\} \end{aligned}$$

For a triangle $((\textbf{I},\textsf{rand},\overrightarrow{h}),(\textbf{I},\textsf{rand},\overrightarrow{h}^{\prime }),(\textbf{I},\textsf{rand},\overrightarrow{h}^{\prime \prime }))\in \triangle _i(\textbf{I},\textsf{rand})$, we call the pair of tuples $((\textbf{I},\textsf{rand},\overrightarrow{h}), (\textbf{I},\textsf{rand},\overrightarrow{h}^{\prime }))$ the $\text {base}$, and $((\textbf{I},\textsf{rand},\overrightarrow{h}),(\textbf{I},\textsf{rand},\overrightarrow{h}^{\prime \prime }))$ and $((\textbf{I},\textsf{rand},\overrightarrow{h}^{\prime }),(\textbf{I},\textsf{rand},\overrightarrow{h}^{\prime \prime }))$ the $\text {sides}$.

We next define a map that transforms a *b*-side instance into a $(1-b)$-side instance for $b \in \{ {\textbf {0}}, {\textbf {1}} \}$. Roughly, the map allows us to relate the number of triangles with a **0**-side instance to those with a **1**-side instance. We present a variant of the definition of instances in [55, Definition 12] that is agnostic to the underlying sigma protocol. We provide an explicit description of the map, analogous to [55, Definition 12], when we detail our construction of (partial) blind signatures.

##### Definition 19

(*Mapping instances via transcript*) For $(\textbf{I}, \textsf{rand}, \overrightarrow{h}) \in \textsf{Succ}$, we define $\Phi _{\textsf{rand}, \overrightarrow{h}}(\textbf{I})$ as a function that maps a **0**-side instance $\textbf{I}$ (resp. **1**-side instance $\textbf{I}$) to a **1**-side instance $\textbf{I} '$ (resp. **0**-side instance $\textbf{I} '$).

Finally, we formally define the witness extractor used by the reduction. We present a variant of the definition of witness extractor in [55, Definition 13] that is agnostic to the underlying sigma protocol. This is because the witness extractor’s concrete description is defined using the special soundness extractor of the underlying sigma protocol, which we will do when we detail our construction of (partial) blind signatures.

##### Definition 20

(*Witness extraction*) Fix $\textbf{I}, \textsf{rand}$ and let $\overrightarrow{h}, \overrightarrow{h} ' \in \text {F} _i(\textbf{I}, \textsf{rand})$ for some $i \in [\ell + 1]$. Moreover, denote $\sigma _i, \sigma _i'$ the signatures that correspond to $h_i$, $h_i'$, respectively. We say deterministic algorithms $(\textsf{Ext}_0, \textsf{Ext}_1)$ are *witness extractors* if $(\textsf{Ext}_0(\sigma _i, \sigma _i'), \textsf{Ext}_1(\sigma _i, \sigma _i')) \in \{ (\textsf{sk} _0, \bot ), (\bot , \textsf{sk} _1), (\textsf{sk} _0, \textsf{sk} _1) \}$.^10^ For $b \in \{ 0,1 \}$, we say that the *b*-*side witness can be extracted from* $(\textbf{I}, \textsf{rand}, \overrightarrow{h})$ *and* $(\textbf{I}, \textsf{rand}, \overrightarrow{h} ')$ *at index* *i* if $\textsf{Ext}_b(\sigma _i, \sigma _i')$ outputs $\textsf{sk} _b$.

#### Sufficient condition for one-more unforgeability

We are now prepared to formally present the main result of Kastner et al. [55]. First of all, if the map $\Phi _{\textsf{rand}, \overrightarrow{h}}$ is a bijection that preserves transcripts for any $\textsf{rand}$ and $\overrightarrow{h}$, then a partner tuple with a *b*-side instance maps to another partner tuple with a $(1 - b)$-side instance for the same $\textsf{rand}$ and $\overrightarrow{h}$ (see [55, Corollary 1 and Lemma 3]). This implies that the extracted witness from a partner tuple is independent of the reduction’s secret key. However, it is not clear if the reduction is able to obtain a partner tuple by rewinding. To this end, we use the sides of the triangle rather than the base (i.e., partner tuple) to extract a witness, where the main observation is that if a *b*-side witness can be extracted from the base of a triangle, then a *b*-side witness can be extracted from at least one of the sides. Then, we argue that the reduction having a *b*-side witness hits one corner of the base of a triangle in the first run, and then hits the top of the triangle such that it creates side with a $(1-b)$-side witness with a probability of roughly 1/2.

The main contribution of Kastner et al. [55] was to make the above high-level argument precise. Their result is mostly purely statistical and it suffices to only prove that our (partial) blind signature satisfies the following two lemmas to invoke their main theorem concerning one-more unforgeability. The first lemma shows that the blind signature is perfectly *witness indistinguishable*. This is used to establish the extracted witness from a partner tuple is independent of the reduction’s secret key.

##### Lemma 1

([55, Lemma 2]) Fix $\textsf{rand}, \overrightarrow{h}$. For all tuples $(\textbf{I}, \textsf{rand}, \overrightarrow{h})\in \textsf{Succ}$, $\Phi _{\textsf{rand}, \overrightarrow{h}}$ is a self-inverse bijection and $\textsf{trans} (\textbf{I}, \textsf{rand}, \overrightarrow{h}) = \textsf{trans} (\Phi _{\textsf{rand}, \overrightarrow{h}}(\textbf{I}), \textsf{rand}, \overrightarrow{h}).$

The second lemma states that if a witness can be extracted from a base of a triangle, then the same witness can be extracted from at least one of its sides.

##### Lemma 2

([55, Corollary 3]) Fix $\textbf{I},\textsf{rand}$ and let $(\overrightarrow{h},\overrightarrow{h} ',\overrightarrow{h} '')\in \triangle _i(\textbf{I},\textsf{rand})$, for some $i\in [\ell +1]$. If the **0**-side (**1**-side) witness can be extracted from the base $(\textbf{I},\textsf{rand}, \overrightarrow{h}),(\textbf{I},\textsf{rand},\overrightarrow{h} ')$ of the triangle at index *i*, then one can also extract the **0**-side (**1**-side) witness from at least one of the sides $(\textbf{I},\textsf{rand},\overrightarrow{h}),(\textbf{I},\textsf{rand},\overrightarrow{h} '')$ or $(\textbf{I},\textsf{rand},\overrightarrow{h}),(\textbf{I},\textsf{rand},\overrightarrow{h} '')$ at index *i*.

The following is the main theorem of Kastner et al. [55, Theorem 1] casted slightly generally to be agnostic to the underlying hardness assumption.

##### Theorem 3

Let the (partially) blind Schnorr-type signature $(\textsf{P})\textsf{BS}$ be as defined in the preparation of Sect. 4.2. In particular, assume the public key consists of two instances of the **NP** relation $R'$ generated by a corresponding hard instance generator $\textsf{IG}$ and the underlying sigma protocol has challenge space $\mathcal {C}$.

If Lemmas 1 and 2 hold, then for all $\ell \in \mathbb {N}$, if there exists an adversary $\mathcal {A}$ that makes *Q* hash queries to the random oracle and breaks the $\ell$-one more unforgeability of $(\textsf{P})\textsf{BS}$ with advantage $$\epsilon _\mathcal {A} \ge \frac{C_1}{|{\mathcal {C}}|} \cdot \left( {\begin{array}{c} Q \\ \ell + 1\end{array}}\right) $$, then there exists an algorithm $\mathcal {B}$ that breaks the hard instance generator with advantage $$\epsilon _\mathcal {B}\ge C_2 \cdot \frac{\epsilon _\mathcal {A} ^2}{\left( {\begin{array}{c}Q\\ \ell + 1\end{array}}\right) ^2 \cdot (\ell + 1)^3}$$ for some universal positive constants $C_1$ and $C_2$.

We note that Kastner, Loss, and Xu only show the above theorem for blind signatures. They then show that it can be extended to a proof for their particular partially blind signature with a loss of 1/*T*, where *T* is the number of the distinct tag $\textsf{info}$ queries by the adversary (see [55, Theorem 2]). However, as explained in the introduction, we cannot follow their approach since our partially blind signature must deviate from prior constructions. To this end, we notice that the same proofs and theorem above can be applied to the partially blind setting if the instances in Definition 12 can be defined independently from the tags $\textsf{info}$ used by the adversary. See Sect. 6 for more details.

## Constructing isogeny-based blind signatures

In this section, we provide our isogeny-based blind signature. We first explain the sigma protocol that underlies our isogeny-based blind signature and then show how to compile it into a blind signature.

### Our basic sigma protocol for isogeny knowledge

First, we introduce the basic sigma protocol that we use to construct the OR-proofs which form the basis for our blind signature in Sect. 5 and our partially blind signature in Sect. 6. Though the protocol is essentially standard, we include this discussion because this Sigma protocol is *not* simply the protocol used in CRS [32, 76] adapted to the supersingular setting (as in CSI-FiSh [12])—rather, our proof uses the quadratic twist in a fundamental way, which is necessary when constructing our signature schemes.

**Fig. 1:**
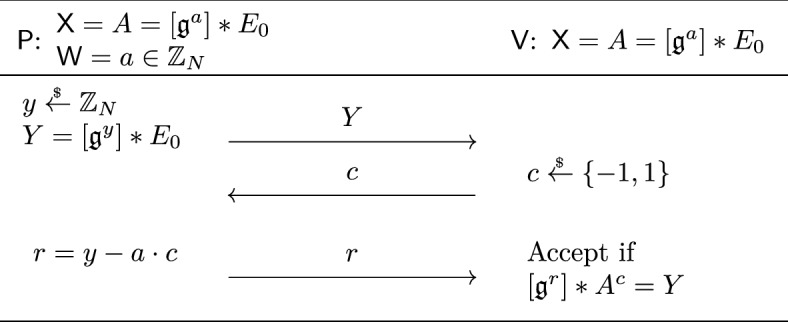
The basic Sigma protocol underlying our blind signature scheme and partially-blind signature scheme

To begin, our protocol is depicted in Fig. 1.

We prove that the scheme depicted in Fig. 1 is a secure sigma protocol; that is, that it satisfies perfect completeness, special soundess, and honest verifier zero-knowledge (HVZK).

#### Lemma 4

(Perfect completeness) The protocol depicted in Fig. 1 is perfectly complete.

#### Proof

Suppose that the protocol is executed according to the specification. Then

$$\begin{aligned}{}[\mathfrak {g}^r] *A^c = [\mathfrak {g}^{y - a\cdot c}] *[\mathfrak {g}^{a\cdot c}] *E_0 = [\mathfrak {g}^y] *E_0 = Y \end{aligned}$$

so that $\textsf{V}$ accepts, as required. $\square$

#### Lemma 5

(Special soundess) The protocol depicted in Fig. 1 satisfies special soundness.

#### Proof

Using the notation of Fig. 1, without loss of generality we may assume that $c = 1$ and $c' = -1$. Then

$$\begin{aligned} r' - r = (y + a) - (y - a) = 2a. \end{aligned}$$

Recall the parameter $p \equiv 3 \pmod {4}$ implies $|\mathcal {C\ell (O)}|$ is odd. Therefore, we can solve for the unique value of $a \in \mathbb {Z}_N$ as

$$\begin{aligned} a \equiv 2^{-1} (r' - r) \pmod {N}. \end{aligned}$$

$$\square $$

#### Lemma 6

(Honest verifier zero-knowledge) The protocol depicted in Fig. 1 satisfies the honest verifier zero-knowledge property.

#### Proof

For a fixed statement $\textsf{X} = [\mathfrak {g}^a] *E_0$, the distribution of honest transcripts is uniform on the set

1 $$\begin{aligned} T&= \{ (Y = [\mathfrak {g}^y] *E_0,\, c,\, r = y - a \cdot c) \,:\, y \in \mathbb {Z}_N, c \in \{-1,1\}\} \nonumber \\&= \{ (Y = [\mathfrak {g}^{r + ac}] *E_0,\, c,\, r) \,:\, r \in \mathbb {Z}_N, c \in \{-1,1\}\} \nonumber \\&= \{ (Y = [\mathfrak {g}^{r}] *A^c,\, c,\, r) \,:\, r \in \mathbb {Z}_n, c \in \{-1,1\}\}. \end{aligned}$$

Considering Eq. 1, we see that the following procedure will perfectly simulate the honest distribution of transcripts:

1. Choose $r \in \mathbb {Z}_N$ uniformly at random.
2. Choose $c \in \{-1,1\}$ uniformly at random.
3. Set $$Y = [g^r] *A^c$$.

Thus we have defined the required $\textsf{Sim}$, and so the protocol satisfies the honest verifier zero-knowledge property. $\square$

### Base sigma protocol for an OR relation

**Fig. 2:**
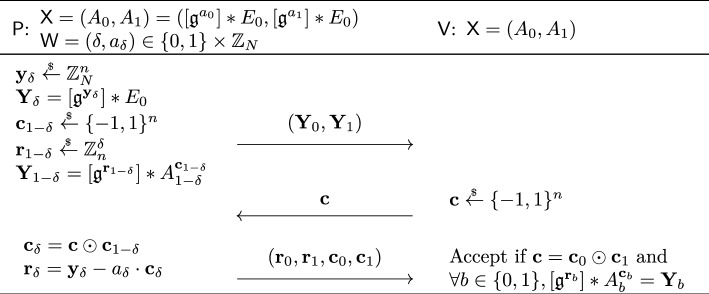
The base OR sigma protocol underlying our blind signature scheme

Building on the protocol of Sect. 5.1 we consider a sigma protocol to prove that the prover knows at least *one of the two secrets* corresponding to the public statement $\textsf{X} = (A_0, A_1) = ([\mathfrak {g}^{a_0}]*E_0, [\mathfrak {g}^{a_1}]*E_0)$. The sigma protocol is depicted in Fig. 2. Note that this is a standard isogeny-based sigma protocol where 0 is removed from the challenge space (see for instance [12]). As explained in Sect. 2.2, the main reason for this slight modification is to make the (non-soundness amplified) challenge space $\{ -1, 1 \}$ to be a (multiplicative) subgroup of $\mathbb {Z}_N^\times$.

While these properties are implicit in the blind signature, we sketch the properties of our sigma protocol for completeness. Correctness can be verified through a routine check.

#### HVZK

Given a challenge $\textbf{c}$, a zero-knowledge simulator $\textsf{Sim}$ samples random $(\textbf{c}_0, \textbf{c}_1) \overset{_{ \$}}{\leftarrow } (\{ -1, 1 \}^n)^2$ and $(\textbf{r}_0, \textbf{r}_1) \overset{_{ \$}}{\leftarrow } \mathbb {Z}_N^2$ conditioned on $\textbf{c}_0 \odot \textbf{c}_1 = \textbf{c}$. It then sets $\textbf{Y}_b = [\mathfrak {g}^{\textbf{r}_b}] *A^{\textbf{c}_b}_b$ for $b\in \{ 0,1 \}$, and outputs the simulated transcript $\big ( (\textbf{Y}_0, \textbf{Y}_1), \textbf{c}, (\textbf{r}_0, \textbf{r}_1, \textbf{c}_0, \textbf{c}_1) \big )$. Since there is a bijection between $\textbf{r}_b$ and $\textbf{Y}_b$ once $\textbf{c}_b$ is fixed, this produces a transcript identically distributed as a real transcript.

#### Witness indistinguishability

This is a direct consequence of the above since perfect $\textsf{HVZK}$ implies perfect witness indistinguishability.

#### Special soundness

Let $\big ( (\textbf{Y}_0, \textbf{Y}_1), \textbf{c}, (\textbf{r}_0, \textbf{r}_1, \textbf{c}_0, \textbf{c}_1) \big )$ and $\big ( (\textbf{Y}_0, \textbf{Y}_1), \textbf{c}', (\textbf{r}'_0, \textbf{r}'_1, \textbf{c}'_0, \textbf{c}'_1) \big )$ be two valid transcripts such that $\textbf{c}\ne \textbf{c}'$. Since $\textbf{c}\ne \textbf{c}'$, either $\textbf{c}_0 \ne \textbf{c}'_0$ or $\textbf{c}_1 \ne \textbf{c}'_1$. Without loss of generality, assume $c_{0, 1} \ne c'_{0, 1}$, where $c_{0, 1}$ and $c_{0, 1}'\in \{ -1,1 \}$ are the first elements of $\textbf{c}_0$ and $\textbf{c}_0'$, respectively. The extractor $\textsf{Ext}$ then given such two valid transcripts outputs a witness $(0, a_0 = \frac{r_{0, 1} - r_{0, 1}'}{c_{0, 1}- c_{0, 1}'})$, where $r_{0, 1}, r_{0, 1}'\in \mathbb {Z}_N$ are the first elements of $\textbf{r}_0$ and $\textbf{r}_0'$. Note that, since $p \equiv 3 \pmod {4}$, we have that $N$ is odd, so that $c_{0,1} - c'_{0,1} \in \{-2,2\}$ is invertible mod $N$. Let us verify the correctness of such an $\textsf{Ext}$. Since the two transcripts are valid, we have $[\mathfrak {g}^{r_{0, 1}}] * A_0^{c_{0, 1}} = [\mathfrak {g}^{r'_{0, 1}}] * A_0^{c'_{0, 1}}$. Plugging in $A_0 = [\mathfrak {g}^{a_0}] *E_0$, we have $[\mathfrak {g}^{r_{0, 1} + c_{0, 1}\cdot a_0}] * E_0 = [\mathfrak {g}^{r'_{0, 1} + c'_{0, 1}\cdot a_0}] * E_0$, where we use the fact $c_{0, 1}, c_{0, 1}'\in \{ -1,1 \}$. Cleaning up the exponents, we obtain the desired $a_0$.

### Description of our blind signature

**Fig. 3:**
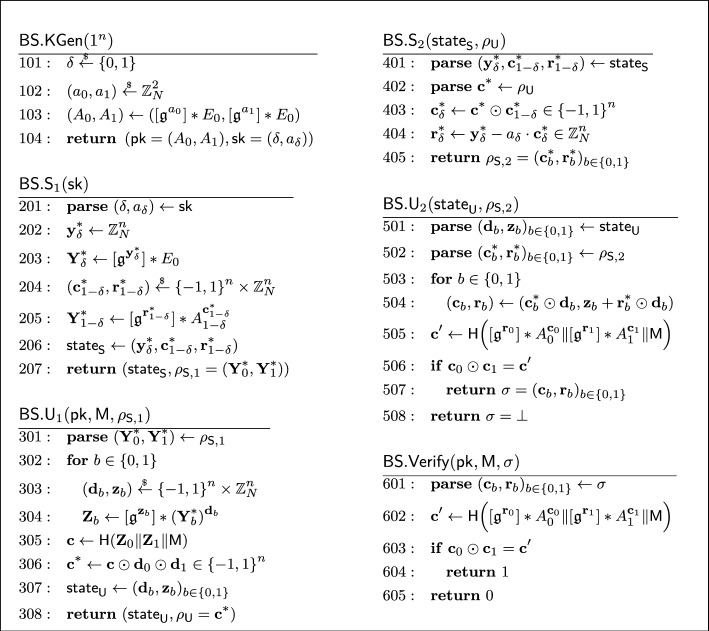
Our blind signature scheme. We assume the algorithms return $\bot$ and terminate if **parse** is not in the correct format

We present our isogeny-based blind signature building on top of the base sigma protocol in Sect. 5.2. Let $(p, N, E_0)$ be the public parameter specified as the underlying prime, the order of the group and the distinguished element, resp. Let $\mathfrak {g}$ be a generator of the ideal class group $\mathcal {C\ell (O)}$. We assume these parameters are provided to all algorithms. Let $\textsf{H}: \{0,1\}^*\rightarrow \{-1,1\}^n$ be a hash function modeled as a random oracle in the security proof.

The following algorithms are summarized in Fig. 3.

$$\textsf{BS}.\textsf{KGen}\left( 1^n\right) $$:
On input the security parameter $1^n$, it samples a bit $\delta \overset{_{ \$}}{\leftarrow } \{ 0,1 \}$, $(a_0, a_1)\overset{_{ \$}}{\leftarrow } \mathbb {Z}_N^2$ and outputs a public key $\textsf{pk} = (A_0, A_1) = ([\mathfrak {g}^{a_0}]*E_0,[\mathfrak {g}^{a_1}]*E_0)$ and secret key $\textsf{sk} = (\delta , a_\delta )$.
$$\textsf{BS}.\textsf{S}_1(\textsf{sk}):$$
The signer first samples $\textbf{y}_{\delta }^* \overset{_{ \$}}{\leftarrow } \mathbb {Z}^n_N$ and sets $\textbf{Y}_{\delta }^* = [\mathfrak {g}^{\textbf{y}_\delta ^*}] *E_0$. It then samples $(\textbf{c}^*_{1 -\delta }, \textbf{r}^*_{1 - \delta }) \overset{_{ \$}}{\leftarrow } \{ -1, 1 \}^n\times \mathbb {Z}_N^n$ and sets $\textbf{Y}_{1 - \delta }^* = [\mathfrak {g}^{\textbf{r}^*_{1 - \delta }}] *A_{1-\delta }^{\textbf{c}^*_{1 - \delta }}$. It then outputs the signer state $\textsf{state}_\textsf{S}= (\textbf{y}_\delta ^*, \textbf{c}^*_{1 -\delta }, \textbf{r}^*_{1 - \delta })$ and the first-sender message $\rho _{\textsf{S}, 1}= (\textbf{Y}_0^*, \textbf{Y}_1^*)$.
$$\textsf{BS}.\textsf{U}_1(\textsf{pk}, \textsf{M}, \rho _{\textsf{S}, 1}):$$
The user parses $(\textbf{Y}_0^*, \textbf{Y}_1^*) \leftarrow \rho _{\textsf{S}, 1}$, samples $(\textbf{d}_{b}, \textbf{z}_{b}) \overset{_{ \$}}{\leftarrow } \{ -1, 1 \}^n\times \mathbb {Z}^n_N$, and computes $\textbf{Z}_{b} = [\mathfrak {g}^{\textbf{z}_{b}}] *(\textbf{Y}^*_b)^{\textbf{d}_{b}}$ for $b\in \{ 0,1 \}$. It then computes $\textbf{c}= \textsf{H}( \textbf{Z}_0 \Vert \textbf{Z}_1 \Vert \textsf{M}) \in \{ -1, 1 \}^n$ and outputs the user state $\textsf{state}_\textsf{U}= (\textbf{d}_b, \textbf{z}_b)_{b \in \{ 0,1 \} }$ and user message $\rho _\textsf{U}= \textbf{c}^* = \textbf{c}\odot \textbf{d}_0 \odot \textbf{d}_1$.
$$\textsf{BS}.\textsf{S}_2(\textsf{state}_\textsf{S}, \rho _\textsf{U}):$$
The signer parses $(\textbf{y}_\delta ^*, \textbf{c}^*_{1 -\delta }, \textbf{r}^*_{1 - \delta }) \leftarrow \textsf{state}_\textsf{S}$, $\textbf{c}^* \leftarrow \rho _\textsf{U}$, sets $\textbf{c}_{\delta }^* = \textbf{c}^* \odot \textbf{c}_{1-\delta }^* \in \{ -1, 1 \}^n$, and computes $\textbf{r}_\delta ^* = \textbf{y}_\delta ^* - a_\delta \cdot \textbf{c}_\delta ^* \in \mathbb {Z}_N^n$.^11^ It then outputs the second-signer message $\rho _{\textsf{S}, 2}= (\textbf{c}^*_b, \textbf{r}_b^*)_{b \in \{ 0,1 \} }$.
$$\textsf{BS}.\textsf{U}_2(\textsf{state}_\textsf{U}, \rho _{\textsf{S}, 2}):$$
The user parses $(\textbf{d}_b, \textbf{z}_b)_{b \in \{ 0,1 \} } \leftarrow \textsf{state}_\textsf{U}$, $(\textbf{c}^*_b, \textbf{r}_b^*)_{b \in \{ 0,1 \} } \leftarrow \rho _{\textsf{S}, 2}$ and sets $(\textbf{c}_b, \textbf{r}_b) = (\textbf{c}^*_b \odot \textbf{d}_b, \textbf{z}_b + \textbf{r}_b^* \odot \textbf{d}_b)$ for $b \in \{ 0,1 \}$. It then checks if
  2 $$\begin{aligned} \textbf{c}_0 \odot \textbf{c}_1 = \textsf{H}\Big ( [\mathfrak {g}^{\textbf{r}_0}] *A_0^{\textbf{c}_0} \Vert [\mathfrak {g}^{\textbf{r}_1}] *A_1^{\textbf{c}_1} \Vert \textsf{M} \Big ). \end{aligned}$$
  If it holds, it outputs a signature $\sigma = (\textbf{c}_b, \textbf{r}_b)_{b \in \{ 0,1 \} } \in \big ( \{ -1, 1 \}^n\times \mathbb {Z}_N^n \big )^2$, and otherwise a $\bot$.
$$\textsf{BS}.\textsf{Verify}(\textsf{pk}, \textsf{M}, \sigma )$$:
The verifier outputs 1 if Eq. 2 holds, and otherwise 0.

The correctness, blindness, and one-more unforgeability of our blind signature are provided in the subsequent sections.

### Proof of correctness and blindness

Correctness can be checked by a routine calculation. For completeness, we provide the proof below.

#### Theorem 7

(Correctness) The blind signature scheme in Fig. 3 is (perfectly) correct.

#### Proof

To show correctness, it suffices to show that Eq. 2 holds when both the signer and user follow the protocol. First, it can be checked that we have $\textbf{Y}^*_b = [\mathfrak {g}^{\textbf{r}^*_b}] *A^{\textbf{c}^*_{b}}_b$ for $b\in \{ 0,1 \}$. The case $b = 1 - \delta$ holds by definition and the other case holds due to the correctness of the base OR sigma protocol (see Sect. 5.2). Then, substituting $(\textbf{c}_b, \textbf{r}_b) = (\textbf{c}^*_b \odot \textbf{d}_b, \textbf{z}_b + \textbf{r}_b^* \odot \textbf{d}_b)$ for $b \in \{ 0,1 \}$, we have

3 $$\begin{aligned}{}[\mathfrak {g}^{\textbf{r}_b}] *A_b^{\textbf{c}_b}&= [\mathfrak {g}^{ \textbf{z}_b + \textbf{r}_b^* \odot \textbf{d}_b }] *A_b^{\textbf{c}^*_b \odot \textbf{d}_b} \nonumber \\&= [\mathfrak {g}^{\textbf{z}_b}] *\Big ( [\mathfrak {g}^{\textbf{r}_b^* \odot \textbf{d}_b}] *A^{\textbf{c}_b^* \odot \textbf{d}_b}_b \Big ) = [\mathfrak {g}^{\textbf{z}_{b}}] *(\textbf{Y}^*_b)^{\textbf{d}_{b}} = \textbf{Z}_b. \end{aligned}$$

Finally, since $\textbf{c}= \textbf{c}^* \odot \textbf{d}_0 \odot \textbf{d}_1 = \textbf{c}^*_0 \odot \textbf{c}^*_1 \odot \textbf{d}_0 \odot \textbf{d}_1 = \textbf{c}_0 \odot \textbf{c}_1$, where $\textbf{c}= \textsf{H}( \textbf{Z}_0 \Vert \textbf{Z}_1 \Vert \textsf{M})$, we obtain Eq. 2 as desired. Note that we use the fact that $x \odot x = 1$ for any $x \in \{ -1, 1 \}$ in the first equality. $\square$

The proof of blindness is also standard. Since checking *A* is a valid elliptic curve can be done efficiently and for such valid *A*, there exists a unique $a \in \mathbb {Z}_N$ such that $[\mathfrak {g}^a] * E_0 = A$, our blind signature is secure even against a malicious server outputting an arbitrary public key.

#### Theorem 8

(Blindness) The blind signature scheme in Fig. 3 is (perfectly) blind under chosen keys.

#### Proof

It suffices to show that for any valid public key $\textsf{pk}$, any first and second-signer messages $\rho _{\textsf{S}, 1}= (\textbf{Y}_0^*, \textbf{Y}_1^*)$ and $\rho _{\textsf{S}, 2}= (\textbf{c}_b^*, \textbf{r}_b^*)_{b \in \{ 0,1 \} } \in ( \{ -1, 1 \}^n\times \mathbb {Z}_N^n )^2$, and valid signature $\sigma = (\textbf{c}_b, \textbf{r}_b)_{b \in \{ 0,1 \} } \in ( \{ -1, 1 \}^n\times \mathbb {Z}_N^n )^2$, there exists a unique and pair-wise distinct user state $\textsf{state}_\textsf{U}= (\textbf{d}_b, \textbf{z}_b)_{b \in \{ 0,1 \} } \in \big ( \{ -1, 1 \}^n\times \mathbb {Z}_N^n \big )^2$ that could have generated $\sigma$. In other words, it suffices to show that fixing an arbitrary $(\textsf{pk}, \rho _{\textsf{S}, 1}, \rho _{\textsf{S}, 2})$, there exists a bijection between a valid $\sigma$ and $\textsf{state}_\textsf{U}$. Here, note that any public key $\textsf{pk} = (A_0, A_1)$ output by the adversary (i.e., malicious signer) $\mathcal {A}$ can be efficiently checked to be valid elliptic curves (i.e., supersingularity). Below, we let $(a_0, a_1) \in \mathbb {Z}_N^2$ be the unique secret key $\textsf{sk} = (a_0, a_1)$ such that $(A_0, A_1) = ([\mathfrak {g}^{a_0}] *E_0, [\mathfrak {g}^{a_1}] *E_0)$.

Let us fix $\textsf{sk} = (a_0, a_1)$ (hence $\textsf{pk}$), $\rho _{\textsf{S}, 1}= (\textbf{Y}_0^*, \textbf{Y}_1^*)$, $\rho _{\textsf{S}, 2}= (\textbf{c}_b^*, \textbf{r}_b^*)_{b \in \{ 0,1 \} }$, and a valid signature $\sigma = (\textbf{c}_b, \textbf{r}_b)_{b \in \{ 0,1 \} }$. Let us further define the user state $\textsf{state}_\textsf{U}= (\textbf{d}_b, \textbf{z}_b)_{b \in \{ 0,1 \} }$ as $\textbf{d}_b = \textbf{c}_b \odot \textbf{c}^*_b$ and $\textbf{z}_b = \textbf{r}_b - \textbf{r}^*_b \odot \textbf{d}_b$ for $b \in \{ 0,1 \}$. Following Eq. 3 from right to left, we have $\textbf{Z}_b = [\mathfrak {g}^{\textbf{r}_b}] *A_b^{\textbf{c}_b}$ for $b\in \{ 0,1 \}$. Combining this with $\sigma$ being a valid signature, we have $\textbf{c}_0 \odot \textbf{c}_1 = \textsf{H}\Big ( [\mathfrak {g}^{\textbf{r}_0}] *A_0^{\textbf{c}_0} \Vert [\mathfrak {g}^{\textbf{r}_1}] *A_1^{\textbf{c}_1} \Vert \textsf{M} \Big ) = \textsf{H}(\textbf{Z}_0 \Vert \textbf{Z}_1 \Vert \textsf{M}).$ Therefore, $\textsf{state}_\textsf{U}$ is indeed a user state that results in the valid signature $\sigma$. Moreover, for any choice of $\rho _{\textsf{S}, 2}$ and any $\sigma \ne \sigma '$, it can be checked that the corresponding user states $\textsf{state}_\textsf{U}$ and $\textsf{state}'_\textsf{U}$ defined as above are distinct. Hence, there is a bijection between a valid signature and a user state. This concludes the proof. $\square$

### Proof of one-more unforgeability

Our proof of OMUF consists of preparing the necessary tools to invoke Theorem 3. Specifically, we define instances (see Definition 12), the map $\Phi _{\textsf{rand}, \overrightarrow{h}}$ (see Definition 19), the witness extractors $(\textsf{Ext}_0, \textsf{Ext}_1)$ (see Definition 20) and prove that Lemmas 1 and 2 hold.

Below, we denote $\overrightarrow{X}$ as a shorthand for a vector $(X^{(1)}, \ldots , X^{(\ell )})$ and endow $\overrightarrow{X}$ with the same operations defined for $X^{(k)}$ by operating them component wise. Moreover, recall $\textsf{rand}$ denotes the adversary’s randomness, and $\overrightarrow{h} = (\textbf{c}^{(1)}, \ldots , \textbf{c}^{(\ell )})$ is the random oracle’s response vector conditioned on the adversary making only $\ell$ random oracle queries. Finally, once the instance, adversary’s randomness and hash output tuple $(\textbf{I}, \textsf{rand}, \overrightarrow{h})$ is fixed, the query transcript $\overrightarrow{e} (\textbf{I},\textsf{rand},\overrightarrow{h})$—the vector of user message $\rho _U$ queries made to the signing algorithm $\textsf{BS}.\textsf{S}_2$—is defined. We denote this as $\overrightarrow{\textbf{c}^*}$ below to be consistent with the notations used in our construction.

#### Preparation: instances

Let us first define the **0**-side instance $\textbf{I} _0$ and the **1**-side instance $\textbf{I} _1$. Below, we assume the adversary against the one-more unforgeability game makes $\ell$-signing queries in total.

A **0**-side instance $\textbf{I} _0 =( 0,a_0,A_1,\overrightarrow{\textbf{y}^*_0}, \overrightarrow{\textbf{c}^*_1}, \overrightarrow{\textbf{r}^*_1} )$ is defined as follows:

- $(0,a_0):$ The secret key $\textsf{sk}$ when $\delta =0$.
- $A_1:$ The part of the public key $\textsf{pk} =(A_0,A_1)$ whose secret key is unknown.
- $\textbf{y}^{*(k)}_0:$ The exponent of the commitment $\textbf{Y}_{0}^{*(k)}$ in the *k*-th ($k\in [\ell ]$) first-sender message when $\delta =0$ such that $\textbf{Y}_{0}^{*(k)}= [\mathfrak {g}^{\textbf{y}^{*(k)}_{0}}] *E_0$.
- $\textbf{c}^{*(k)}_{1}:$ The simulated challenge in the *k*-th ($k \in [\ell ]$) first-sender message when $\delta = 0$.
- $\textbf{r}^{*(k)}_{1}:$ The exponent of the commitment $\textbf{Y}^{*(k)}_{1}$ in the *k*-th ($k \in [\ell ]$) first-sender message when $\delta = 0$ such that $\textbf{Y}^{*(k)}_{1} = [\mathfrak {g}^{\textbf{r}^{*(k)}_{1}}] *A_1^{\textbf{c}^{*(k)}_{1}}$.

A **1**-side instance $\textbf{I} _1=( 1,a_1,A_0,\overrightarrow{\textbf{y}^*_1}, \overrightarrow{\textbf{c}^*_0}, \overrightarrow{\textbf{r}^*_0} )$ is defined as follows:

- $(1,a_1):$ The secret key $\textsf{sk}$ when $\delta =1$.
- $A_0:$ The part of the public key $\textsf{pk} =(A_0,A_1)$ whose secret key is unknown.
- $\textbf{y}^{*(k)}_1:$ The exponent of the commitment $\textbf{Y}_{1}^{*(k)}$ in the *k*-th ($k\in [\ell ]$) first-sender message when $\delta =0$ such that $\textbf{Y}_{1}^{*(k)}= [\mathfrak {g}^{\textbf{y}^{*(k)}_{1}}] *E_0$.
- $\textbf{c}^{*(k)}_{0}:$ The simulated challenge in the *k*-th ($k \in [\ell ]$) first-sender message when $\delta = 1$.
- $\textbf{r}^{*(k)}_{0}:$ The exponent of the commitment $\textbf{Y}^{*(k)}_{0}$ in the *k*-th ($k \in [\ell ]$) first-sender message when $\delta = 0$ such that $\textbf{Y}^{*(k)}_{0} = [\mathfrak {g}^{\textbf{r}^{*(k)}_{0}}] *A_1^{\textbf{c}^{*(k)}_{0}}$.

#### Preparation: map $\Phi _{\textsf{rand}, \overrightarrow{h}}$

We next define the map $\Phi _{\textsf{rand}, \overrightarrow{h}}$ that maps a **0**-side instance $\textbf{I} _0$ into a **1**-side instance $\textbf{I} _1$ and vice versa. Concretely, a **0**-side instance $\textbf{I} _0 =( 0,a_0,A_1,\overrightarrow{\textbf{y}^*_0}, \overrightarrow{\textbf{c}^*_1}, \overrightarrow{\textbf{r}^*_1} )$ maps to a **1**-side instance $\textbf{I} _1$ such that

$$\begin{aligned} \textbf{I} _1 = \left( \begin{array}{c c c c c c} 1,&a_1,&A_0 = [\mathfrak {g}^{a_0}] *E_0,&\overrightarrow{\textbf{y}^*_{1}} = \overrightarrow{\textbf{r}^*_{1}} + a_1 \cdot \overrightarrow{\textbf{c}^*_1},&\overrightarrow{\textbf{c}^*_0} = \overrightarrow{\textbf{c}^*} \odot \overrightarrow{\textbf{c}^*_{1}},&\overrightarrow{\textbf{r}^*_{0}}= \overrightarrow{\textbf{y}^*_{0}} - a_0 \cdot \overrightarrow{\textbf{c}^*_0} \end{array} \right) , \end{aligned}$$

where $a_1$ is such that $[\mathfrak {g}^{a_1}] *E_0 = A_1$ and recall that $\overrightarrow{\textbf{c}^*} = \overrightarrow{e} (\textbf{I} _0,\textsf{rand},\overrightarrow{h})$. On the other hand, a **1**-side instance $\textbf{I} _1 =( 1,a_1,A_0,\overrightarrow{\textbf{y}^*_1}, \overrightarrow{\textbf{c}^*_0}, \overrightarrow{\textbf{r}^*_0} )$ maps to a **0**-side instance $\textbf{I} _0$ such that

$$\begin{aligned} \textbf{I} _0 = \left( \begin{array}{c c c c c c} 0,&a_0,&A_1 = [\mathfrak {g}^{a_1}] *E_0,&\overrightarrow{\textbf{y}^*_{0}} = \overrightarrow{\textbf{r}^*_{0}} + a_0 \cdot \overrightarrow{\textbf{c}^*_0},&\overrightarrow{\textbf{c}^*_1} = \overrightarrow{\textbf{c}^*} \odot \overrightarrow{\textbf{c}^*_{0}},&\overrightarrow{\textbf{r}^*_{1}}= \overrightarrow{\textbf{y}^*_{1}} - a_1 \cdot \overrightarrow{\textbf{c}^*_1} \end{array} \right) , \end{aligned}$$

where $a_0$ is such that $[\mathfrak {g}^{a_0}] *E_0 = A_0$ and recall that $\overrightarrow{\textbf{c}^*} = \overrightarrow{e} (\textbf{I} _1,\textsf{rand},\overrightarrow{h})$.

#### Preparation: witness extractors ${(\textsf{Ext}_0, \textsf{Ext}_1)}$

**Fig. 4:**
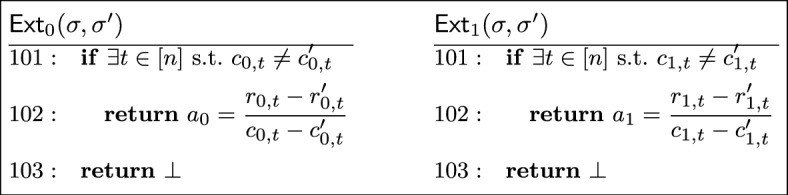
Witness extractors for our blind signature. In the above, $\sigma = (\textbf{c}_k,\textbf{r}_k)_{k\in \{0,1\}}$ and $\sigma ' = (\textbf{c}'_k,\textbf{r}'_k)_{{k\in \{0,1\}}}$, where $\textbf{c}_k$, $\textbf{c}'_k$ live in $\{ -1, 1 \}^n$ and $\textbf{r}_{k}, \textbf{r}_{k}'$ live in $\mathbb {Z}^n_N$. Non-bold font indicates the entries of a vector

Fix $\textbf{I}, \textsf{rand}$ and let $(\overrightarrow{h},\overrightarrow{h} ') \in \text {F} _i(\textbf{I},\textsf{rand})$ for some $i\in [\ell +1]$. Let us denote $\sigma = (\textbf{c}_b, \textbf{r}_b)_{b \in \{ 0,1 \} }$ and $\sigma ' = (\textbf{c}'_b, \textbf{r}'_b)_{b \in \{ 0,1 \} }$ the signatures that correspond to $\textbf{c}^{(i)}$ and $\textbf{c}'^{(i)}$, respectively, where recall $\textbf{c}^{(i)}$ (resp. $\textbf{c}'^{(i)}$) is the *i*-th entry of $\overrightarrow{h}$ (resp. $\overrightarrow{h} '$). In particular, we have $\textbf{c}_0 \odot \textbf{c}_1 = \textbf{c}^{(i)}$ and $\textbf{c}'_0 \odot \textbf{c}'_1= \textbf{c}'^{(i)}$. We define the witness extractors $(\textsf{Ext}_0, \textsf{Ext}_1)$ as in Fig. 4.

The following lemma establishes the correctness of the witness extractors.

##### Lemma 9

$(\textsf{Ext}_0, \textsf{Ext}_1)$ in Fig. 4 satisfy the definition of witness extractors in Definition 20.

##### Proof

By the definition of $\text {F} _i(\textbf{I},\textsf{rand})$ (see Definition 14), we have $(\textbf{I},\textsf{rand}, \overrightarrow{h}), (\textbf{I},\textsf{rand}, \overrightarrow{h} ') \in \textsf{Succ}$ and $\textbf{c}^{(i)} \ne \textbf{c}'^{(i)}$. The former implies that the two signatures $\sigma$ and $\sigma '$ are valid. Concretely, we have

$$\begin{aligned} \textbf{c}^{(i)} = \textbf{c}_0 \odot \textbf{c}_1&= \textsf{H}\Big ( [\mathfrak {g}^{\textbf{r}_0}] *A_0^{\textbf{c}_0} \Vert [\mathfrak {g}^{\textbf{r}_1}] *A_1^{\textbf{c}_1} \Vert \textsf{M} \Big ) \\ \textbf{c}'^{(i)} = \textbf{c}_0' \odot \textbf{c}_1'&=\textsf{H}\Big ( [\mathfrak {g}^{\textbf{r}'_0}] *A_0^{\textbf{c}'_0} \Vert [\mathfrak {g}^{\textbf{r}'_1}] *A_1^{\textbf{c}'_1} \Vert \textsf{M} \Big ). \end{aligned}$$

Moreover, since $\overrightarrow{h}$ and $\overrightarrow{h} '$ agree up to the *i*-th entry and the challenger and adversary’s randomness are fixed, the input to the hash functions agree. Namely, we have

$$\begin{aligned}{}[\mathfrak {g}^{\textbf{r}_{b}}] *A_b^{\textbf{c}_{b}} = [\mathfrak {g}^{\textbf{r}'_{b}}] *A_b^{\textbf{c}'_{b}} \hbox { for}\ b\in \{ 0,1 \} ~\wedge ~ \textsf{M}= \textsf{M}'. \end{aligned}$$

Since $\textbf{c}^{(i)} \ne \textbf{c}'^{(i)}$, we must have $\textbf{c}_0 \ne \textbf{c}'_0$ or $\textbf{c}_1\ne \textbf{c}'_1$. Based on the special-soundness of the underlying sigma protocol (see Sect. 5.2), one of $\textsf{Ext}_0$ or $\textsf{Ext}_1$ always outputs a valid secret key. This completes the proof.

$$\square $$

#### Proof of one-more unforgeability

We prove the following two lemmas required to invoke the main theorem Theorem 3.

##### Lemma 10

Lemma 1 holds for our definition of the map $\Phi _{\textsf{rand}, \overrightarrow{h}}$.

##### Proof

Since the proof for the **0**-side and **1**-side instances $\textbf{I} _0$ and $\textbf{I} _1$ are analogous, we only consider the **0**-side instance. For any $\textsf{rand}, \overrightarrow{h}$, let us consider the query transcript $\overrightarrow{e} (\textbf{I} _0, \textsf{rand}, \overrightarrow{h}) = \overrightarrow{\textbf{c}^*}$, i.e., the vector of user message $\rho _U$ queries made by the adversary to the signing algorithm $\textsf{BS}.\textsf{S}_2$. Since the underlying sigma protocol is perfectly witness indistinguishable (see Sect. 5.2), for each $i \in [\ell ]$ and $\textbf{c}^{*(i)}$, there is a set of randomness that the signer with a secret key $(1, a_1)$ (i.e., a **1**-side witness) could have used to produce the same view (i.e., first and second-signer messages) to the adversary. Concretely, this set of randomness is exactly those defined by $\Phi _{\textsf{rand}, \overrightarrow{h}}(\textbf{I} _0)$. Hence, we have $\textsf{trans} (\textbf{I} _0, \textsf{rand}, \overrightarrow{h}) = \textsf{trans} (\Phi _{\textsf{rand}, \overrightarrow{h}}(\textbf{I} _0), \textsf{rand}, \overrightarrow{h})$ as desired. Moreover, it is easy to check that $\Phi _{\textsf{rand}, \overrightarrow{h}}(\Phi _{\textsf{rand}, \overrightarrow{h}}(\textbf{I} _0))$ from the definition of $\Phi _{\textsf{rand}, \overrightarrow{h}}$. Hence, it is a bijection as desired. This completes the proof. $\square$

##### Lemma 11

Lemma 2 holds for our definition of the witness extractors $(\textsf{Ext}_0,\textsf{Ext}_1)$.

##### Proof

Since the proof of **0**-side and **1**-side is analogous, we only consider the **0**-side case. We prove the lemma by contradiction. Suppose the **0**-side witness can be extracted from the base $(\textbf{I},\textsf{rand},\overrightarrow{h}),(\textbf{I},\textsf{rand},\overrightarrow{h} ')$ at index *i*, but cannot be extracted from either of the sides $(\textbf{I},\textsf{rand},\overrightarrow{h} '),(\textbf{I},\textsf{rand},\overrightarrow{h} '')$ or $(\textbf{I},\textsf{rand},\overrightarrow{h}),(\textbf{I},\textsf{rand},\overrightarrow{h} '')$. By Lemma 9, the assumption holds if and only if $\textbf{c}_{0}=\textbf{c}_{0}^{\prime \prime }$ and $\textbf{c}_{0}^\prime =\textbf{c}_{0}^{\prime \prime }$. As a result, $\textbf{c}_{0}=\textbf{c}_{0}^\prime$. By Lemma 9, the **0**-side witness cannot be extracted from $(\textbf{I},\textsf{rand},\overrightarrow{h}),(\textbf{I},\textsf{rand},\overrightarrow{h} ')$. However, this contradicts our assumption. $\square$

Combining everything together, we obtain the following.

##### Theorem 12

(One-more unforgeability) The blind signature scheme in Fig. 3 is one-more unforgeable. To be more specific, for all $\ell \in \mathbb {N}$, if there exists an adversary $\mathcal {A}$ that makes *Q* hash queries to the random oracle and breaks the $\ell$-one more unforgeability of $\textsf{BS}$ with advantage $$\epsilon _\mathcal {A} \ge \frac{C_1}{2^n} \cdot \left( {\begin{array}{c} Q \\ \ell + 1\end{array}}\right) $$, then there exists an algorithm $\mathcal {B}$ that breaks the $\textsf{GAIP}$ problem with advantage $$\epsilon _\mathcal {B}\ge C_2 \cdot \frac{\epsilon _\mathcal {A} ^2}{\left( {\begin{array}{c} Q\\ \ell + 1\end{array}}\right) ^2 \cdot (\ell + 1)^3}$$ for some universal positive constants $C_1$ and $C_2$.

##### Remark 2

Assuming that $\textsf{GAIP}$ is subexponentially hard—more precisely, assuming that no polynomial-time adversary can solve $\textsf{GAIP}$ with probability better than $2^{-\log ^{\omega (1)} n}$, where $n$ is the security parameter—this implies that the blind signature scheme of Fig. 3 is secure in the regime of poly-logarithmically-many concurrent sessions. This is because a polynomial-time adversary makes $Q = n^{O(1)} = 2^{O(\log n)}$ hash queries, and poly-logarithmically-many concurrent sessions means $\ell = \log ^{O(1)}n$. The theorem guarantees that the advantage $\epsilon _\mathcal {A}$ of a polynomial-time adversary in the one-more unforgeability game satisfies

$$\begin{aligned} \epsilon _\mathcal {A} \le \left( {\begin{array}{c}Q\\ \ell +1\end{array}}\right) \sqrt{\frac{ (\ell +1)^3 }{C_2 2^{\log ^{\omega (1)} n}}} = 2^{\log ^{O(1)}n- \log ^{\omega (1)}n} = {\textsf{negl}}(n). \end{aligned}$$

If we assume only that no polynomial-time adversary can solve $\textsf{GAIP}$ with non-negligible probability—that is, such an adversary’s success probability is $2^{-\omega (\log n)}$—then we still have security against a constant number of concurrent sessions, since with $\ell = O(1)$ we have

$$\begin{aligned} \epsilon _\mathcal {A} \le \left( {\begin{array}{c}Q\\ \ell +1\end{array}}\right) \sqrt{\frac{ (\ell +1)^3 }{C_2 2^{\omega (\log n)}}} = 2^{O(\log n) - \omega (\log n)} = {\textsf{negl}}(n). \end{aligned}$$

##### Proof

We define the hard instance generator $\textsf{IG}$ to output a ${\textsf{GAIP}}$ problem instance. Then, the proof follows from the above Lemmas 10 and by Theorem 3, i.e., the main theorem of Kastner et al. [55]. $\square$

## Extension to partially blind signatures

In this section, we provide our isogeny-based partially blind signature. We first explain the sigma protocol that underlies our isogeny-based partially blind signature and then show how to compile it into a partially blind signature.

### Base sigma protocol for a 2-out-of-3 relation

**Fig. 5:**
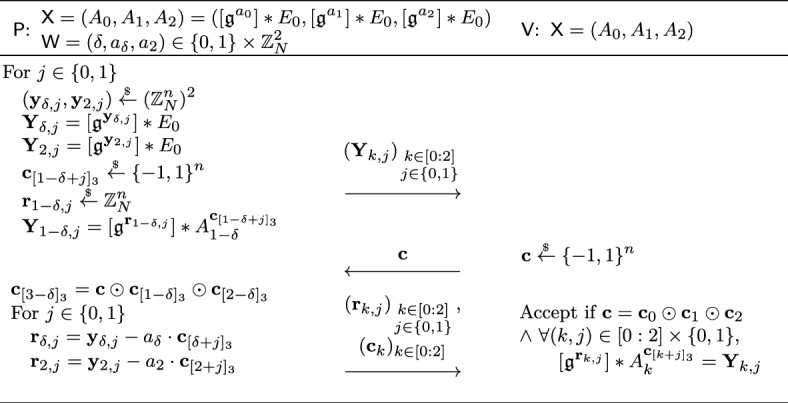
The base 2-out-of-3 sigma protocol underlying our partially blind signature scheme. Recall [0 : 2] denotes the set $\{ 0, 1, 2 \}$ and $[x]_3$ is a shorthand for $x \text { mod } 3$

We consider a sigma protocol to prove that the prover knows at least *two out of the three* secrets corresponding to the public statement $\textsf{X} = (A_0, A_1, A_2) = ([\mathfrak {g}^{a_0}]*E_0, [\mathfrak {g}^{a_1}]*E_0, [\mathfrak {g}^{a_2}]*E_0)$. The sigma protocol is depicted in Fig. 5. Since the secret $a_2$ for $A_2$ will be known by the signer *and* user in our partially blind signature, we assume the prover always knows the secret $a_2$ and proves knowledge of one other secret $a_0$ or $a_1$ in our sigma protocol.

While these properties are implicit in the partially blind signature, we sketch the properties of our sigma protocol for completeness.

#### Correctness

Observe that the prover creates six first-flow commitments $(\textbf{Y}_{k, j})_{(k, j) \in [0:2] \times \{ 0, 1 \} }$, where $(\textbf{Y}_{k, j})_{ j \in \{ 0, 1 \} }$ is used for the *k*-th statement $A_k$, and the challenges associated with $\textbf{Y}_{k, j}$ are defined as $\textbf{c}_{[k + j]_3}$. Specifically, we have the correspondence $(\textbf{Y}_{0, 0}, \textbf{Y}_{0, 1}) \mapsto (\textbf{c}_0, \textbf{c}_1)$, $(\textbf{Y}_{1, 0}, \textbf{Y}_{1, 1}) \mapsto (\textbf{c}_1, \textbf{c}_2)$, and $(\textbf{Y}_{2, 0}, \textbf{Y}_{2, 1}) \mapsto (\textbf{c}_2, \textbf{c}_0)$. Correctness then follows from a routine check.

#### $\textsf{HVZK}$

Given a challenge $\textbf{c}$, a zero-knowledge simulator $\textsf{Sim}$ samples random $(\textbf{c}_0, \textbf{c}_1, \textbf{c}_2) \overset{_{ \$}}{\leftarrow } (\{ -1, 1 \}^n)^3$ and $(\textbf{r}_{k, j})_{(k, j) \in [0:2] \times \{ 0, 1 \} }\overset{_{ \$}}{\leftarrow } \mathbb {Z}_N^6$ conditioned on $\textbf{c}_0 \odot \textbf{c}_1 \odot \textbf{c}_2 = \textbf{c}$. It then sets $\textbf{Y}_{k, j} = [\mathfrak {g}^{\textbf{r}_{k, j}}] *A^{\textbf{c}_{[k + j]_3}}_k$ for $(k, j) \in [0:2] \times \{ 0, 1 \}$, and outputs the simulated transcript $\big ( (\textbf{Y}_{k, j})_{(k, j) \in [0:2]}, \textbf{c}, ( (\textbf{r}_{k, j}, \textbf{c}_k)_{k \in [0:2]} )_{ j \in \{ 0,1 \} } \big )$. Since there is a bijection between $\textbf{r}_{k, j}$ and $\textbf{Y}_{k, j}$ once $\textbf{c}_{[k + j]_3}$ is fixed, this produces a transcript identically distributed as a real transcript.

#### Witness indistinguishability

This is a direct consequence of the above since perfect $\textsf{HVZK}$ implies perfect witness indistinguishability.

#### Special soundness

Let $\big ( (\textbf{Y}_{k, j})_{(k, j) \in [0:2]}, \textbf{c}, ( (\textbf{r}_{k, j}, \textbf{c}_k)_{k \in [0:2]} )_{ j \in \{ 0,1 \} } \big )$ and $\big ( (\textbf{Y}_{k, j})_{(k, j) \in [0:2]}, \textbf{c}', ( (\textbf{r}'_{k, j}, \textbf{c}'_k)_{k \in [0:2]} )_{ j \in \{ 0,1 \} } \big )$ be two valid transcripts such that $\textbf{c}\ne \textbf{c}'$. Since $\textbf{c}\ne \textbf{c}'$, there exists $k \in [0:2]$ such that $\textbf{c}_k \ne \textbf{c}'_k$. Without loss of generality, assume $c_{0, 1} \ne c'_{0, 1}$, where $c_{0, 1}$ and $c_{0, 1}'\in \{ -1,1 \}$ are the first elements of $\textbf{c}_0$ and $\textbf{c}_0'$, respectively. The extractor $\textsf{Ext}$ then given such two valid transcripts outputs a witness $(0, a_0 = \frac{r_{0, 0, 1} - r_{0, 0, 1}'}{c_{0, 1}- c_{0, 1}'}, a_2 = \frac{r_{2, 1, 1} - r_{2, 1, 1}'}{c_{0, 1}- c_{0, 1}'})$, where $(r_{0, 0, 1}, r'_{0, 0, 1}, r_{2, 1, 1}, r_{2, 1, 1}') \in \mathbb {Z}_N^4$ are the first elements of $(\textbf{r}_{0, 0}, \textbf{r}'_{0, 0}, \textbf{r}_{2, 1}, \textbf{r}_{2, 1}')$. Let us verify the correctness of such an $\textsf{Ext}$. Since the two transcripts are valid, we have $[\mathfrak {g}^{r_{0, 0, 1}}] * A_0^{c_{0, 1}} = [\mathfrak {g}^{r'_{0, 0, 1}}] * A_0^{c'_{0, 1}}$ and $[\mathfrak {g}^{r_{2, 1, 1}}] * A_0^{c_{0, 1}} = [\mathfrak {g}^{r'_{2, 1, 1}}] * A_0^{c'_{0, 1}}$. Plugging in $A_0 = [\mathfrak {g}^{a_0}] *E_0$ and $A_2 = [\mathfrak {g}^{a_2}] *E_0$ and following the same argument as in Sect. 5.2, we obtain the desired $(a_0, a_2)$.

### Description of our partially blind signature

**Fig. 6:**
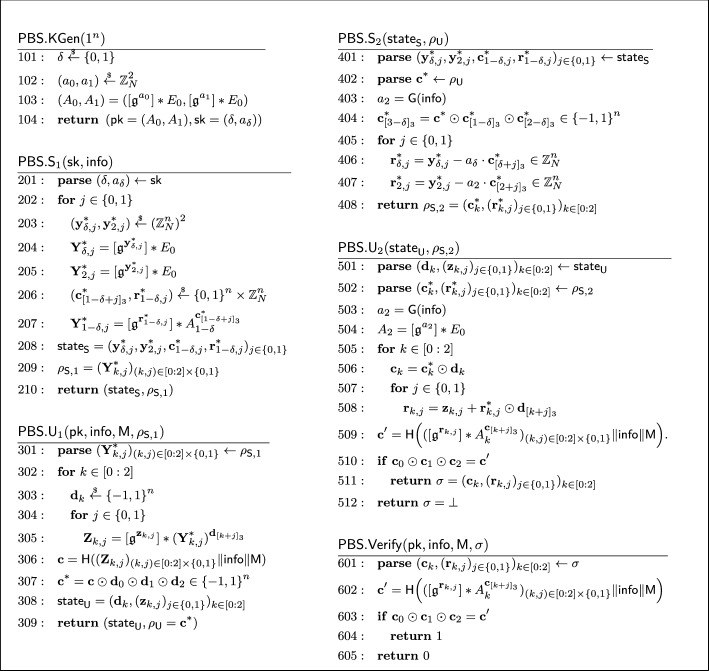
Our partially blind signature scheme. We assume the algorithms return $\bot$ and terminate if **parse** is not in the correct format. Recall [0 : 2] denotes the set $\{ 0, 1, 2 \}$ and $[x]_3$ is a shorthand for $x \text { mod } 3$

We are now able to present our isogeny-based partially blind signature. Let $( p, N, E_0)$ be the public parameters, $[\mathfrak {g}]$ be a generator in $\mathcal {C\ell (O)}$, and $\textsf{H}: \{0,1\}^*\rightarrow \{-1,1\}^n$ as defined in Sect. 5. We also require another hash function $\textsf{G}: \{ 0,1 \} ^* \rightarrow \mathbb {Z}_N$ that is modeled as a random oracle. Note that $\textsf{H}$ and $\textsf{G}$ can be implemented by a single random oracle by using domain separation. The following algorithms are summarized in Fig. 6.

$$\textsf{PBS}.\textsf{KGen}\left( 1^n\right) $$:
On input the security parameter $1^n$, it samples a bit $\delta \overset{_{ \$}}{\leftarrow } \{ 0,1 \}$, $(a_0, a_1)\overset{_{ \$}}{\leftarrow } \mathbb {Z}_N^2$ and outputs a public key $\textsf{pk} = (A_0, A_1) = ([\mathfrak {g}^{a_0}]*E_0,[\mathfrak {g}^{a_1}]*E_0)$ and secret key $\textsf{sk} = (\delta , a_\delta )$.
$$\textsf{PBS}.\textsf{S}_1(\textsf{sk}, \textsf{info}):$$
The signer performs the following for $j \in \{ 0,1 \}$: It samples $(\textbf{y}_{\delta , j}^*, \textbf{y}_{2, j}^*) \overset{_{ \$}}{\leftarrow } (\mathbb {Z}^n_N)^2$ and sets $(\textbf{Y}_{\delta , j}^*, \textbf{Y}_{2, j}^*) = ([\mathfrak {g}^{\textbf{y}^*_{\delta , j}}] *E_0, [\mathfrak {g}^{\textbf{y}^*_{2, j}}] *E_0)$. It then samples $(\textbf{c}^*_{[1 -\delta + j]_3}, \textbf{r}^*_{1 - \delta , j}) \overset{_{ \$}}{\leftarrow } \{ -1, 1 \}^n\times \mathbb {Z}_N^n$ and sets $\textbf{Y}_{1 - \delta , j}^* = [\mathfrak {g}^{\textbf{r}^*_{1 - \delta , j}}] *A_{1-\delta }^{\textbf{c}^*_{[1 - \delta + j]_3}}$. Finally, it outputs the signer state $\textsf{state}_\textsf{S}= ( \textbf{y}_{\delta , j}^*, \textbf{y}_{2, j}^*,\textbf{c}^*_{1 -\delta , j}, \textbf{r}^*_{1 - \delta , j} )_{j \in \{ 0,1 \} }$ and the first-sender message $\rho _{\textsf{S}, 1}= ( \textbf{Y}_{k, j}^* )_{(k, j) \in [0:2] \times \{ 0,1 \} }$.
$$\textsf{PBS}.\textsf{U}_1(\textsf{pk}, \textsf{info}, \textsf{M}, \rho _{\textsf{S}, 1}):$$
The user parses $( \textbf{Y}_{k, j}^* )_{(k, j) \in [0:2] \times \{ 0,1 \} } \leftarrow \rho _{\textsf{S}, 1}$. It then samples $\textbf{d}_{k} \overset{_{ \$}}{\leftarrow } \{ -1, 1 \}^n$, $\textbf{z}_{k, j} \overset{_{ \$}}{\leftarrow } \mathbb {Z}^n_N$, and computes $\textbf{Z}_{k, j} = [\mathfrak {g}^{\textbf{z}_{k, j}}] *(\textbf{Y}^*_{k, j})^{\textbf{d}_{[k + j]_3}}$ for $(k, j) \in [0:2] \times \{ 0,1 \}$. It then computes $\textbf{c}= \textsf{H}\Big ( ( \textbf{Z}_{k, j} )_{(k, j) \in [0:2] \times \{ 0,1 \} } \Vert \textsf{info}\Vert \textsf{M} \Big ) \in \{ -1, 1 \}^n$ and outputs the user state $\textsf{state}_\textsf{U}= (\textbf{d}_k, ( \textbf{z}_{k, j} )_{j \in \{ 0,1 \} })_{k \in [0:2]}$ and user message $\rho _\textsf{U}= \textbf{c}^* = \textbf{c}\odot \textbf{d}_0 \odot \textbf{d}_1\odot \textbf{d}_2$.
$$\textsf{PBS}.\textsf{S}_2(\textsf{state}_\textsf{S}, \rho _\textsf{U}):$$
The signer computes $a_2 = \textsf{G}(\textsf{info}) \in \mathbb {Z}_N$, parses $( \textbf{y}_{\delta , j}^*, \textbf{y}_{2, j}^*, \textbf{c}^*_{1 -\delta , j}, \textbf{r}^*_{1 - \delta , j} )_{j \in \{ 0,1 \} } \leftarrow \textsf{state}_\textsf{S}$, $\textbf{c}^* \leftarrow \rho _\textsf{U}$ and sets $\textbf{c}_{[ 3- \delta ]_3}^* = \textbf{c}^* \odot \textbf{c}_{[1-\delta ]_3}^*\odot \textbf{c}_{[2-\delta ]_3}^* \in \{ -1, 1 \}^n$. It then computes $\textbf{r}^*_{\delta , j} = \textbf{y}^*_{\delta , j} - a_\delta \cdot \textbf{c}^*_{[\delta + j]_3} \in \mathbb {Z}_N^n$ and $\textbf{r}^*_{2, j} = \textbf{y}^*_{2, j} - a_2 \cdot \textbf{c}^*_{[2 + j]_3} \in \mathbb {Z}_N^n$ for $j \in \{ 0,1 \}$. Finally, it outputs the second-signer message $\rho _{\textsf{S}, 2}= (\textbf{c}^*_k, ( \textbf{r}_{k, j}^* )_{j \in \{ 0,1 \} })_{k \in [0:2]}$.
$$\textsf{PBS}.\textsf{U}_2(\textsf{state}_\textsf{U}, \rho _{\textsf{S}, 2}):$$
The user first computes $a_2 = \textsf{G}(\textsf{info}) \in \mathbb {Z}_N$ and sets $A_3 = [\mathfrak {g}^{a_2}] * E_0$. It then parses $(\textbf{d}_k, ( \textbf{z}_{k, j} )_{j \in \{ 0,1 \} })_{k \in [0:2]} \leftarrow \textsf{state}_\textsf{U}$, $(\textbf{c}^*_k, ( \textbf{r}_{k, j}^* )_{j \in \{ 0,1 \} })_{k \in [0:2]} \leftarrow \rho _{\textsf{S}, 2}$ and sets $\textbf{c}_k = \textbf{c}^*_k \odot \textbf{d}_k$ and $\textbf{r}_{k, j} = \textbf{z}_{k, j} + \textbf{r}_{k, j}^* \odot \textbf{d}_{[k + j]_3}$ for $(k, j) \in [0:2] \times \{ 0,1 \}$. It then checks if
  4 $$\begin{aligned} \textbf{c}_0 \odot \textbf{c}_1 \odot \textbf{c}_2 = \textsf{H}\Big ( ( [\mathfrak {g}^{\textbf{r}_{k, j}}] *A_k^{\textbf{c}_{[k + j]_3}} )_{(k, j) \in [0:2] \times \{ 0,1 \} } \Vert \textsf{info}\Vert \textsf{M} \Big ). \end{aligned}$$
  If it holds, it outputs a signature $\sigma = (\textbf{c}_k, ( \textbf{r}_{k, j} )_{j \in \{ 0,1 \} })_{k \in [0:2]} \in \big ( \{ -1, 1 \}^n\times \big ( \mathbb {Z}_N^n \big )^2 \big )^3$, and otherwise a $\bot$.
$$\textsf{PBS}.\textsf{Verify}(\textsf{pk}, \textsf{M}, \sigma )$$:
The verifier outputs 1 if Eq. 4 holds, and otherwise 0.

The correctness, blindness, and one-more unforgeability of our blind signature are provided in the subsequent sections.

### Proof of correctness and blindness

Correctness can be checked by a routine calculation. For completeness, we provide the proof below.

#### Theorem 13

The partially blind signature scheme in Fig. 6 is (perfectly) correct.

#### Proof

To show correctness, it suffices to show that Eq. 4 holds when both the signer and user follow the protocol. First, it can be checked that we have $\textbf{Y}^*_{k, j} = [\mathfrak {g}^{\textbf{r}^*_{k, j}}] *A^{\textbf{c}^*_{[k + j]_3}}_k$ for $(k, j)\in [0:2] \times \{ 0,1 \}$. The case $k = 1 - \delta$ holds by definition and the other cases hold due to the correctness of the base 2-out-of-3 sigma protocol (see Sect. 6.1). Then, plugging in $\textbf{c}_k = \textbf{c}^*_k \odot \textbf{d}_k$ and $\textbf{r}_{k, j} = \textbf{z}_{k, j} + \textbf{r}_{k, j}^* \odot \textbf{d}_{[k + j]_3}$ for $(k, j) \in [0:2] \times \{ 0,1 \}$, we have

5 $$\begin{aligned}{}[\mathfrak {g}^{\textbf{r}_{k, j}}] *A_k^{\textbf{c}_{[k + j]_3}}&= [\mathfrak {g}^{ \textbf{z}_{k, j} + \textbf{r}_{k, j}^* \odot \textbf{d}_{[k + j]_3} }] *A_k^{\textbf{c}^*_{[k + j]_3} \odot \textbf{d}_{[k + j]_3}} \nonumber \\&= [\mathfrak {g}^{\textbf{z}_{k, j}}] *\Big ( [\mathfrak {g}^{\textbf{r}_{k, j}^* \odot \textbf{d}_{[k + j]_3}}] *A^{\textbf{c}^*_{[k + j]_3} \odot \textbf{d}_{[k + j]_3}}_k \Big ) \nonumber \\&= [\mathfrak {g}^{\textbf{z}_{k, j}}] *(\textbf{Y}^*_{k, j})^{\textbf{d}_{[k + j]_3}} = \textbf{Z}_{k, j}. \end{aligned}$$

Finally, since $\textbf{c}= \textbf{c}^* \odot \textbf{d}_0 \odot \textbf{d}_1 \odot \textbf{d}_2 = \textbf{c}^*_0 \odot \textbf{c}^*_1 \odot \textbf{c}^*_2 \odot \textbf{d}_0 \odot \textbf{d}_1 \odot \textbf{d}_2 = \textbf{c}_0 \odot \textbf{c}_1 \odot \textbf{c}_2$, where $\textbf{c}= \textsf{H}(( \textbf{Z}_{k, j} )_{(k, j) \in [0:2] \times \{ 0,1 \} } \Vert \textsf{info}\Vert \textsf{M})$, we obtain Eq. 4 as desired. Note that we use the fact that $x \odot x = 1$ for any $x \in \{ -1, 1 \}$ in the first equality. $\square$

The proof of blindness is also standard. Since checking *A* is a valid elliptic curve can be done efficiently and for such valid *A*, there exists a unique $a \in \mathbb {Z}_N$ such that $[\mathfrak {g}^a] * E_0 = A$, our partially blind signature is secure even against a malicious server outputting an arbitrary public key.

#### Theorem 14

The partially blind signature scheme in Fig. 6 is (perfectly) blind under chosen keys.

#### Proof

It suffices to show that for any valid public key $\textsf{pk}$, tag $\textsf{info}$, any first and second-signer messages $\rho _{\textsf{S}, 1}= ( \textbf{Y}_{k, j}^* )_{(k, j) \in [0:2] \times \{ 0,1 \} }$ and $\rho _{\textsf{S}, 2}= (\textbf{c}^*_k, ( \textbf{r}_{k, j}^* )_{j \in \{ 0,1 \} })_{k \in [0:2]} \in ( \{ -1, 1 \}^n\times ( \mathbb {Z}_N^{n} )^2 )^3$, and valid signature $(\textbf{c}_k, ( \textbf{r}_{k, j} )_{j \in \{ 0,1 \} })_{k \in [0:2]} \in ( \{ -1, 1 \}^n\times ( \mathbb {Z}_N^{n} )^2 )^3$, there exists a unique and pair-wise distinct user state $\textsf{state}_\textsf{U}= (\textbf{d}_k, ( \textbf{z}_{k, j} )_{j \in \{ 0,1 \} })_{k \in [0:2]} \in ( \{ -1, 1 \}^n\times ( \mathbb {Z}_N^{n} )^2 )^3$ that could have generated $\sigma$. In other words, it suffices to show that fixing an arbitrary $(\textsf{pk}, \textsf{info}, \rho _{\textsf{S}, 1}, \rho _{\textsf{S}, 2})$, there exists a bijection between a valid $\sigma$ and $\textsf{state}_\textsf{U}$. Here, note that any public key $\textsf{pk} = (A_0, A_1)$ output by the adversary (i.e., malicious signer) $\mathcal {A}$ can be efficiently checked to be valid elliptic curves (i.e., supersingularity). Below, we let $(a_0, a_1) \in \mathbb {Z}_N^2$ be the unique secret key $\textsf{sk} = (a_0, a_1)$ such that $(A_0, A_1) = ([\mathfrak {g}^{a_0}] *E_0, [\mathfrak {g}^{a_1}] *E_0)$ and set $a_2 = \textsf{G}(\textsf{info})$ and $A_2 = [\mathfrak {g}^{a_2}] *E_0$.

Let us fix $\textsf{sk} = (a_0, a_1)$ (hence $\textsf{pk}$), $(a_2, A_2)$ (hence $\textsf{info}$), $\rho _{\textsf{S}, 1}= ( \textbf{Y}_{k, j}^* )_{(k, j) \in [0:2] \times \{ 0,1 \} }$ and $\rho _{\textsf{S}, 2}= (\textbf{c}^*_k, ( \textbf{r}_{k, j}^* )_{j \in \{ 0,1 \} })_{k \in [0:2]}$, and a valid signature $\sigma = (\textbf{c}_k, ( \textbf{r}_{k, j} )_{j \in \{ 0,1 \} })_{k \in [0:2]}$. Let us further define the user state $\textsf{state}_\textsf{U}= (\textbf{d}_k, ( \textbf{z}_{k, j} )_{j \in \{ 0,1 \} })_{k \in [0:2]}$ as $\textbf{d}_k = \textbf{c}_k \odot \textbf{c}^*_k$ and $\textbf{z}_{k, j} = \textbf{r}_{k, j} - \textbf{r}^*_{k,j} \odot \textbf{d}_{[k + j]_3}$ for $(k, j) \in [0:2] \times \{ 0,1 \}$. Following Eq. 5 from right to left, we have $\textbf{Z}_{k, j} = [\mathfrak {g}^{\textbf{r}_{k, j}}] *A_k^{\textbf{c}_{[k + j]_3}}$ for $(k, j) \in [0:2] \times \{ 0,1 \}$. Combining this with $\sigma$ being a valid signature, we have $\textbf{c}_0 \odot \textbf{c}_1 \odot \textbf{c}_2 = \textsf{H}\Big ( ( [\mathfrak {g}^{\textbf{r}_{k, j}}] *A_k^{\textbf{c}_{[k + j]_3}} )_{(k, j) \in [0:2] \times \{ 0,1 \} } \Vert \textsf{info}\Vert \textsf{M} \Big ) = \textsf{H}\Big ( ( \textbf{Z}_{k, j} )_{(k, j) \in [0:2] \times \{ 0,1 \} } \Vert \textsf{info}\Vert \textsf{M} \Big ).$ Therefore, $\textsf{state}_\textsf{U}$ is indeed a user state that results in the valid signature $\sigma$. Moreover, for any choice of $\rho _{\textsf{S}, 2}$ and any $\sigma \ne \sigma '$, it can be checked that the corresponding user states $\textsf{state}_\textsf{U}$ and $\textsf{state}'_\textsf{U}$ defined as above are distinct. Hence, there is a bijection between a valid signature and a user state. This concludes the proof. $\square$

### Proof of one-more unforgeability

Our proof of OMUF consists of preparing the necessary tools to invoke Theorem 3. Specifically, we define instances (see Definition 12), the map $\Phi _{\textsf{rand}, \overrightarrow{h}}$ (see Definition 19), the witness extractors $(\textsf{Ext}_0, \textsf{Ext}_1)$ (see Definition 20) and prove that Lemmas 1 and 2 hold. We refer the readers to Sect. 5.5 for some of the notations used below.

#### Preparation: instances

Let us first define the **0**-side instance $\textbf{I} _0$ and the **1**-side instance $\textbf{I} _1$. Below, we assume the adversary against the one-more unforgeability game makes $\ell$ signing queries in total.

A **0**-side instance $\textbf{I} _0 = (0, a_0, A_1, \overrightarrow{\textbf{y}^*_{0, 0}}, \overrightarrow{\textbf{y}^*_{0, 1}}, \overrightarrow{\textbf{c}^*_{1}}, \overrightarrow{\textbf{c}^*_{2}}, \overrightarrow{\textbf{r}^*_{1, 0}}, \overrightarrow{\textbf{r}^*_{1, 1}}, \overrightarrow{\textbf{y}^*_{2, 0}}, \overrightarrow{\textbf{y}^*_{2, 1}})$ is defined as follows:

- $(0, a_0)$: The secret key $\textsf{sk}$ when $\delta = 0$.
- $A_1$: The part of the public key $\textsf{pk} = (A_0, A_1)$ whose secret key is unknown.
- $( \textbf{y}^{*(k)}_{0, 0}, \textbf{y}^{*(k)}_{0, 1} )$: The exponent of the commitment $(\textbf{Y}^{*(k)}_{0, 0}, \textbf{Y}^{*(k)}_{0, 1})$ in the *k*-th ($k \in [\ell ]$) first-sender message when $\delta = 0$ such that $(\textbf{Y}^{*(k)}_{0, 0}, \textbf{Y}^{*(k)}_{0, 1}) = ([\mathfrak {g}^{\textbf{y}^{*(k)}_{0, 0}}] *E_0, [\mathfrak {g}^{\textbf{y}^{*(k)}_{0, 1}}] *E_0)$.
- $(\textbf{c}^{*(k)}_{1}, \textbf{c}^{*(k)}_{2})$: The simulated challenge in the *k*-th ($k \in [\ell ]$) first-sender message when $\delta = 0$.
- $(\textbf{r}^{*(k)}_{1, 0}, \textbf{r}^{*(k)}_{1, 1})$: The exponent of the commitment $(\textbf{Y}^{*(k)}_{1, 0}, \textbf{Y}^{*(k)}_{1, 1})$ in the *k*-th ($k \in [\ell ]$) first-sender message when $\delta = 0$ such that $(\textbf{Y}^{*(k)}_{1, 0}, \textbf{Y}^{*(k)}_{1, 1}) = ([\mathfrak {g}^{\textbf{r}^{*(k)}_{1, 0}}] *A_1^{\textbf{c}^{*(k)}_{1}}, [\mathfrak {g}^{\textbf{r}^{*(k)}_{1, 1}}] *A_1^{\textbf{c}^{*(k)}_{2}})$.
- $( \textbf{y}^{*(k)}_{2, 0}, \textbf{y}^{*(k)}_{2, 1} )$: The exponent of the commitment $(\textbf{Y}^{*(k)}_{2, 0}, \textbf{Y}^{*(k)}_{2, 1})$ in the *k*-th ($k \in [\ell ]$) first-sender message when $\delta = 0$ such that $(\textbf{Y}^{*(k)}_{2, 0}, \textbf{Y}^{*(k)}_{2, 1}) = ([\mathfrak {g}^{\textbf{y}^{*(k)}_{2, 0}}] *E_0, [\mathfrak {g}^{\textbf{y}^{*(k)}_{2, 1}}] *E_0)$..

A **1**-side instance $\textbf{I} _1 = (1, a_1, A_0, \overrightarrow{\textbf{y}^*_{1, 0}}, \overrightarrow{\textbf{y}^*_{1, 1}}, \overrightarrow{\textbf{c}^*_{0}}, \overrightarrow{\textbf{c}^*_{1}}, \overrightarrow{\textbf{r}^*_{0, 0}}, \overrightarrow{\textbf{r}^*_{0, 1}}, \overrightarrow{\textbf{y}^*_{2, 0}}, \overrightarrow{\textbf{y}^*_{2, 1}})$ is defined as follows:

- $(1, a_1)$: The secret key $\textsf{sk}$ when $\delta = 1$.
- $A_0$: The part of the public key $\textsf{pk} = (A_0, A_1)$ whose secret key is unknown.
- $( \textbf{y}^{*(k)}_{1, 0}, \textbf{y}^{*(k)}_{1, 1} )$: The exponent of the commitment $(\textbf{Y}^{*(k)}_{1, 0}, \textbf{Y}^{*(k)}_{1, 1})$ in the *k*-th ($k \in [\ell ]$) first-sender message when $\delta = 1$ such that $(\textbf{Y}^{*(k)}_{1, 0}, \textbf{Y}^{*(k)}_{1, 1}) = ([\mathfrak {g}^{\textbf{y}^{*(k)}_{1, 0}}] *E_0, [\mathfrak {g}^{\textbf{y}^{*(k)}_{1, 1}}] *E_0)$.
- $(\textbf{c}^{*(k)}_{0}, \textbf{c}^{*(k)}_{1})$: The simulated challenge in the *k*-th ($k \in [\ell ]$) first-sender message when $\delta = 1$.
- $(\textbf{r}^{*(k)}_{0, 0}, \textbf{r}^{*(k)}_{0, 1})$: The exponent of the commitment $(\textbf{Y}^{*(k)}_{0, 0}, \textbf{Y}^{*(k)}_{0, 1})$ in the *k*-th ($k \in [\ell ]$) first-sender message when $\delta = 1$ such that $(\textbf{Y}^{*(k)}_{0, 0}, \textbf{Y}^{*(k)}_{0, 1}) = ([\mathfrak {g}^{\textbf{r}^{*(k)}_{0, 0}}] *A_0^{\textbf{c}^{*(k)}_{0}}, [\mathfrak {g}^{\textbf{r}^{*(k)}_{0, 1}}] *A_1^{\textbf{c}^{*(k)}_{1}})$.
- $( \textbf{y}^{*(k)}_{2, 0}, \textbf{y}^{*(k)}_{2, 1} )$: The exponent of the commitment $(\textbf{Y}^{*(k)}_{2, 0}, \textbf{Y}^{*(k)}_{2, 1})$ in the *k*-th ($k \in [\ell ]$) first-sender message when $\delta = 1$ such that $(\textbf{Y}^{*(k)}_{2, 0}, \textbf{Y}^{*(k)}_{2, 1}) = ([\mathfrak {g}^{\textbf{y}^{*(k)}_{2, 0}}] *E_0, [\mathfrak {g}^{\textbf{y}^{*(k)}_{2, 1}}] *E_0)$.

In the above, note that the randomness $(\overrightarrow{\textbf{y}_{2, 0}}, \overrightarrow{\textbf{y}_{2, 1}})$ associated with the tags $\overrightarrow{\textsf{info}}$ are identical for both instances, and moreover, chosen independently of the tags queried by the adversary. This will be a crucial observation when applying Theorem 3, which focuses on the one-more unforgeability of blind signatures, to the partially blind signature setting.

#### Preparation: map $\Phi _{\textsf{rand}, \overrightarrow{h}}$

We next define the map $\Phi _{\textsf{rand}, \overrightarrow{h}}$ that maps a **0**-side instance $\textbf{I} _0$ into a **1**-side instance $\textbf{I} _1$ and vice versa. Concretely, a **0**-side instance $\textbf{I} _0 = (0, a_0, A_1, \overrightarrow{\textbf{y}^*_{0, 0}}, \overrightarrow{\textbf{y}^*_{0, 1}}, \overrightarrow{\textbf{c}^*_{1}}, \overrightarrow{\textbf{c}^*_{2}}, \overrightarrow{\textbf{r}^*_{1, 0}}, \overrightarrow{\textbf{r}^*_{1, 1}}, \overrightarrow{\textbf{y}^*_{2, 0}}, \overrightarrow{\textbf{y}^*_{2, 1}})$, $\Phi _{\textsf{rand}, \overrightarrow{h}}(\textbf{I} _0)$ maps to a **1**-side instance $\textbf{I} _1$ given by

$$\begin{aligned} \textbf{I} _1 = \left( \begin{array}{ccccc} &{} a_1 \text { such that } [\mathfrak {g}^{a_1}] *E_0 = A_1, &{} A_0 = [\mathfrak {g}^{a_0}] *E_0, &{} &{}\\ 1, &{} \overrightarrow{\textbf{y}^*_{1, 0}} = \overrightarrow{\textbf{r}^*_{1, 0}} + a_1 \cdot \overrightarrow{\textbf{c}^*_1}, &{} \overrightarrow{\textbf{y}^*_{1, 1}}= \overrightarrow{\textbf{r}^*_{1, 1}} + a_1 \cdot \overrightarrow{\textbf{c}^*_2}, &{}\overrightarrow{\textbf{y}^*_{2, 0}}, &{} \overrightarrow{\textbf{y}^*_{2, 1}} \\ &{} \overrightarrow{\textbf{c}^*_0} = \overrightarrow{\textbf{c}^*} \odot \overrightarrow{\textbf{c}^*_{1}} \odot \overrightarrow{\textbf{c}^*_{2}}, &{} \overrightarrow{\textbf{c}^*_1}, &{}&{}\\ &{} \overrightarrow{\textbf{r}^*_{0, 0}} = \overrightarrow{\textbf{y}^*_{0, 0}} - a_0 \cdot \overrightarrow{\textbf{c}^*_0}, &{} \overrightarrow{\textbf{r}^*_{0, 1}}= \overrightarrow{\textbf{y}^*_{0, 1}} - a_0 \cdot \overrightarrow{\textbf{c}^*_1},&{} &{} \end{array} \right) , \end{aligned}$$

where recall that $\overrightarrow{\textbf{c}^*} = \overrightarrow{e} (\textbf{I} _0,\textsf{rand},\overrightarrow{h})$.

On the other hand, a **1**-side instance $\textbf{I} _1 = (1, a_1, A_0, \overrightarrow{\textbf{y}^*_{1, 0}}, \overrightarrow{\textbf{y}^*_{1, 1}}, \overrightarrow{\textbf{c}^*_{0}}, \overrightarrow{\textbf{c}^*_{1}}, \overrightarrow{\textbf{r}^*_{0, 0}}, \overrightarrow{\textbf{r}^*_{0, 1}}, \overrightarrow{\textbf{y}^*_{2, 0}}, \overrightarrow{\textbf{y}^*_{2, 1}})$, $\Phi _{\textsf{rand}, \overrightarrow{h}}(\textbf{I} _1)$ maps to a **0**-side instance $\textbf{I} _0$ such that

$$\begin{aligned} \textbf{I} _0 = \left( \begin{array}{ccccc} &{} a_0 \text { such that } [\mathfrak {g}^{a_0}] *E_0 = A_0, &{} A_1 = [\mathfrak {g}^{a_1}] *E_0, &{} &{}\\ 0, &{} \overrightarrow{\textbf{y}^*_{0, 0}} = \overrightarrow{\textbf{r}^*_{0, 0}} + a_0 \cdot \overrightarrow{\textbf{c}^*_0}, &{} \overrightarrow{\textbf{y}^*_{0, 1}}= \overrightarrow{\textbf{r}^*_{0, 1}} + a_0 \cdot \overrightarrow{\textbf{c}^*_1}, &{}\overrightarrow{\textbf{y}^*_{2, 0}}, &{} \overrightarrow{\textbf{y}^*_{2, 1}} \\ &{} \overrightarrow{\textbf{c}^*_1}, &{} \overrightarrow{\textbf{c}^*_2} = \overrightarrow{\textbf{c}^*} \odot \overrightarrow{\textbf{c}^*_{0}} \odot \overrightarrow{\textbf{c}^*_{1}}, &{}&{}\\ &{} \overrightarrow{\textbf{r}^*_{1, 0}} = \overrightarrow{\textbf{y}^*_{1, 0}} - a_1 \cdot \overrightarrow{\textbf{c}^*_1}, &{} \overrightarrow{\textbf{r}^*_{1, 1}}= \overrightarrow{\textbf{y}^*_{1, 1}} - a_1 \cdot \overrightarrow{\textbf{c}^*_2},&{} &{} \end{array} \right) , \end{aligned}$$

where recall that $\overrightarrow{\textbf{c}^*} = \overrightarrow{e} (\textbf{I} _1,\textsf{rand},\overrightarrow{h})$.

#### Preparation: witness extractors $(\textsf{Ext}_0, \textsf{Ext}_1)$

**Fig. 7:**
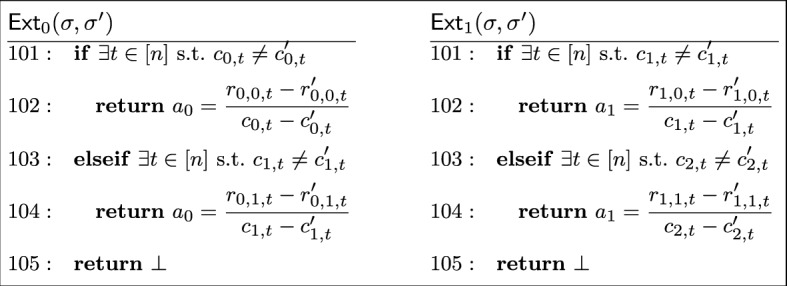
Witness extractors for our partially blind signature. In the above, $\sigma = (\textbf{c}_k, ( \textbf{r}_{k, j} )_{j \in \{ 0,1 \} })_{k \in [0:2]}$ and $\sigma ' = (\textbf{c}'_k, ( \textbf{r}'_{k, j} )_{j \in \{ 0,1 \} })_{k \in [0:2]}$, where $\textbf{c}_k$, $\textbf{c}'_k$ live in $\{ -1, 1 \}^n$ and $\textbf{r}_{k, j}, \textbf{r}_{k, j}'$ live in $\mathbb {Z}^n_N$. Non-bold font indicates the entries of a vector

Fix $\textbf{I}, \textsf{rand}$ and let $(\overrightarrow{h},\overrightarrow{h} ') \in \text {F} _i(\textbf{I},\textsf{rand})$ for some $i\in [\ell +1]$. Let $\sigma = (\textbf{c}_k, ( \textbf{r}_{k, j} )_{j \in \{ 0,1 \} })_{k \in [0:2]}$ and $\sigma ' = (\textbf{c}'_k, ( \textbf{r}'_{k, j} )_{j \in \{ 0,1 \} })_{k \in [0:2]}$ be the signatures that correspond to $\textbf{c}^{(i)}$ and $\textbf{c}'^{(i)}$, respectively, where $\textbf{c}^{(i)}$ (resp. $\textbf{c}'^{(i)}$) is the *i*-th entry of $\overrightarrow{h}$ (resp. $\overrightarrow{h} '$). In particular, we have $\textbf{c}_0 \odot \textbf{c}_1 \odot \textbf{c}_2 = \textbf{c}^{(i)}$ and $\textbf{c}'_0 \odot \textbf{c}'_1 \odot \textbf{c}'_2 = \textbf{c}'^{(i)}$. We define the witness extractors $(\textsf{Ext}_0, \textsf{Ext}_1)$ as in Fig. 7.

The following lemma establishes the correctness of the witness extractors.

##### Lemma 15

$(\textsf{Ext}_0, \textsf{Ext}_1)$ in Fig. 7 satisfy Definition 20.

##### Proof

By the definition of $\text {F} _i(\textbf{I},\textsf{rand})$ (see Definition 14), we have $(\textbf{I},\textsf{rand}, \overrightarrow{h}), (\textbf{I},\textsf{rand}, \overrightarrow{h} ') \in \textsf{Succ}$ and $\textbf{c}^{(i)} \ne \textbf{c}'^{(i)}$. The former implies that the two signatures $\sigma$ and $\sigma '$ are valid. Concretely, we have

$$\begin{aligned} \textbf{c}^{(i)} = \textbf{c}_0 \odot \textbf{c}_1 \odot \textbf{c}_2&= \textsf{H}\Big ( ( [\mathfrak {g}^{\textbf{r}_{k, j}}] *A_k^{\textbf{c}_{[k + j]_3}} )_{(k, j) \in [0:2] \times \{ 0,1 \} } \Vert \textsf{info}\Vert \textsf{M} \Big ) \\ \textbf{c}'^{(i)} = \textbf{c}_0' \odot \textbf{c}_1' \odot \textbf{c}_2'&= \textsf{H}\Big ( ( [\mathfrak {g}^{\textbf{r}'_{k, j}}] *A_k^{\textbf{c}'_{[k + j]_3}} )_{(k, j) \in [0:2] \times \{ 0,1 \} } \Vert \textsf{info}' \Vert \textsf{M}' \Big ). \end{aligned}$$

Moreover, since $\overrightarrow{h}$ and $\overrightarrow{h} '$ agree up to the *i*-th entry and the challenger and adversary’s randomness are fixed, the input to the hash functions agree. Namely, we have

$$\begin{aligned}{}[\mathfrak {g}^{\textbf{r}_{k, j}}] *A_k^{\textbf{c}_{[k + j]_3}} = [\mathfrak {g}^{\textbf{r}'_{k, j}}] *A_k^{\textbf{c}'_{[k + j]_3}} \hbox { for}\ (k, j) \in [0:2] \times \{ 0,1 \} ~\wedge ~ (\textsf{info}, \textsf{M}) = (\textsf{info}', \textsf{M}'). \end{aligned}$$

Due to the special soundness of the underlying sigma protocol (see Sect. 6.1), the witness extractors $\textsf{Ext}_0$ and $\textsf{Ext}_1$ each outputs a valid secret key from the **0**-side and **1**-side instances, respectively. Moreover, since $\textbf{c}^{(i)} \ne \textbf{c}'^{(i)}$, we must have $\textbf{c}_k \ne \textbf{c}'_k$ for some $k \in [0:2]$. Thus, at least one of $\textsf{Ext}_0$ or $\textsf{Ext}_1$ always outputs a valid secret key; if $\textbf{c}_1 \ne \textbf{c}'_1$, then they both output a valid secret key. This completes the proof. $\square$

#### Proof of one-more unforgeability

We prove the following two lemmas required to invoke the main theorem Theorem 3.

##### Lemma 16

Lemma 1 holds for the map $\Phi _{\textsf{rand}, \overrightarrow{h}}$.

##### Proof

Since the proof for the **0**-side and **1**-side instances $\textbf{I} _0$ and $\textbf{I} _1$ are analogous, we only consider the **0**-side instance. For any $\textsf{rand}, \overrightarrow{h}$, let us consider the query transcript $\overrightarrow{e} (\textbf{I} _0, \textsf{rand}, \overrightarrow{h}) = \overrightarrow{\textbf{c}^*}$, i.e., the vector of user message $\rho _U$ queries made by the adversary to the signing algorithm $\textsf{PBS}.\textsf{S}_2$. Since the underlying sigma protocol is perfectly witness indistinguishable (see Sect. 6.1), for each $i \in [\ell ]$ and $\textbf{c}^{*(i)}$, there is a set of randomness that the signer with a secret key $(1, a_1)$ (i.e., a **1**-side witness) could have used to produce the same view (i.e., first and second-signer messages) to the adversary. Concretely, this set of randomness is exactly those defined by $\Phi _{\textsf{rand}, \overrightarrow{h}}(\textbf{I} _0)$. Hence, we have $\textsf{trans} (\textbf{I} _0, \textsf{rand}, \overrightarrow{h}) = \textsf{trans} (\Phi _{\textsf{rand}, \overrightarrow{h}}(\textbf{I} _0), \textsf{rand}, \overrightarrow{h})$ as desired. Moreover, it is easy to check that $\Phi _{\textsf{rand}, \overrightarrow{h}}(\Phi _{\textsf{rand}, \overrightarrow{h}}(\textbf{I} _0))$ from the definition of $\Phi _{\textsf{rand}, \overrightarrow{h}}$. Hence, it is a bijection as desired. This completes the proof. $\square$

##### Lemma 17

Lemma 2 holds for the witness extractors $(\textsf{Ext}_0,\textsf{Ext}_1)$.

##### Proof

Since the proof of **0**-side and **1**-side witnesses are analogous, we only consider the **0**-side witness. Suppose the **0**-side witness can be extracted from base $(\textbf{I},\textsf{rand},\overrightarrow{h}),(\textbf{I},\textsf{rand},\overrightarrow{h} ')$ at index *i*, but cannot be extracted from either of the sides $(\textbf{I},\textsf{rand},\overrightarrow{h} '),(\textbf{I},\textsf{rand},\overrightarrow{h} '')$ or $(\textbf{I},\textsf{rand},\textsf{H}),(\textbf{I},\textsf{rand},\overrightarrow{h} '')$. Due to the description of our witness extractors $(\textsf{Ext}_0,\textsf{Ext}_1)$ in Fig. 7, we have $(\textbf{c}'_0, \textbf{c}'_1) = (\textbf{c}''_0, \textbf{c}''_1)$ and $(\textbf{c}_0, \textbf{c}_1) = (\textbf{c}''_0, \textbf{c}''_1)$ if the **0**-side witness cannot be extracted from either of the sides. This implies that $(\textbf{c}_0, \textbf{c}_1) = (\textbf{c}'_0, \textbf{c}'_1)$. However, this means that $\textsf{Ext}_0$ fails to extract a **0**-side witness, thus contradicting our assumption. This completes the proof. $\square$

Combining everything together, we obtain the following.

##### Theorem 18

(One-more unforgeability) The partially blind signature scheme in Fig. 6 is one-more unforgeable. More precisely, for all $\ell \in \mathbb {N}$, if there exists an adversary $\mathcal {A}$ that makes *Q* hash queries to the random oracle and breaks the $\ell$-one more unforgeability of our $\textsf{PBS}$ with advantage $$\epsilon _\mathcal {A} \ge \frac{C_1}{2^n} \cdot \left( {\begin{array}{c} Q\\ \ell + 1\end{array}}\right) $$, then there exists an algorithm $\mathcal {B}$ that breaks the ${\textsf{GAIP}}$ problem with advantage $$\epsilon _\mathcal {B}\ge C_2 \cdot \frac{\epsilon _\mathcal {A} ^2}{\left( {\begin{array}{c}Q\\ \ell + 1\end{array}}\right) ^2 \cdot (\ell + 1)^3}$$ for some universal positive constants $C_1$ and $C_2$.

##### Proof

We define the hard instance generator $\textsf{IG}$ to output a ${\textsf{GAIP}}$ instance. Then, the proof follows from the above Lemmas 1 and 2 and by invoking Theorem 3, i.e., the main theorem of Kastner, Loss, and Xu [55]. To be precise, [55, Theorem 1] is for blind signatures and not the partially blind variant—however, it can be checked that the same proof applies to our partially blind signature by observing that our definition of **0**-side and **1**-side instances are defined *independently* of the tags $\overrightarrow{\textsf{info}}$ used by the adversary, where note that $\overrightarrow{\textsf{info}}$ is implicitly defined by $(\textbf{I}, \textsf{rand}, \overrightarrow{h})$. In particular, the probability that the reduction extracts the correct witness (i.e., the witness not used by the reduction), can be bounded following the same argument as [55, Theorem 1]. $\square$

##### Remark 3

(Comparing to the Abe-Okamoto partially blind signature) We note that the reason why the same argument does not hold for the Abe-Okamoto partially blind signature [4] is that the tag $\textsf{info}$ is explicitly required to define the instances. In more detail, the Abe-Okamoto partially blind signature only has one secret key $a_0 \in \mathbb {Z}_p$ attached to the verification key $h_0 = g^{a_0} \in \mathbb {G}$. To sign with respect to a tag $\textsf{info}$, the signer hashes $\textsf{info}$ to a group element $h_\textsf{info}$ and then performs an OR proof that it knows a secret key to either $h_0$ or $h_\textsf{info}$. In the security proof, the reduction hashes $\textsf{info}$ to a group element $h_\textsf{info}= g^{a_\textsf{info}}$ while knowing the exponent $a_\textsf{info}$. In case the adversary is restricted to use only one tag $\textsf{info}$, the proof can define the **0**-side and **1**-side instances by using $a_0$ and $a_\textsf{info}$, respectively, and in particular independently of the adversary’s randomness. However, when there is more than one tag, we can no longer define a well-defined **1**-side instance. This is why Kastner, Loss and Xu and Abe and Okamoto first prove the single-tag setting and then prove the multi-tag setting by guessing which tag $\textsf{info}$ the adversary forges on.

## Optimization using higher degree roots of unity

We investigate the possibility of reducing the signature size by exploiting the $\mathbb {Z}$-module structure of the ideal class group. In this section, we present a generalized construction of the blind signature presented in Sect. 5 based on a new assumption, the *ring group action inverse problem* ($\textsf{rGAIP}$), which is a generalized version of the group action inverse problem ($\textsf{GAIP}$).

In Sects. 7.4 to 7.6, we provide the proofs of the correctness, blindness, and OMUF of the construction under the assumption that $\textsf{rGAIP}$ is hard and discuss the applicability of the partialness technique given in Sect. 6. In Sect. 8, we provide analysis on the hardness of the $\textsf{rGAIP}$ for the CSIDH-512 parameter set and show that not all $\textsf{rGAIP}$ instances are equally difficult.

### Overview and preparation

#### Notations

We summarize some notations unique to this section. We use $\mathbb {Z}_d$ to denote the set $\{ 0, \ldots , d-1 \}$. Moreover, any vector is indexed from 0, e.g., $\textbf{a}\in \mathbb {Z}_d^\kappa$ is expressed as $(a_0, \ldots , a_{\kappa - 1})$. With an overload of notations, for any integer *j*, we define the bold font $\textbf{j}$ as the length-$\kappa$ vector $(j, \dots , j)$. For any positive integer *d* and $a \in \mathbb {Z}$ or $\mathbb {Z}_d$, we use $[ a ]_d$ to denote $(a \mod d) \in \mathbb {Z}_d$. For the simplicity of the notations, we use the exponent of $\langle \zeta \rangle$ to represent the challenge space of a sigma protocol with an understanding that $\langle \zeta \rangle$ is the *d*-th primitive root of unity. That is, we will draw a challenge *c* from $\mathbb {Z}_d$. The operation between the challenges is thereby the addition $c_0+c_1$, corresponding to the multiplication of $\zeta ^{c_0+c_1} = \zeta ^{c_0}\zeta ^{c_1}$.

#### Overview

It is a natural attempt to reduce the signature size by considering a larger public key space. Indeed, as shown in [12, Sect. 5.1], such an optimization is possible for standard signature schemes by relaxing $\textsf{GAIP}$ to the multi-target $\textsf{GAIP}$. As a result, the soundness error of the underlying sigma protocol in a single round decreases to $\frac{1}{2S-1}$ from $\frac{1}{3}$ for a public key size *S*. Since the number of repetitions is decreased to $\frac{n}{\log _2 (2S-1)}$, this technique makes it possible to decrease the signature size, signing, or verification time at the cost of increased public key size. For isogeny-based protocols—which are generally slow but offer small key sizes—this is a very favorable tradeoff.

Unfortunately, a natural adaptation of the same relaxation will not apply to our case because the multi-target $\textsf{GAIP}$ does not offer the particular structure that our blind signature requires. Roughly speaking, a main component of our blind signature requires a user/verifier to compute $[\mathfrak {g}^{z + y^* d }] *E_0$ while only given $[\mathfrak {g}^{y^*}] *E_0 \in \mathcal {E}\hspace{-2.35pt}\ell \hspace{-1.30pt}\ell$, $z \in \mathbb {Z}_N$ and *d*. This is only feasible by using the quadratic twist which is when $d \in \{ -1,1 \}$. An unstructured random public key not only fails to benefit the user but also breaches the group structure of the challenge space since *d* is no longer restricted in $\{ -1, 1 \}$.

To this end, we present a novel technique that allows us to trade off between efficiency and the signature size using a structured public key. The high-level idea is fairly simple: to generalize the concept of the quadratic twist in the sense of the group action relation. In the previous section, both parties compute the action of $[\mathfrak {g}^r]$ on a curve $E_0$ or $E_0^{-1}$ with respect to the challenge $c \in \mathbb {Z}^\times _3 = \{ -1,1 \}$. Recall that $([\mathfrak {g}^r] *E_0 )^{-1}=[\mathfrak {g}^{-r}] *E_0$. In other words, the challenge $c \in \mathbb {Z}^\times _3 = \{ -1,1 \}$ is encoded into $\mathfrak {g}^{c}$. Since $-1$ is a second primitive root of unity over $\mathbb {Z}_N$, the challenge space, as a (multiplicative) group, induces an action on $\mathcal {E}\hspace{-2.35pt}\ell \hspace{-1.30pt}\ell$ by computing the twist.

We generalize the concept by expanding the challenge space to $\langle \zeta \rangle = \{ 1,\zeta ,\zeta ^2,\ldots ,\zeta ^{d-1} \}$, where $d \in \mathbb {N}$ and $\zeta$, a *d*-th primitive root of unity over $\mathbb {Z}_N^\times$; that is, $\zeta$ satisfies $\zeta ^{d}=1$ and $\zeta ^{j}\ne 1$ for any $j\in [d-1]$. Note that $\langle \zeta \rangle$ is naturally a multiplicative (sub)group, which offers the operation over the challenge space. The action $(r,c)\in \mathbb {Z}_N \times \mathbb {Z}_d$ on a curve $E_0 \in \mathcal {E}\hspace{-2.35pt}\ell \hspace{-1.30pt}\ell$ is defined to be $[\mathfrak {g}^{r \zeta ^c}]*E_0$. When $k=2$ and $\zeta$ can be taken to be $-1$, this is identical to the scheme in the previous section. However, unlike the case $d=2$ where we have the formula derived from the quadratic twist, when $d \ge 3$ the signer is required to compute $[\mathfrak {g}^{y_{b,j}^*\zeta }] *E_0$ for each $(b,j) \in [2]\times [\kappa ]$ in $\textsf{BS}.\textsf{S}_1$ to aid the user’s computation.

#### Preparation

Our construction requires one more property from the *d*-th primitive root of unity $\zeta$ to be useful.

Looking ahead, when we construct a sigma protocol for the $\textsf{rGAIP}$ relation, the special soundness extractor must solve for the secret exponent $a \in \mathbb {Z}_N$, given $c_1, c_2 \in \mathbb {Z}^2_N$ and $r_1 = y + a \zeta ^{c_1}$, $r_2 = y + a \zeta ^{c_2} \pmod {N}$ for an unknown *a* and *y*. If $\mathbb {Z}_N$ is a finite field, then this is trivial. However, in general when $\mathbb {Z}_N$ is a ring, such *a* may not be efficiently computable. One sufficient condition would be to only use a $d \in \mathbb {Z}_N$ such that $(\zeta ^{c_1} - \zeta ^{c_2})$ is invertible over $\mathbb {Z}_N$ for all distinct $(c_1, c_2) \in \mathbb {Z}_N^2$. However, this is an overly restrictive requirement and we thus make the following relaxed requirement.

##### Requirement 1

We require $$\eta _d = \textsf{lcm}_{i\in [d-1]}(\textsf{gcd}(\zeta ^i-1,N))=\textsf{poly}$$.

The requirement is equivalent to finding a *d* which divides the totient of many maximal prime power divisors of the class number (see Sect. 8.1 about the existence of, and a method for finding, such a root). Informally, when $\eta _d$ is polynomial in the security parameter *n*, then we can brute force all $a \in \mathbb {Z}_N$ such that $a \cdot (\zeta ^{c_1} - \zeta ^{c_2}) = z$ for a given $(c_1, c_2, z) \in \mathbb {Z}_N^3$. Formally, we have the following.

##### Lemma 19

Let $(N,d,\zeta )$ be a public parameter where the factorization of *N* is known and let $\eta _d = \textsf{lcm}_{i\in [d-1]}(\textsf{gcd}(\zeta ^i-1,N))$. Then, there exists an extractor $\textsf{Ext}'$ that takes as input the public parameter and $(r_1,r_2,c_1,c_2) \in \mathbb {Z}^2_N \times \mathbb {Z}_d^2$ where $c_1,c_2$ are distinct with relations $r_1 = y + a \zeta _d^{c_1}$, $r_2 = y + a \zeta _d^{c_2} \pmod {N}$, and outputs a list containing $a \in \mathbb {Z}_N$ of size not greater than $\eta _d$ in time $\textsf{poly}(\eta _d)$.

##### Proof

By calculating $(r_1-r_2)\zeta ^{-c_2}_d= a(\zeta ^{c_1-c_2}_d-1)$, the extractor solves for *a* by solving the linear equation lifted to the prime power factor of *N*, then using the Chinese remainder theorem to obtain a list of candidates of *a*. The size of the list is the number of solutions for the linear equation, which is at most $\eta _d$. $\square$

### Base sigma protocol with a large challenge space

**Fig. 8:**
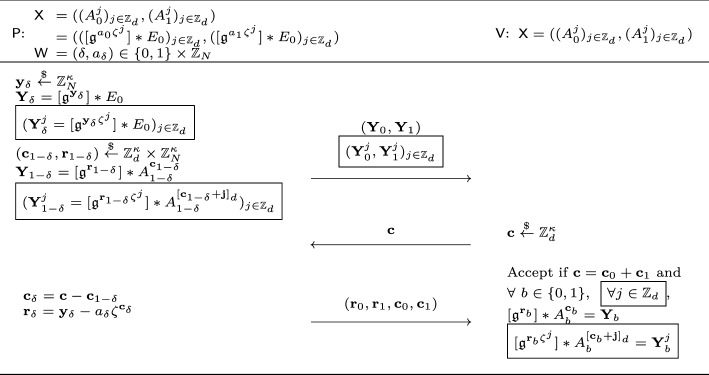
The base sigma protocol with a large challenge space, where the box is to be ignored. Recall $\mathbb {Z}_d = \{ 0, 1, \dots , d-1 \}$. $A_{b}^{j}$ denotes $[\mathfrak {g}^{a_b\zeta ^j }] *E_0$ for $j \in \mathbb {Z}_d$ and the vector $A_{b}^{[ \textbf{c} ]_d}$ denotes $(A_{b}^{[ c_0 ]_d},\ldots , A_{b}^{[ c_{\kappa -1} ]_d})$ where $\textbf{c}= (c_0,\ldots ,c_{\kappa -1}) \in \mathbb {Z}^\kappa$. If $\textbf{c}\in \mathbb {Z}^\kappa _d$, then $A_{b}^{[ \textbf{c} ]_d}$ is simply $A_{b}^{\textbf{c}}$. Other notations are explained in the paragraph above Sect. 7.2. The base sigma protocol can be made compatible with blind signatures by running the boxed lines instead of the preceding non-boxed lines

We first introduce the base sigma protocol with a larger challenge space assuming Requirement 1. This is depicted in Fig. 8 with the boxed components omitted.

We will show the correctness, HVZK, and, importantly, special soundness of this sigma protocol.

#### Correctness

It suffices to show the equation.

6 $$\begin{aligned}{}[\mathfrak {g}^{\textbf{r}_b}] *A_{b}^{\textbf{c}_b} = \textbf{Y}_{b} \end{aligned}$$

for $b \in \{ 0,1 \}$. For the case $b=1-\delta$, the equation holds naturally. For the case $b=\delta$, we have

$$\begin{aligned}{}[\mathfrak {g}^{\textbf{r}_\delta }] *A_{\delta }^{\textbf{c}_\delta }&\ = [\mathfrak {g}^{\textbf{y}_\delta - a_\delta \zeta ^{\textbf{c}_\delta }}] *A_{\delta }^{\textbf{c}_\delta } \\&\ =[\mathfrak {g}^{\textbf{y}_\delta -a_\delta \zeta ^{\textbf{c}_\delta }}] *\big ( [\mathfrak {g}^{a_\delta \zeta ^{\textbf{c}_\delta } } ] *E_0 \big ) \\&\ =\textbf{Y}_{\delta }, \end{aligned}$$

where we use the fact that $A_{\delta }^{c} = [\mathfrak {g}^{a_\delta \zeta ^c}] *E_0$ for any $c\in \mathbb {Z}_d$.

#### HVZK

Given a challenge $\textbf{c}\in \mathbb {Z}_d^\kappa$, a zero-knowledge simulator $\textsf{Sim}$ samples random $(\textbf{c}_0, \textbf{c}_1) \overset{_{ \$}}{\leftarrow } \mathbb {Z}^\kappa _d$ conditioned on $\textbf{c}_0 + \textbf{c}_1 = \textbf{c}$. Then, for each $b \in \{ 0,1 \}$, the simulator generates $\textbf{r}_{b} \overset{_{ \$}}{\leftarrow } \mathbb {Z}^\kappa _N$ and $\textbf{Y}_{b} = [\mathfrak {g}^{\textbf{r}_b}] *A_{b}^{\textbf{c}_{b}}$, and outputs $( ( \textbf{Y}_{0},\textbf{Y}_{1} ),\textbf{c}, ( \textbf{r}_0,\textbf{r}_1, \textbf{c}_0, \textbf{c}_1 ))$.

Since there is a bijection between $\textbf{r}_b$ and $\textbf{Y}_b$ once $\textbf{c}_b$ is fixed, this produces a transcript identically distributed as a real transcript.

#### Witness indistinguishability

This is a direct consequence of the above since perfect $\textsf{HVZK}$ implies perfect witness indistinguishability.

#### Special soundness

It suffices to show that special soundness holds in the case that $$\kappa =1$$. Let $$ ( ( Y_{0},Y_{1} ), c, (r_0,r_1,c_0,c_1))$$, and $$ (( Y_{0},Y_{1} ),c', (r'_0,r'_1,c'_0,c'_1)) $$ be two valid transcripts. Since $$c=c_0 + c_1$$, $$c= c'_0 + c'_1$$ and $$c \ne c'$$, we assume $$c_0 \ne c'_0$$ without loss of generality. We have $$r_0,r'_0 \in \mathbb {Z}_N$$, and distinct $$c_0,c'_0 \in \mathbb {Z}_d$$ which satisfy $$ r_0 = y + a_0 \zeta _d^{c_0}$$, $$r'_0 = y + a_0 \zeta _d^{c'_0} \pmod {N}$$ where $$y,a_0$$ are unknown. Since we assume Requirement 1 holds, we can use the extractor $$\textsf{Ext}'(r_0,r'_0,c_0,c'_0)$$ in Lemma 19 to obtain a list of size $$\eta = \textsf{lcm}_{i\in [d-1]}(\textsf{gcd}(\zeta ^i-1,N))=\textsf{poly}$$ containing $$a_0 \in \mathbb {Z}_N$$ in polynomial time. We can find $$a_0$$ from the list by running through each element in the list and checking if it maps to the statement $$( A_{0}^j )_{j\in \mathbb {Z}_d}$$ or $$( A_{1}^j )_{j\in \mathbb {Z}_d}$$. Here, we implicitly assume the statement is honestly generated and that this check always terminates.

Before explaining our blind signature, we make a subtle but important modification to our base sigma protocol depicted in Fig. 8 with the boxes. As explained in the introduction, this modification is required since the user of the blind signature is required to randomize $\textbf{Y}_b = [\mathfrak {g}^{\textbf{y}_b}] *E_0$ for $b \in \{ 0,1 \}$ to $[\mathfrak {g}^{\textbf{z}_b}] *\big ( [\mathfrak {g}^{\textbf{y}_b \zeta ^{\textbf{d}_b}}] *E_0 \big )$, where $(\textbf{z}_b, \textbf{d}_b) \overset{_{ \$}}{\leftarrow } \mathbb {Z}_N^\kappa \times \mathbb {Z}_d^\kappa$, which is no longer possible when $d \ge 3$. We will give the details of this construction in the following subsection. This extra components also play a key role when proving blindness with malicious keys.

### Enhancing the base sigma protocol for blind signatures

Before explaining our blind signature, we make a subtle but important modification to our base sigma protocol. To understand this modification, notice that if we tried to use a similar idea as in the prior sections to blind $\textbf{Y}_b = [\mathfrak {g}^{\textbf{y}_b}] *E_0$ for $b \in \{ 0,1 \}$, the user must randomize it to a value $[\mathfrak {g}^{\textbf{z}_b}] *\big ( [\mathfrak {g}^{\textbf{y}_b \zeta ^{\textbf{d}_b}}] *E_0 \big )$, where $(\textbf{z}_b, \textbf{d}_b) \overset{_{ \$}}{\leftarrow } \mathbb {Z}_N^\kappa \times \mathbb {Z}_d^\kappa$. This was doable when $d = 2$, since $\zeta = -1$ and $[\mathfrak {g}^{\textbf{y}_b \zeta ^{\textbf{d}_b}}] *E_0$ is simply the quadratic twist of $\textbf{Y}_b$. However, in general, such a computation cannot be performed. To this end, we let the prover include components that will later help the user in the blind signature. This extension to our basic sigma protocol is illustrated in Fig. 8, where the box represents the modification. The prover sends $[\mathfrak {g}^{\textbf{y}_b \zeta ^{j}}] *E_0$ for all $j \in \mathbb {Z}_d$ so that the user in the blind signature can choose whichever one based on the $\textbf{d}_d$ it samples. We also modify the verifier of the base sigma protocol to check that $[\mathfrak {g}^{\textbf{y}_b \zeta ^{\textbf{d}_b}}] *E_0$ were generated correctly. Below, we show that the extended sigma protocol satisfies correctness and HVZK. Since the extended sigma protocol includes the transcript of the base sigma protocol, special soundness is inherited.

#### Correctness

It suffices to show that

$$\begin{aligned}{}[\mathfrak {g}^{\textbf{r}_b \zeta ^j}] *A_{b}^{[ \textbf{c}_b+ \textbf{j} ]_d} = \textbf{Y}_{b}^{j} \end{aligned}$$

for any $(b,j)\in \{ 0,1 \} \times \mathbb {Z}_d$. For the case $b=1-\delta$, the equation holds by definition. For the case $b=\delta$, we have

$$\begin{aligned}{}[\mathfrak {g}^{\textbf{r}_\delta \zeta ^j }] *A_{\delta }^{[ \textbf{c}_\delta + \textbf{j} ]_d}&\ = [\mathfrak {g}^{\textbf{y}_\delta \zeta ^j - a_\delta \zeta ^{\textbf{c}_\delta + \textbf{j}}}] *A_{\delta }^{[ \textbf{c}_\delta + \textbf{j} ]_d} \\&\ =[\mathfrak {g}^{\textbf{y}_\delta \zeta ^j-a_\delta \zeta ^{\textbf{c}_\delta + \textbf{j}}}] *\big ( [\mathfrak {g}^{a_\delta \zeta ^{\textbf{c}_\delta +\textbf{j}} } ] *E_0 \big ) \\&\ =\textbf{Y}_{\delta }^{j}, \end{aligned}$$

where we use the fact that $A_{\delta }^{[ c ]_d} = [\mathfrak {g}^{a_\delta \zeta ^c}] *E_0$ for any $c\in \mathbb {Z}$.

#### HVZK

Given a challenge $\textbf{c}\in \mathbb {Z}_d^\kappa$, a zero-knowledge simulator $\textsf{Sim}$ samples random $(\textbf{c}_0, \textbf{c}_1) \overset{_{ \$}}{\leftarrow } \mathbb {Z}^\kappa _d$ conditioned on $\textbf{c}_0 + \textbf{c}_1 = \textbf{c}$. Then, for each $(b,j) \in \{ 0,1 \} \times \mathbb {Z}_d$, the simulator generates $\textbf{r}_{b} \overset{_{ \$}}{\leftarrow } \mathbb {Z}^\kappa _N$ and $\textbf{Y}^j_{b} = [\mathfrak {g}^{\textbf{r}_b\zeta ^j}] *A_{b}^{[ \textbf{c}_{b}+ \textbf{j} ]_d}$, and outputs $( ( \textbf{Y}_{0}^j,\textbf{Y}_{1}^j )_{j \in \mathbb {Z}_d},\textbf{c}, ( \textbf{r}_0,\textbf{r}_1, \textbf{c}_0, \textbf{c}_1 ))$ Since for every $j \in \mathbb {Z}_d$, there is a bijection between $\textbf{r}_b$ and $\textbf{Y}_{b}^j$ once $\textbf{c}_b$ is fixed, this produces a transcript identically distributed as a real transcript.

### Description of our optimized blind signature

**Fig. 9:**
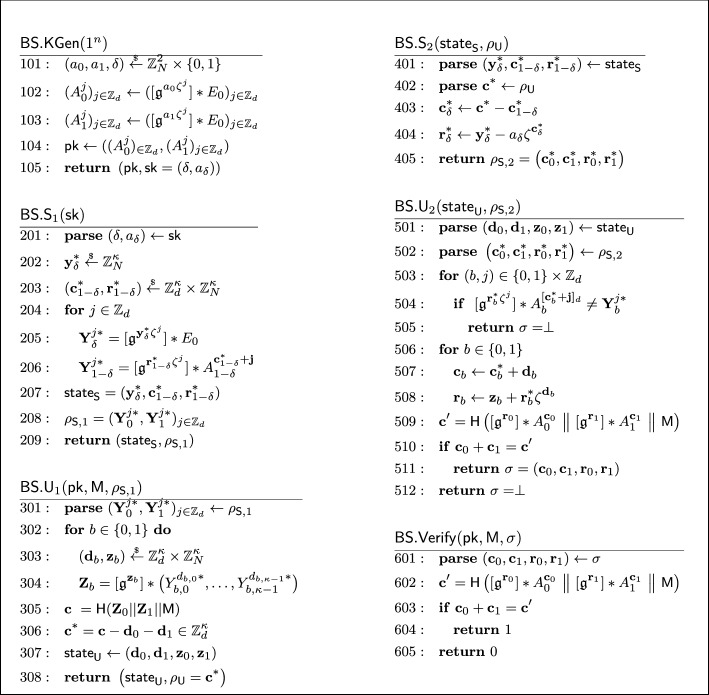
The optimized version of the blind signature where $\textsf{H}$ is a hash function and $\zeta$ is a *d*-th primitive root of unity. Recall $\mathbb {Z}_d = \{ 0, 1, \dots , d-1 \}$ and that we use the notations $\textbf{d}= (d_0, \dots , d_{\kappa -1}) \in \mathbb {Z}^\kappa _d$ and $\textbf{Y}^{j} = (Y_{0}^{j}, \dots , Y_{\kappa -1}^{j}) \in \mathcal {E}\hspace{-2.35pt}\ell \hspace{-1.30pt}\ell ^\kappa$. Moreover, if $\textbf{c}\in \mathbb {Z}^\kappa _d$, then $A_b^{[ \textbf{c} ]_d}$ is simply $A_b^\textbf{c}$ for $b \in \{ 0,1 \}$. See the caption of Fig. 8 for further explanation on the notations

We present our optimized isogeny-based blind signature building upon of the enhanced base sigma protocol in Sect. 7.2. Let $(p, N, E_0)$ be the public parameter and $\mathfrak {g}$ be a generator of the ideal class group $\mathcal {C\ell (O)}$ as in Sect. 5. Let $\zeta$ to be a *d*-th root of unity. We assume these parameters are provided to all algorithms. The parameter $\kappa \in \mathbb {N}$ indicates the number of repetition of the underlying sigma protocol such that $d^\kappa \ge 2^n$. Let $\textsf{H}: \{0,1\}^*\rightarrow \mathbb {Z}_d^\kappa$ be a hash function modeled as a random oracle. The following algorithms are summarized in Fig. 9.

$$\textsf{BS}.\textsf{KGen}\left( 1^n\right) $$:
On input the security parameter $1^n$, it samples a bit $\delta \overset{_{ \$}}{\leftarrow } \{ 0,1 \}$, $(a_0, a_1)\overset{_{ \$}}{\leftarrow } \mathbb {Z}_N^2$, and outputs a public key $\textsf{pk} = (( A_{0}^{j} )_{j\in \mathbb {Z}_d}, ( A_{1}^{j} )_{j\in \mathbb {Z}_d})$ where $A_{b}^{j} = [\mathfrak {g}^{ a_b \zeta ^j}]*E_0$ for $(b,j)\in \{ 0,1 \} \times \mathbb {Z}_d$, and secret key $\textsf{sk} = (\delta , a_\delta )$.
$$\textsf{BS}.\textsf{S}_1(\textsf{sk}):$$
The signer first samples $\textbf{y}_{\delta }^* \overset{_{ \$}}{\leftarrow } \mathbb {Z}^\kappa _N$ and sets $\textbf{Y}_{\delta }^{j*} = [\mathfrak {g}^{\textbf{y}_\delta ^*\zeta ^j}] *E_0$ for $j \in \mathbb {Z}_d$. It then samples $(\textbf{c}^*_{1 -\delta }, \textbf{r}^*_{1 - \delta }) \overset{_{ \$}}{\leftarrow } \mathbb {Z}^{\kappa }_d \times \mathbb {Z}_N^\kappa$ and sets $\textbf{Y}_{1 - \delta }^{j*} = [\mathfrak {g}^{\textbf{r}^*_{1 - \delta } \zeta ^j }] *A_{ 1-\delta }^{\textbf{c}^*_{1 - \delta } + \textbf{j}}$ for $j \in \mathbb {Z}_d$. It then outputs the signer state $\textsf{state}_\textsf{S}= (\textbf{y}_\delta ^*, \textbf{c}^*_{1 -\delta }, \textbf{r}^*_{1 - \delta })$ and the first-sender message $\rho _{\textsf{S}, 1}= ( \textbf{Y}_{0}^{j*}, \textbf{Y}_{1}^{j*} )_{j\in \mathbb {Z}_d}$.
$$\textsf{BS}.\textsf{U}_1(\textsf{pk}, \textsf{M}, \rho _{\textsf{S}, 1}):$$
The user parses $( \textbf{Y}_{0}^{j*}, \textbf{Y}_{1}^{j*} )_{j\in \mathbb {Z}_d} \leftarrow \rho _{\textsf{S}, 1}$, samples $(\textbf{d}_{b}, \textbf{z}_{b}) \overset{_{ \$}}{\leftarrow } \mathbb {Z}^\kappa _d \times \mathbb {Z}^\kappa _N$, and computes $\textbf{Z}_{b} = [\mathfrak {g}^{ \textbf{z}_{b}}] *\big ( Y_{b, 0}^{d_{b, 0}*}, \dots , Y_{b, \kappa -1}^{d_{b, \kappa -1}*} \big )$ for $b \in \{ 0,1 \}$. Here, note that $Y_{b, j}^{d_{b, j}*}$ denotes the *j*-th ($j \in \mathbb {Z}_d$) element of $\textbf{Y}_b^{d_{b, j}*} \in \mathcal {E}\hspace{-2.35pt}\ell \hspace{-1.30pt}\ell ^\kappa$ and $d_{b, j}$ is the *j*-th element of $\textbf{d}_b \in \mathbb {Z}_d^\kappa$. It then computes $\textbf{c}= \textsf{H}( \textbf{Z}_0 \Vert \textbf{Z}_1 \Vert \textsf{M}) \in \mathbb {Z}_d^\kappa$ and outputs the user state $\textsf{state}_\textsf{U}= (\textbf{d}_0, \textbf{d}_1, \textbf{z}_0, \textbf{z}_1)$ and user message $\rho _\textsf{U}= \textbf{c}^* = \textbf{c}- \textbf{d}_0 - \textbf{d}_1$.
$$\textsf{BS}.\textsf{S}_2(\textsf{state}_\textsf{S}, \rho _\textsf{U}):$$
The signer parses $(\textbf{y}_\delta ^*, \textbf{c}^*_{1 -\delta }, \textbf{r}^*_{1 - \delta }) \leftarrow \textsf{state}_\textsf{S}$, $\textbf{c}^* \leftarrow \rho _\textsf{U}$, sets $\textbf{c}_{\delta }^* = \textbf{c}^* + \textbf{c}_{1-\delta }^* \in \mathbb {Z}_d^\kappa$, and computes $\textbf{r}_\delta ^* = \textbf{y}_\delta ^* - a_\delta \zeta ^{\textbf{c}_\delta ^*} \in \mathbb {Z}_N^\kappa$. It then outputs the second-signer message $\rho _{\textsf{S}, 2}= (\textbf{c}^*_0, \textbf{c}^*_1, \textbf{r}^*_0,\textbf{r}^*_1)$.
$$\textsf{BS}.\textsf{U}_2(\textsf{state}_\textsf{U}, \rho _{\textsf{S}, 2}):$$
The user parses $(\textbf{d}_0, \textbf{d}_1, \textbf{z}_0, \textbf{z}_1) \leftarrow \textsf{state}_\textsf{U}$, $(\textbf{c}^*_0, \textbf{c}^*_1, \textbf{r}^*_0,\textbf{r}^*_1) \leftarrow \rho _{\textsf{S}, 2}$ and checks if $[\mathfrak {g}^{\textbf{r}^*_{b}\zeta ^j}] *A_{b}^{[ \textbf{c}^*_{b}+\textbf{j} ]_d} = \textbf{Y}_{b}^{j^*}$ holds for all $(b, j) \in \{ 0,1 \} \times \mathbb {Z}_d$. If not, it outputs $\bot$. Otherwise, it sets $(\textbf{c}_b, \textbf{r}_b) = (\textbf{c}^*_b + \textbf{d}_b, \textbf{z}_b + \textbf{r}_b^* \zeta ^{\textbf{d}_b}) \in \mathbb {Z}_d^\kappa \times \mathbb {Z}_N^\kappa$ for $b \in \{ 0,1 \}$. It then checks if
  7 $$\begin{aligned} \textbf{c}_0 + \textbf{c}_1 = \textsf{H}\Big ( [\mathfrak {g}^{\textbf{r}_0}] *A_{0}^{\textbf{c}_0} \Vert [\mathfrak {g}^{\textbf{r}_1}] *A_{1}^{\textbf{c}_1} \Vert \textsf{M} \Big ). \end{aligned}$$
  If it holds, it outputs a signature $\sigma = (\textbf{c}_0,\textbf{c}_1,\textbf{r}_0,\textbf{r}_1) \in ( \mathbb {Z}_d^\kappa )^2 \times ( \mathbb {Z}_N^\kappa )^2$, and otherwise $\bot$.
$$\textsf{BS}.\textsf{Verify}(\textsf{pk}, \textsf{M}, \sigma )$$:
The verifier outputs 1 if Eq. 7 holds, and otherwise 0.

#### Remark 4

One can observe that the only source of overhead in the communication bandwidth compared to the blind signature in Sect. 5 is in $\textsf{BS}.\textsf{S}_1$. The bandwidth is increased by a factor of $\frac{d\kappa }{2n}$.

#### Remark 5

We remark that it is possible to fuse our partial blindness technique and the generalized construction in this section and obtain an optimized PBS variant. By doing so, we can obtain a PBS with a smaller signature size based on the $\textsf{rGAIP}$. Roughly, there are three sequences of the curves in the public statement $(A_0, A_1, A_2) = \left( ([\mathfrak {g}^{a_0 \zeta _j }]*E_0)_{j\in \mathbb {Z}_d}, ([\mathfrak {g}^{ a_1\zeta _j}]*E_0)_{j\in \mathbb {Z}_d}, ([\mathfrak {g}^{a_2\zeta _j }]*E_0)_{j\in \mathbb {Z}_d}\right)$ where the secret key of the third public key is derived from the public information. The underlying sigma protocol is to prove for a two-out-of-three secret corresponding to this statement.

However, given the proofs in Sect. 6 and in this section, we expect the proof to be highly involved. We leave this as a future work.

### Proof of correctness and blindness

The subsection shows that our blind signature presented in Sect. 7.4 has (perfect) correctness and blindness.

#### Theorem 20

The blind signature scheme in Fig. 9 is (perfectly) correct.

#### Proof

To show correctness, it suffices to show the equation

$$\begin{aligned} \textbf{c}_0 + \textbf{c}_1 = \textsf{H}\left( [\mathfrak {g}^{\textbf{r}_0}] *A_{0}^{\textbf{c}_0} \ \parallel \ [\mathfrak {g}^{\textbf{r}_1}] *A_{1}^{\textbf{c}_1}\ \parallel \ \textsf{M}\right) \end{aligned}$$

holds when both the signer and user follow the protocol.

From the description of $\textsf{BS}.\textsf{U}_1, \textsf{BS}.\textsf{S}_2$ and $\textsf{BS}.\textsf{U}_2$, we have $\textbf{c}= \textbf{c}^* + \textbf{d}_0 + \textbf{d}_1$, $\textbf{c}^* = \textbf{c}_1^* + \textbf{c}_2^*$, and $\textbf{c}_b = \textbf{c}_b^* + \textbf{d}_b$ for $b \in \{ 0,1 \}$. Therefore, we have $\textbf{c}= \textbf{c}_0 + \textbf{c}_1$, which shows the l.h.r. equation. It remains to show $\textbf{Z}_b = [\mathfrak {g}^{\textbf{r}_b}] *A_{b}^{\textbf{c}_b}$ for each $b \in \{ 0,1 \}$. Following the definition of $\textbf{Z}_b$ computed by $\textsf{BS}.\textsf{U}_1$, we have

8 $$\begin{aligned} \textbf{Z}_b&= [\mathfrak {g}^{ \textbf{z}_{b}}] *\big ( Y_{b, 0}^{d_{b, 0}*}, \dots , Y_{b, \kappa -1}^{d_{b, \kappa -1}*} \big ) \nonumber \\&= [\mathfrak {g}^{ \textbf{z}_{b}}] *\big ( [\mathfrak {g}^{r^*_{b, 0}\zeta ^{d_{b, 0}}}] *A_{b}^{[ c^*_{b, 0}+ {d_{b, 0}} ]_d}, \dots , [\mathfrak {g}^{r^*_{b, \kappa -1}\zeta ^{d_{b, \kappa -1}}}] *A_{b}^{[ c^*_{b, \kappa -1}+ {d_{b, \kappa -1}} ]_d} \big ) \end{aligned}$$

9 $$\begin{aligned}&= \big ( [\mathfrak {g}^{z_{b, 0} + r^*_{b, 0}\zeta ^{d_{b, 0}}}] *A_{b}^{[ c^*_{b, 0}+ {d_{b, 0}} ]_d}, \dots , [\mathfrak {g}^{z_{b, \kappa -1} + r^*_{b, \kappa -1}\zeta ^{d_{b, \kappa -1}}}] *A_{b}^{[ c^*_{b, \kappa -1}+ {d_{b, \kappa -1}} ]_d} \big ) \nonumber \\&= [\mathfrak {g}^{\textbf{r}_b}] *A_{b}^{\textbf{c}_b}, \end{aligned}$$

where Eq. 8 follows from the check performed by $\textsf{BS}.\textsf{U}_2$ and Eq. 9 follows from the definition of $(\textbf{c}_b, \textbf{r}_b)$. $\square$

Next, we will show the generalized blind signature has perfect blindness. Notably, blindness holds even under adversarially chosen keys. This is a strong property since if a malicious signer uses malformed supersingular curves in $\mathcal {E}\hspace{-2.35pt}\ell \hspace{-1.30pt}\ell$ without the ring structure as the public key, the user cannot detect this. The main reason why we can argue perfect blindness is that if the public key is malformed, then the pair of curves in the first message $( \textbf{Y}_{0}^{j*},\textbf{Y}_{1}^{j*} )_{j \in \mathbb {Z}_d}$ is also malformed in a controlled manner. If there exists one user state that leads to a valid signature, then we can argue that the first message must be in a specific (but possibly incorrect) form regardless of the user state. Using this, we are able to establish a bijection between an arbitrary user state and a valid signature conditioning on a fixed first and second signature messages and a user message. Namely, any valid signature could have been produced with an equal probability.

#### Theorem 21

The blind signature scheme in Fig. 9 is (perfectly) blind under chosen keys.

#### Proof

Let $(\rho _{{\textsf{S}, 1}, 0}, \rho _{{\textsf{S}, 2}, 0})$ and $(\rho _{{\textsf{S}, 1}, 1}, \rho _{{\textsf{S}, 2}, 1})$ be the two sets of first and second-signer message pairs the adversary $\mathcal {A}$ queries to oracles $\textsf{U}_1$ and $\textsf{U}_2$. Moreover, let $\rho _{\textsf{U}, b}$ be the user message returned by oracle $\textsf{U}_1$ when $\mathcal {A}$ queries with $\rho _{{\textsf{S}, 1}, b}$ for $b \in \{ 0,1 \}$, and let $(\sigma _\textsf{coin}, \sigma _{1 - \textsf{coin}})$ be the two signatures $\mathcal {A}$ sees at the end, where note that these two corresponds to $\widetilde{\textsf{M}}_0$ and $\widetilde{\textsf{M}}_1$, respectively, regardless of the choice of $\textsf{coin}\in \{ 0,1 \}$. We call $(\rho _{{\textsf{S}, 1}, b}, \rho _{\textsf{U}, b}, \rho _{{\textsf{S}, 2}, b})_{b \in \{ 0,1 \} }$ the *view* of $\mathcal {A}$. To prove perfect blindness, it suffices to prove that the view is independent of $\textsf{coin}\in \{ 0,1 \}$. In other words, since the randomness used by oracle $\textsf{U}_1$ is defined by $( \textsf{state}_{\textsf{U}, b} )_{b \in \{ 0,1 \} }$ and oracle $\textsf{U}_2$ is deterministic, we prove that there exist two sets of states $( \textsf{state}^{(0)}_{\textsf{U}, b} )_{b \in \{ 0,1 \} }$ and $( \textsf{state}^{(1)}_{\textsf{U}, b} )_{b \in \{ 0,1 \} }$ that can be sampled by oracle $\textsf{U}_1$ with an equal probability such that they generate the same view to $\mathcal {A}$ but produce a different pair of signatures $(\sigma _0, \sigma _1)$ and $(\sigma _1, \sigma _0)$, respectively. Considering that the set of valid signature space and user randomness/state space is identical, we prove a stronger statement that for any non-aborting (partial) view $(\rho _{{\textsf{S}, 1}, 0}, \rho _{\textsf{U}, 0}, \rho _{{\textsf{S}, 2}, 0})$ of $\mathcal {A}$, there is a bijection between a valid signature $\sigma _0$ on message $\textsf{M}_0$ and a state $\textsf{state}_{\textsf{U}, 0}$ of the oracle $\textsf{U}_1$. Below, we drop the subscript 0 for readability.

Let us denote the first and second-signer message as $\rho _{{\textsf{S}, 1}} = ( \textbf{Y}_{0}^{j*}, \textbf{Y}_{1}^{j*} )_{j\in \mathbb {Z}_d}$, $\rho _{{\textsf{S}, 2}} = (\textbf{c}^*_0, \textbf{c}^*_1, \textbf{r}^*_0,\textbf{r}^*_1)$, a user message as $\rho _{\textsf{U}} = \textbf{c}^*$, and a valid signature for message $\textsf{M}$ as $\sigma = (\textbf{c}_0,\textbf{c}_1,\textbf{r}_0,\textbf{r}_1) \in ( \mathbb {Z}^\kappa _d )^2 \times ( \mathbb {Z}_N^\kappa )^2$. Here, note that any public key $\textsf{pk} = (( A_{0}^{j} )_{j\in \mathbb {Z}_d}, ( A_{1}^{j} )_{j\in \mathbb {Z}_d})$ output by the adversary (i.e., malicious signer) $\mathcal {A}$ can be efficiently checked to be valid elliptic curves (i.e., supersingularity) but cannot be checked if it has the correct cyclic structure.

We define a map between the signature $\sigma = (\textbf{c}_0,\textbf{c}_1,\textbf{r}_0,\textbf{r}_1)$ and user state $\textsf{state}_\textsf{U}= (\textbf{d}_0, \textbf{d}_1, \textbf{z}_0, \textbf{z}_1)$ by $\textbf{d}_b = \textbf{c}_b - \textbf{c}^*_b$ and $\textbf{z}_b = \textbf{r}_b - \textbf{r}^*_b \cdot \zeta ^{\textbf{d}_b}$ for $b \in \{ 0,1 \}$. It is easy to check that once the view (or $\rho _{{\textsf{S}, 2}} = (\textbf{c}^*_0, \textbf{c}^*_1, \textbf{r}^*_0,\textbf{r}^*_1)$) is fixed, then this mapping is indeed a bijection. It remains to prove that this $\textsf{state}_\textsf{U}$ is a state that produces $\sigma$.

Observe that if $\textsf{BS}.\textsf{U}_1(\textsf{pk}, \textsf{M}, \rho _{\textsf{S}, 1})$ samples $\textsf{state}_\textsf{U}$, then it computes $\textbf{Z}_b = [\mathfrak {g}^{ \textbf{z}_{b}}] *\big ( Y_{b, 0}^{d_{b, 0}^*}, \dots , Y_{b, \kappa -1}^{d_{b, \kappa -1}^*} \big )$ for $b \in \{ 0,1 \}$ using $\rho _{\textsf{S}, 1}$. It then sets $\textbf{c}' = \textsf{H}(\textbf{Z}_0 || \textbf{Z}_1 || \textsf{M})$ and defines $\rho '_\textsf{U}= \textbf{c}'^{*} = \textbf{c}' - \textbf{d}_0 - \textbf{d}_1$. Moreover, due to restrictions on the blindness game, the view is non-aborting for at least one state $\textsf{state}_\textsf{U}$. Combining this with the fact that the first check performed by $\textsf{BS}.\textsf{U}_2(\textsf{state}_\textsf{U}, \rho _{\textsf{S}, 2})$ only depends on $\rho _{\textsf{S}, 2}$, and in particular independent of $\textsf{state}_\textsf{U}$, we have $[\mathfrak {g}^{\textbf{r}^*_{b}\zeta ^j}] *A_{b}^{[ \textbf{c}^*_{b}+\textbf{j} ]_d} = \textbf{Y}_{b}^{j^*}$ for $j \in \mathbb {Z}_d$ and any state $\textsf{state}_\textsf{U}$. Therefore, $\textsf{BS}.\textsf{U}_2$ always outputs $\sigma$ as desired since the signature $\sigma$ is assumed to be valid.

It remains to check that $\rho '_\textsf{U}= \textbf{c}'^{*}$ generated by $\textsf{BS}.\textsf{U}_1$ is the desired $\rho _\textsf{U}= \textbf{c}^*$ to complete the proof. Since $\sigma$ is valid and due to the definition of $\textsf{state}_\textsf{U}$, we have $\textbf{c}^*_0 + \textbf{c}_1^* + \textbf{d}_0 + \textbf{d}_1 = \textsf{H}\left( [\mathfrak {g}^{\textbf{r}_0}] *A_{0}^{\textbf{c}_0} \ \parallel \ [\mathfrak {g}^{\textbf{r}_1}] *A_{1}^{\textbf{c}_1} \ \parallel \ \textsf{M}\right)$. Moreover, since the view is non-aborting, we are guaranteed that $\textbf{c}^* = \textbf{c}^*_0 + \textbf{c}^*_1$. Therefore, if $\textbf{Z}_b = [\mathfrak {g}^{\textbf{r}_b}] *A_{b}^{\textbf{c}_b}$ for $b \in \{ 0,1 \}$, then we can conclude that $\textbf{c}^* = \textbf{c}'^{*}$ as desired. This can be checked as follows, where we use the fact that $[\mathfrak {g}^{\textbf{r}^*_{b}\zeta ^j}] *A_{b}^{[ \textbf{c}^*_{b}+\textbf{j} ]_d} = \textbf{Y}_{b}^{j^*}$ for $j \in \mathbb {Z}_d$ in the second equality:

$$\begin{aligned} \textbf{Z}_b&= [\mathfrak {g}^{ \textbf{z}_{b}}] *\big ( Y_{b, 0}^{d_{b, 0}*}, \dots , Y_{b, \kappa -1}^{d_{b, \kappa -1}*} \big )\\&= [\mathfrak {g}^{ \textbf{z}_{b}}] *\big ( [\mathfrak {g}^{r^*_{b, 0}\zeta ^{d_{b, 0}}}] *A_{b}^{[ c^*_{b, 0}+ d_{b, 0} ]_d}, \dots , [\mathfrak {g}^{r^*_{b, \kappa -1}\zeta ^{d_{b, \kappa -1}}}] *A_{b}^{[ c^*_{b, \kappa -1}+ d_{b,\kappa -1} ]_d} \big )\\&= \big ( [\mathfrak {g}^{z_{b, 0} + r^*_{b, 0}\zeta ^{d_{b, 0}}}] *A_{b}^{[ c^*_{b, 0}+ d_{b, 0} ]_d}, \dots , [\mathfrak {g}^{z_{b, \kappa - 1} + r^*_{b, \kappa -1}\zeta ^{d_{b, \kappa -1}}}] *A_{b}^{[ c^*_{b, \kappa -1}+ d_{b,\kappa -1} ]_d} \big ) \\&= [\mathfrak {g}^{\textbf{r}_b}] *A_{b}^{\textbf{c}_b}. \end{aligned}$$

This completes the proof. $\square$

### Proof of one-more unforgeability

Our proof of OMUF consists of preparing the necessary tools present in Sect. 4 to invoke Theorem 3. Specifically, we define instances $\textbf{I} _0,\textbf{I} _1$ (see Definition 12), the map $\Phi _{\textsf{rand}, \overrightarrow{h}}$ (see Definition 19), the witness extractors $(\textsf{Ext}_0, \textsf{Ext}_1)$ (see Definition 20) and prove that Lemmas 1 and 2 hold. We refer the readers to Sect. 5.5 for some of the notations used below.

*Preparation: instances*

Let us first define the **0**-side instance $\textbf{I} _0$ and the **1**-side instance $\textbf{I} _1$. Below, we assume the adversary against the one-more unforgeability game makes $\ell$-signing queries in total.

A **0**-side instance $\textbf{I} _0=( 0,a_0,\textbf{A}_1, \overrightarrow{\textbf{y}^*_0},\overrightarrow{\textbf{r}^*_1},\overrightarrow{\textbf{c}^*_1} )$ is defined as follows:

- $(0,a_0):$ The secret key $\textsf{sk}$ when $\delta =0$.
- $\textbf{A}_1:$ The part of the public key $\textsf{pk} = ( \textbf{A}_0 = ( A_{0}^{j} )_{j\in \mathbb {Z}_d}, \textbf{A}_1 = ( A_{1}^{j} )_{j\in \mathbb {Z}_d})$ whose secret key is unknown.
- $\textbf{y}^{*(k)}_0:$ The exponent of the commitment $( \textbf{Y}_{0}^{j*(k)} )_{j\in \mathbb {Z}_d}$ in the *k*-th ($k\in [\ell ]$) first-sender message when $\delta =0$ such that $\textbf{Y}_{0}^{j*(k)}= [\mathfrak {g}^{\textbf{y}^{*(k)}_{0} \zeta ^j }] *E_0$ for each $j \in \mathbb {Z}_d$.
- $\textbf{c}^{*(k)}_{1}:$ The simulated challenge in the *k*-th ($k \in [\ell ]$) first-sender message when $\delta = 0$.
- $\textbf{r}^{*(k)}_{1}:$ The exponent of the commitment $\textbf{Y}_{1}^{j*(k)}$ in the *k*-th ($k \in [\ell ]$) first-sender message when $\delta = 0$ such that $\textbf{Y}_{1}^{j*(k)}= [\mathfrak {g}^{\textbf{r}^{*(k)}_{1}\zeta ^j}] *A_{1}^{[ \textbf{c}^{*(k)}_1+ \textbf{j} ]_d}$ for each $j \in \mathbb {Z}_d$.

A **1**-side instance $\textbf{I} _1=( 1,a_1,\textbf{A}_0,\overrightarrow{\textbf{y}^*_1},\overrightarrow{\textbf{r}^*_0},\overrightarrow{\textbf{c}^*_0} )$ is defined as follows:

- $(1,a_1):$ The secret key $\textsf{sk}$ when $\delta =1$.
- $\textbf{A}_0:$ The part of the public key $\textsf{pk} = ( \textbf{A}_0 = ( A_{0}^{j} )_{j\in \mathbb {Z}_d}, \textbf{A}_1 = ( A_{1}^{j} )_{j\in \mathbb {Z}_d})$ whose secret key is unknown.
- $\textbf{y}^{*(k)}_1:$ The exponent of the commitment $( \textbf{Y}_{1}^{j*(k)} )_{j\in \mathbb {Z}_d}$ in the *k*-th ($k\in [\ell ]$) first-sender message when $\delta =1$ such that $( \textbf{Y}_{1}^{j*(k)} )_{j\in \mathbb {Z}_d} = [\mathfrak {g}^{\textbf{y}^{*(k)}_{1} \zeta ^j }] *E_0$ for each $j \in \mathbb {Z}_d$.
- $\textbf{c}^{*(k)}_{0}:$ The simulated challenge in the *k*-th ($k \in [\ell ]$) first-sender message when $\delta = 1$.
- $\textbf{r}^{*(k)}_{0}:$ The exponent of the commitment $\textbf{Y}_{0}^{j*(k)}$ in the *k*-th ($k \in [\ell ]$) first-sender message when $\delta = 1$ such that $\textbf{Y}_{0}^{j*(k)}= [\mathfrak {g}^{\textbf{r}^{*(k)}_{0}\zeta ^j}] *A_{0}^{[ \textbf{c}^{*(k)}_0+ \textbf{j} ]_d}$ for each $j \in \mathbb {Z}_d$.

#### Preparation: Map $\Phi _{\textsf{rand}, \overrightarrow{h}}$

We next define the map $\Phi _{\textsf{rand}, \overrightarrow{h}}$ that maps a **0**-side instance $\textbf{I} _0$ into a **1**-side instance $\textbf{I} _1$ and vice versa. Concretely, a **0**-side instance $\textbf{I} _0 =( 0,a_0,\textbf{A}_1, \overrightarrow{\textbf{y}^*_0},\overrightarrow{\textbf{r}^*_1},\overrightarrow{\textbf{c}^*_1} )$ maps to a **1**-side instance $\textbf{I} _1$ such that

$$\begin{aligned} \textbf{I} _1&= ( 1,a_1, \ A_0= ( A_{0}^{j} )_{j\in \mathbb {Z}_d} = ( [\mathfrak {g}^{a_0\zeta ^j }]*E_0 )_{j \in \mathbb {Z}_d}, \ \overrightarrow{\textbf{y}_1^*}= \overrightarrow{\textbf{r}_1^*} + a_1\zeta ^{\overrightarrow{\textbf{c}_1^*}} , \ \overrightarrow{\textbf{c}_0^*} = \overrightarrow{\textbf{c}^*} - \overrightarrow{\textbf{c}_1^*}, \ \overrightarrow{\textbf{r}_0^*}\\ {}&=\overrightarrow{\textbf{y}_0^*} -a_0 \zeta ^{\overrightarrow{\textbf{c}_0^*}} ), \end{aligned}$$

where $a_1 \in \mathbb {Z}_N$ such that $A_1^0 = [\mathfrak {g}^{a_1}] *E_0$ and recall that $\overrightarrow{\textbf{c}^*} = \overrightarrow{e} (\textbf{I} _0,\textsf{rand},\overrightarrow{h})$. On the other hand, a **1**-side instance $\textbf{I} _1 =( 1,a_1,\textbf{A}_0,\overrightarrow{\textbf{y}^*_1},\overrightarrow{\textbf{r}^*_0},\overrightarrow{\textbf{c}^*_0} )$ maps to a **0**-side instance $\textbf{I} _0$ such that

$$\begin{aligned} \textbf{I} _0&= ( 0,a_0, \ \textbf{A}_1 = ( A_{1}^{j} )_{j\in \mathbb {Z}_d} = ( [\mathfrak {g}^{a_1\zeta ^j }]*E_0 )_{j \in \mathbb {Z}_d}, \ \overrightarrow{\textbf{y}_0^*} = \overrightarrow{\textbf{r}_0^*} + a_0\zeta ^{\overrightarrow{\textbf{c}_0^*}} , \ \overrightarrow{\textbf{c}_1^*} = \overrightarrow{\textbf{c}^*} - \overrightarrow{\textbf{c}_0^*}, \ \overrightarrow{\textbf{r}_1^*} \\ {}&= \overrightarrow{\textbf{y}_1^*} - a_1\zeta ^{\overrightarrow{\textbf{c}_1^*}} ) \end{aligned}$$

where $a_0 \in \mathbb {Z}_N$ such that $A_0^0 = [\mathfrak {g}^{a_0}] *E_0$ and recall that $\overrightarrow{\textbf{c}^*} = \overrightarrow{e} (\textbf{I} _1,\textsf{rand},\overrightarrow{h})$.

#### Preparation: witness extractors $(\textsf{Ext}_0, \textsf{Ext}_1)$

**Fig. 10:**
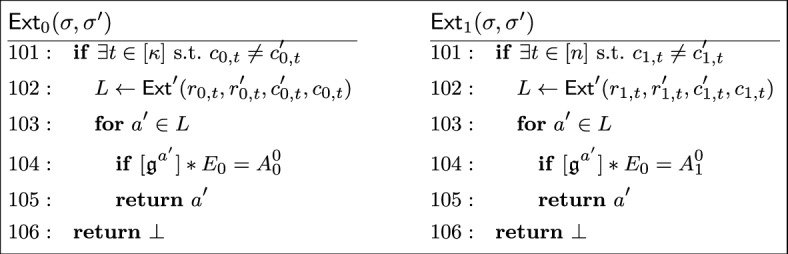
Witness extractors for our generalized blind signature for $\sigma , \sigma '$. In the above, $\sigma =(\textbf{c}_{0},\textbf{c}_{1},\textbf{r}_{0},\textbf{r}_{1})$ and $\sigma ^\prime =(\textbf{c}'_{0},\textbf{c}_{1}',\textbf{r}'_{0},\textbf{r}'_{1})$, where $\textbf{c}_0, \textbf{c}_1,\textbf{c}'_0,\textbf{c}'_1$ live in $\mathbb {Z}_d^\kappa$ and $\textbf{r}_0, \textbf{r}_1,\textbf{r}_0', \textbf{r}_1'$ live in $\mathbb {Z}^\kappa _N$. $\textsf{Ext}'$ is the extractor in Lemma 19. Non-bold font indicates the entries of a vector

Fix $\textbf{I},\textsf{rand}$ and let $(\overrightarrow{h},\overrightarrow{h}^{\prime })\in F_i(\textbf{I},\textsf{rand})$ for some $i\in [\ell +1]$. Moreover, denote the two signatures $\sigma =(\textbf{c}_{0},\textbf{c}_{1},\textbf{r}_{0},\textbf{r}_{1}),\sigma '=(\textbf{c}'_{0},\textbf{c}_{1}',\textbf{r}'_{0},\textbf{r}'_{1})$ be the signatures that correspond to $\textbf{c}^{(i)}$ and $\textbf{c}'^{(i)}$, respectively, where recall $\textbf{c}^{(i)}$ (resp. $\textbf{c}'^{(i)}$) is the *i*-th entry of $\overrightarrow{h}$ (resp. $\overrightarrow{h} '$). In particular, we have $\textbf{c}_0 + \textbf{c}_1 = \textbf{c}^{(i)}$ and $\textbf{c}'_0 + \textbf{c}'_1= \textbf{c}'^{(i)}$. We define the witness extractors $(\textsf{Ext}_0, \textsf{Ext}_1)$ as in Fig. 10.

The following lemma establishes the correctness of the witness extractors.

##### Lemma 22

$(\textsf{Ext}_0, \textsf{Ext}_1)$ in Fig. 10 satisfy the definition of witness extractors in Definition 20.

##### Proof

By the definition of $\text {F} _i(\textbf{I},\textsf{rand})$ (see Definition 14), we have $(\textbf{I},\textsf{rand}, \overrightarrow{h}), (\textbf{I},\textsf{rand}, \overrightarrow{h} ') \in \textsf{Succ}$ and $\textbf{c}^{(i)} \ne \textbf{c}'^{(i)}$. The former implies that the two signatures $\sigma$ and $\sigma '$ are valid. Concretely, we have

$$\begin{aligned} \textbf{c}^{(i)} = \textbf{c}_0 + \textbf{c}_1&= \textsf{H}\Big ( [\mathfrak {g}^{\textbf{r}_0}] *A_{0}^{\textbf{c}_0} \ \Vert \ [\mathfrak {g}^{\textbf{r}_1}] *A_{1}^{\textbf{c}_1} \ \Vert \ \textsf{M} \Big )\\ \textbf{c}'^{(i)} = \textbf{c}_0' + \textbf{c}_1'&= \textsf{H}\Big ( [\mathfrak {g}^{\textbf{r}'_0}] *A_{0}^{\textbf{c}'_0} \ \Vert \ [\mathfrak {g}^{\textbf{r}'_1}] *A_{1}^{\textbf{c}'_1} \ \Vert \ \textsf{M}' \Big ). \end{aligned}$$

Moreover, since $\overrightarrow{h}$ and $\overrightarrow{h} '$ agree up to the *i*-th entry and the challenger and adversary’s randomness are fixed, the input to the hash functions agree. Namely, we have

$$\begin{aligned}{}[\mathfrak {g}^{\textbf{r}_0}] *A_{0}^{\textbf{c}_0} = [\mathfrak {g}^{\textbf{r}'_0}] *A_{0}^{\textbf{c}'_0} ~~\wedge ~~ [\mathfrak {g}^{\textbf{r}_1}] *A_{1}^{\textbf{c}_1} = [\mathfrak {g}^{\textbf{r}'_1}] *A_{1}^{\textbf{c}'_1} ~~\wedge ~~ \textsf{M}= \textsf{M}' \end{aligned}$$

Since $\textbf{c}^{(i)} \ne \textbf{c}'^{(i)}$, we must have $\textbf{c}_0 \ne \textbf{c}'_0$ or $\textbf{c}_1\ne \textbf{c}'_1$. Thus, due to the special soundness of the underlying sigma protocol (see Sect. 7.2) one of $\textsf{Ext}_0$ or $\textsf{Ext}_1$ always outputs a valid secret key. This completes the proof. $\square$

#### Proof of one-more unforgeability

We have the following two lemmas required to invoke the main theorem Theorem 25. Since the proof is almost identical to our earlier proofs in Sect. 5.5, we omit the proof of the lemmas.

##### Lemma 23

Lemma 1 holds for our definition of the map $\Phi _{\textsf{rand}, \overrightarrow{h}}$ above.

##### Lemma 24

Lemma 2 holds for our definition of the witness extractors $(\textsf{Ext}_0,\textsf{Ext}_1)$ Fig. 10.

Combining everything together, we obtain the following.

##### Theorem 25

(One-more unforgeability) The partially blind signature scheme in Fig. 9 is one-more unforgeable. More precisely, for all $\ell \in \mathbb {N}$, if there exists an adversary $\mathcal {A}$ that makes *Q* hash queries to the random oracle and breaks the $\ell$-one more unforgeability of our scheme with advantage $$\epsilon _\mathcal {A} \ge \frac{C_1}{d^\kappa } \cdot \left( {\begin{array}{c} Q\\ \ell + 1\end{array}}\right) $$, then there exists an algorithm $\mathcal {B}$ that breaks the $\zeta$-$\textsf{rGAIP}$ problem with advantage $$\epsilon _\mathcal {B}\ge C_2 \cdot \frac{\epsilon _\mathcal {A} ^2}{\left( {\begin{array}{c}Q\\ \ell + 1\end{array}}\right) ^2 \cdot (\ell + 1)^3}$$ for some universal positive constants $C_1$ and $C_2$. Note we use a *d*-th primitive root of unity $\zeta$ and $\kappa$ denotes the number of parallel repetitions of the underlying sigma protocol.

##### Proof

Upon receiving an ${\textsf{rGAIP}}$ instance, the wrapper proceeds as described in Sect. 4.2. The proof follows from the above Lemmas 23 and 24 and Theorem 3 (i.e., [55, Theorem 1]) and the result follows. $\square$

## Analysis of ring GAIP

This section analyzes the $\zeta _d$-ring group action inverse problem ($\zeta _d$-$\textsf{rGAIP}$) over CSIDH-512. Section 8.1 discusses the existence of primitive $d$-th roots in $\mathbb {Z}_N^\times$, and a method to find one which satisfies Requirement 1. Section 8.2 recalls the most efficient classical and quantum algorithms against $\textsf{GAIP}$ and presents a structural attack on $\zeta _d$-$\textsf{rGAIP}$ which effectively reduces $\zeta _d$-$\textsf{rGAIP}$ for a few choices of *d* to a $\textsf{GAIP}$ instance with a smaller group size compared to the original group considered by $\zeta _d$-$\textsf{rGAIP}$. In Sect. 8.3, we complement our cryptanalysis by proving that $\zeta _d$-$\textsf{rGAIP}$ for a few choices of *d* is as hard as $\textsf{GAIP}$ defined over the same group. This shows optimality of our structural attack for $\zeta _d$-$\textsf{rGAIP}$ for some choices of *d*. We note that the concrete value of *d*’s that admit an attack or a reduction depends on the concrete CSIDH-512 parameter set.

### Finding a root of unity and satisfying requirement 1

We briefly discuss the existence of and a process for finding a primitive $d$-th root of unity $\zeta _d \in \mathbb {Z}_N^\times$ which satisfies Requirement 1. Firstly, it is a straightforward consequence of the fundamental theorem of finitely-generated abelian groups and the definition of $\lambda (N)$ that $\mathbb {Z}_N^\times \cong \mathbb {Z}_{n_1} \times \mathbb {Z}_{n_2} \times \cdots \times \mathbb {Z}_{n_r}$ where $n_1 \,|\, n_2 \,|\, \cdots \,|\, n_r$ and $n_r = \lambda (N)$, so that a *d*-th root of unity exists if and only if *d* is a divisor of $\lambda (N)$—here, $\lambda (\cdot )$ is the Carmichael function.

To find such a root for a given valid *d*, the most intuitive method, perhaps, is to start with a primitive $\lambda (N)$-th root of unity $\zeta _{\lambda (N)}$, and compute $\zeta _{\lambda (N)}^\frac{\lambda (N)}{d}$, which will have order exactly $d$. Unfortunately, this may result in a *d*-th root of unity that does not meet Requirement 1 (even when one exists which satisfies Requirement 1). In particular, we have to ensure that $\zeta$ is a generator modulo all but a small collection of small prime power divisors of *N* to conclude $\eta _d = \textsf{lcm}_{i\in [d-1]}(\textsf{gcd}(\zeta ^i-1,N))=poly(n).$ First, let $N = 2^{f} p_1^{e_1} p_2^{e_2} \cdots p_t^{e_t}$ be the prime decomposition of $N$—note that we treat the prime $2$ separately for later convenience.^12^ By the Chinese remainder theorem we have

$$\begin{aligned} \mathbb {Z}_N^\times \cong \mathbb {Z}_{2^f}^\times \times \mathbb {Z}_{p_1^{e_1}}^\times \times \mathbb {Z}_{p_2^{e_2}}^\times \cdots \times \mathbb {Z}_{p_t^{e_t}}^\times . \end{aligned}$$

Now, for each $j$ such that $d \mid p_j-1$, we may find an element $\gamma _j$ of $\mathbb {Z}_{p_j^{e_j}}^\times$ of order precisely $d$, since $\mathbb {Z}_{p_j^{e_j}}$ is cyclic of order $p_j^{e_j-1}(p_j-1)$.^13^ Let $I_d = \{j \,:\, 1 \le j \le t \text{ and } d \mid p_j\}$. Applying the Chinese remainder theorem again, there exists a solution $\zeta _d \pmod {N}$ to the system

$$\begin{aligned} \zeta _d \equiv {\left\{ \begin{array}{ll} \gamma _j \pmod {p_j} &{} \text{ if } j \in I_d \\ 1 \pmod {p_j^{e_j}} &{} \text{ if } j \in [t]\setminus I_d \\ 1 \pmod {2^f} \end{array}\right. }. \end{aligned}$$

Note that this $\zeta _d$ is coprime to $N$, and moreover for $i = 1,2,\ldots d-1$ we have

$$\begin{aligned} \zeta _d^i&\not \equiv 1 \pmod {p_j} \text{ for } j \in I_d \\ \zeta _d^i&\equiv 1 \pmod {p_j^{e_j}} \text{ for } j \in [t] \setminus I_d \\ \zeta _d^i&\equiv 1 \pmod {2^f} \end{aligned}$$

so that $\eta _d = \textsf{lcm}_{i \in [d-1]}( \textsf{gcd}(\zeta _d^i - 1, N) ) = 2^f \prod _{j \in [t] {\setminus } I_d} p_j^{e_j}$. In particular, if $d$ is chosen so that $2^f\prod _{j \in [t]{\setminus } I_d} p_j = poly(n)$, our $\zeta _d$ satisfies Requirement 1, as required.

Concretely, for the CSIDH-512 parameter sets we have

$$\begin{aligned} N = 3&\times 37 \times 1407181 \times 51593604295295867744293584889 \\&\times 31599414504681995853008278745587832204909,\\ \lambda (N) = 2^3&\times 3^2 \times 5 \times 7^2 \times 47 \times 71 \times 499 \times 43872112495999887537664613\\&\times 111265544030570407933127742061928986637, \end{aligned}$$

and we can construct the following primitive $d$-th roots of unity with respect to CSIDH-512 following this method for $2 \le d \le 9$, 47 and 499:

#### Remark 6

In the list above, we only display *d* that is a prime power. For other composite divisors of $\lambda (N)$, one can obtain the corresponding root by multiplication. For instance, we can obtain $\zeta _{23453}=\zeta _{47}\zeta _{499}$.

For the CSIDH-512 parameter set, the totients of the small prime divisors of $N$ have the following (maximal) small prime power divisors:

$$\begin{aligned} \varphi (3)&: 2 \\ \varphi (37)&: 2^2,\, 3^2 \\ \varphi (1407181)&: 2^2,\, 3,\, 5,\, 47,\, 499\\ \varphi (51593604295295867744293584889)&: 2^3,\, 3,\, 7^2\\ \varphi (31599414504681995853008278745587832204909)&: 2^2,\, 71. \end{aligned}$$

This implies that for the CSIDH-512 parameter we can find only a $4^\text {th}$ root of unity meeting Requirement 1 (with $\eta _4=3$) since only $\mathbb {Z}^\times _3$ has no cyclic subgroups of order 4. For example, for any $3^\text {rd}$ root of unity $\zeta _3$, we always have a 134-bit divisor of $\textsf{gcd}(\zeta _3,N)$. Therefore, $\zeta _4$-$\textsf{rGAIP}$ over CSIDH-512 is the candidate hardness assumption that can be used for our optimized blind signature construction.

In the next subsection, we show that the hardness of $\zeta _d$-$\textsf{rGAIP}$ varies with the choice of $\zeta _d$. Since we believe $\zeta _d$-$\textsf{rGAIP}$ may be of independent interest, we waive Requirement 1 when considering the cryptanalysis.

### Cryptanalysis and structural attack on $\textsf{rGAIP}$

In the previous section, we showed how to choose a root $\zeta _d$ according to the decomposition of the multiplication group of $\mathbb {Z}_N^\times$. In this section, we show that the underlying structure of $\zeta _d$ in each component is related to the security of $\zeta _d$-$\textsf{rGAIP}$ by presenting a concrete cryptanalysis on the overstretched $\zeta _d$-$\textsf{rGAIP}$ with respect to the CSIDH-512 parameters.

*Generic attacks on* $\textsf{GAIP}$

The best known classical algorithm against $\textsf{GAIP}$ is the meet-in-the-middle attack [46, 47] with time complexity $O(\sqrt{|\mathcal {C\ell (O)}|})=O(\root 4 \of {p})$ against $\textsf{GAIP}$.

The best-known quantum algorithm against $\textsf{GAIP}$ is Kuperburg’s algorithm [19, 58, 59, 68, 74]. Typically, given a challenge *E* to find $a \in \mathbb {Z}_N$ such that $E = [\mathfrak {g}^a] *E_0$, we have a hidden shift problem by defining $f(x) = [\mathfrak {g}^x] *E_0$ and $g(x) = [\mathfrak {g}^x] *E$, the permutations *f*, *g* over $\mathcal {E}\hspace{-2.35pt}\ell \hspace{-1.30pt}\ell$ are hidden shifted by *a*. By applying Kuperburg’s algorithm, one can solve $\textsf{GAIP}$ in time complexity $2^{O( \sqrt{\log (|\mathcal {C\ell (O)}|)} )}$. It is not clear whether the additional structure can give an advantage to the adversary by reducing the group size *in general*. The subset $\{ 1,\zeta _d,\ldots ,\zeta _d^{d-1} \}$ forms a group with multiplication instead of addition. Modifying the group action by restricting to the multiplication subgroup of $\mathbb {Z}^\times _N$ does not give a feasible *g* with a hidden shift *a*. Also, $\zeta$ generates the additive group $\mathbb {Z}_N$, so that the quotient group does not help in this case.

#### Structural attack on $\textsf{rGAIP}$

Let $\zeta _d$ be a *d*-th primitive root of unity and *N* be the class number.

We show that the underlying structure of the root in each component of $\mathbb {Z}_N^\times$ is related to security by displaying a structural attack against $\zeta _d$-$\textsf{rGAIP}$ and the efficacy of the attack is related to each $\textsf{gcd}(\zeta _d^i-1,N)$. We remark that the structural attack requires the order *N* to be squarefree. The requirement complies with the Cohen–Lenstra conjecture and our instantiation satisfies the requirement.

The high-level strategy of our structural attack is to break down a $\zeta _d$-$\textsf{rGAIP}$ instance into several $\textsf{GAIP}$ instances over smaller subgroups or quotient groups. The idea is to exploit the differential information of any two curves in the instance and launch a Pohlig-Hellman-type attack. Recall that the instance is of the form $( X_0 = [\mathfrak {g}^{a}] *E_0, X_1 = [\mathfrak {g}^{a \zeta _d}] *E_0, \ldots , X_{d-1} = [\mathfrak {g}^{ a \zeta _d^{d-1}}] *E_0)$. For any two curves $X_i,X_j$ in the instance, there exists a unique group element $[\mathfrak {g}_{ij}] = [\mathfrak {g}^{a\zeta _d^j-a\zeta _d^i}] \in \mathcal {C\ell (O)}$ such that $[\mathfrak {g}_{ij}] *X_i = X_j$. Therefore, recovering the differential element $[\mathfrak {g}_{ij}]$ gives information about *a*. Typically, it is difficult to recover such $[\mathfrak {g}_{ij}]$ due to the size of the group and considering the $\textsf{GAIP}$ of $(X_i,X_j)$. However, depending on the knowledge of $\eta _d$ derived from the public $\zeta _d$, the hardness of the $\textsf{GAIP}$ of the structural $(X_i,X_j)$ can be reduced.

The following two lemmata show the decomposition into two $\textsf{GAIP}$ instances with smaller groups.

##### Lemma 26

Let $(\mathcal {C\ell (O)},\mathcal {E}\hspace{-2.35pt}\ell \hspace{-1.30pt}\ell ,E_0,*)$ be known-order EGA (KOEGA) where $\mathcal {C\ell (O)}$ is cyclic, and the other parameters are as described above. Let $m = \textsf{gcd}(\zeta _d^j - \zeta _d^i, N)$, and let $m' = \frac{N}{m}$. Define $G_{ij}=\langle [\mathfrak {g}^{\zeta ^j_d - \zeta ^i_d}] \rangle$ and note that it is a subgroup of $\mathcal {C\ell (O)}$ with $[\mathfrak {g}_{ij}] \in G_{ij}$. Hence, one can recover $[\mathfrak {g}_{ij}]$ and $a' = a \cdot (\zeta _d^j-\zeta _d^i) \mod m'$ by solving $\textsf{GAIP}$ problem of $(X_i,X_j)$ over the subgroup $G_{ij}$.

##### Proof

$G_{ij}$ is a cyclic subgroup generated by $[\mathfrak {g}^{\zeta _d^j-\zeta _d^i}]$ and $[\mathfrak {g}_{ij}]\in G_{ij}$ by definition. $\square$

Observe that the quotient group $$\mathcal {C\ell (O)}/G_{ij}$$ has order $$m$$, and in fact the map $$\phi :\mathcal {C\ell (O)}/G_{ij} \rightarrow \langle \mathfrak {g}^{m'} \rangle $$ defined by $$\phi (\overline{[\mathfrak {a}]}) = [\mathfrak {a}^{m'}]$$ is an isomorphism. Solving the instance $$ (X_i, X_j)$$ yields $$a \equiv a' \pmod {m}$$. Observe that $$ [\mathfrak {g}^{(a - a')}] *([\mathfrak {g}^{a'}] *E_0) = [\mathfrak {g}^a] *E_0 = X_0$$, and moreover that $$m' \mid (a - a')$$. Thus, to find $$a$$, it suffices to solve the GAIP instance $$ ([\mathfrak {g}^{a'}] *E_0, X_0)$$ over $$\mathbb {Z}_m \cong \langle \mathfrak {g}^{m'} \rangle \cong \mathcal {C\ell (O)}/G_{ij}$$ to find $$a - a'$$. If $$a'$$ has been recovered by solving the GAIP instance $$ (X_i,X_j)$$ over $$G_{ij}$$, we can then easily recover $$a$$. Thus we have established Lemma 27.

##### Lemma 27

Let $(\mathcal {C\ell (O)},\mathcal {E}\hspace{-2.35pt}\ell \hspace{-1.30pt}\ell ,E_0,*)$ be KOEGA where $\mathcal {C\ell (O)}$ is cyclic and the order is squarefree, and let the other parameters be as described above. If $[\mathfrak {g}_{ij}] \in G_{ij}$, then we can recover $[\mathfrak {g}^{a}]$ by solving the GAIP instance $([\mathfrak {g}^{a'}] *E_0, X_0)$ over $\mathcal {C\ell (O)}/G_{ij}$.

We see that the main strength against our structural attack depends on the $\textsf{GAIP}$ hardness with the group size of $\max (|G_{ij}|,|G/G_{ij}|)$. Choosing an appropriate sequence of $(i_\ell ,j_\ell )_{\ell =1}^k$, the root $\zeta _d$ gives the following ascending chain: $\{ 1 \}=G_1< G_2< \cdots < G_k = \mathcal {C\ell (O)},$ where for each $\ell \in [k-1]$, $G_\ell =G_{ij}$ for some distinct $i,j \in [d]$.

We can conclude as follows.

##### Theorem 28

Let $(\mathcal {C\ell (O)},\mathcal {E}\hspace{-2.35pt}\ell \hspace{-1.30pt}\ell ,E_0,*)$ be KOEGA where $\mathcal {C\ell (O)}$ is cyclic and the order is squarefree. Let $\zeta$ be a *d*-th root of unity for *G*. Let

$$\begin{aligned} \{ 1 \}=G_1< G_2< \cdots < G_k = \mathcal {C\ell (O)}\end{aligned}$$

be a chain for *G* where $G_\ell = \{ [\mathfrak {g}^{n(\zeta ^{j_\ell }-\zeta ^{i_\ell })}] \,|\, n \in \mathbb {Z}_N \}$ for some $i_\ell ,j_\ell \in \{ 0,\ldots ,d-1 \}$ for $\ell \in [k-1]$. Given a GAIP adversary $\mathcal {A}$ over the KOEGA model, there exists an $\zeta$-${\textsf{rGAIP}}$ adversary $\mathcal {B}$ running in polynomial time with *O*(*k*) queries to $\mathcal {A}$ such that^14^

$$\begin{aligned} \textsf{Adv}^{\zeta \text{- }{\textsf{rGAIP}}}_{(\mathcal {C\ell (O)},\mathcal {E}\hspace{-2.35pt}\ell \hspace{-1.30pt}\ell ,E_0,*)}(\mathcal {B}) \ge \prod ^{k-1}_{\ell =1} \textsf{Adv}^{\textsf{GAIP}} _{(G_{\ell +1}/G_{\ell },\mathcal {E}\hspace{-2.35pt}\ell \hspace{-1.30pt}\ell , X'_\ell ,*)} (\mathcal {A}). \end{aligned}$$

where $X'_\ell$ is some element of $\mathcal {E}\hspace{-2.35pt}\ell \hspace{-1.30pt}\ell$ chosen in the reduction.

##### Proof

We proceed by induction on $$k$$. When $$k=2$$ it is trivial, since $$G_2/G_1 \cong \mathcal {C\ell (O)}$$, so the algorithm $$\mathcal {B}$$ simply calls $$\mathcal {A} $$ on $$ (X_0, X_1)$$ and returns its output. For larger $$k$$, we apply Lemmas 26 and 27 to find that, to solve a $$\zeta $$-$$\textsf{rGAIP}$$ instance, it suffices to solve GAIP instances in $$G_{k-1}$$ and $$G_k/G_{k-1}$$. By the inductive hypothesis, there exists a polynomial-time adversary $$\mathcal {B}'$$ making $$O(k)$$ calls to $$\mathcal {A} $$ which solves GAIP in $$G_{k-1}$$ with advantage $$\textsf{Adv}^{\zeta \text{- }{\textsf{rGAIP}}}_{(\mathcal {C\ell (O)},\mathcal {E}\hspace{-2.35pt}\ell \hspace{-1.30pt}\ell ,E_0,*)}(\mathcal {B}') \ge \prod ^{k-1}_{\ell =1} \textsf{Adv}^{\textsf{GAIP}} _{(G_{\ell +1}/G_{\ell },\mathcal {E}\hspace{-2.35pt}\ell \hspace{-1.30pt}\ell , X'_\ell ,*)} (\mathcal {A})$$, and $$\mathcal {A} $$ solves the GAIP instance in $$G_k/G_{k-1}$$ with advantage $$\textsf{Adv}^{\textsf{GAIP}} _{(G_{k}/G_{k-1},\mathcal {E}\hspace{-2.35pt}\ell \hspace{-1.30pt}\ell , X'_k,*)} (\mathcal {A})$$ by definition. Our algorithm $$\mathcal {B}$$ is as follows: call $$\mathcal {B}'$$ to solve the GAIP instance in $$G_{k-1}$$, and then call $$\mathcal {A} $$ to solve the GAIP instance in $$G_k/G_{k-1}$$. This algorithm is clearly polynomial-time and makes $$O(k)$$ calls to $$\mathcal {A} $$; moreover, it succeeds with probability

$$\begin{aligned} \textsf{Adv}^{\zeta \text{- }{\textsf{rGAIP}}}_{(\mathcal {C\ell (O)},\mathcal {E}\hspace{-2.35pt}\ell \hspace{-1.30pt}\ell ,E_0,*)}(\mathcal {B})&\ge \textsf{Adv}^{\textsf{GAIP}} _{(G_{k}/G_{k-1},\mathcal {E}\hspace{-2.35pt}\ell \hspace{-1.30pt}\ell , X'_k,*)} (\mathcal {A}) \cdot \prod ^{k-2}_{\ell =1} \textsf{Adv}^{\textsf{GAIP}} _{(G_{\ell +1}/G_{\ell },\mathcal {E}\hspace{-2.35pt}\ell \hspace{-1.30pt}\ell , X'_\ell ,*)} (\mathcal {A}) \\&= \prod ^{k-1}_{\ell =1} \textsf{Adv}^{\textsf{GAIP}} _{(G_{\ell +1}/G_{\ell },\mathcal {E}\hspace{-2.35pt}\ell \hspace{-1.30pt}\ell , X'_\ell ,*)} (\mathcal {A}) \end{aligned}$$

as required. $$\square $$

Using the aforementioned structural attack, the hardness of $\zeta _d$-$\textsf{rGAIP}$ is determined by the size of the largest quotient group $G_{\ell +1}/G_\ell$ for some $\ell \in [k-1]$.

##### Remark 7

We note that $\textsf{gcd}(\zeta _d^i-1,N)$ is divisible by a prime divisor *p* of *N* if and only if $\zeta _d^{\frac{d}{\textsf{gcd}(i,d)}} \equiv 1 \pmod {p}$. Thus we only need to calculate $\textsf{gcd}(\zeta _d^{d'}-1,N)$ for every divisor $d'$ of *d* to find $\eta _d$. In particular, when $d$ is prime, we need only compute $\textsf{gcd}(\zeta _d - 1,N)$ to find $\eta _d$. Therefore, we only need to consider $\textsf{gcd}(\zeta _d-1,N)$ for $d=3,5,7,11,47,499$ for the CSIDH-512 parameter set.

As a consequence, we reduce each $\zeta _d$-$\textsf{rGAIP}$ instance to a $\textsf{GAIP}$ instance with a group size determined by $\zeta _d$. This is summarized in Table 1. For $\zeta _8$, we have a chain $\{ 1 \}=G_{1}< G_{2}< G_{3}< G_{4} < G_{5} = \mathcal {C\ell (O)}$ where $G_2,G_3,G_4$ is of size $\textsf{gcd}(\zeta _8-1,N),\textsf{gcd}(\zeta ^2_8-1,N),\textsf{gcd}(\zeta ^4_8-1,N)$, respectively, and the largest quotient group is $|G_2/G_1| \approx 2^{134}$, which demonstrates the invulnerability of $\zeta _8$-$\textsf{rGAIP}$. For instance, for $\zeta _3$ we have a chain $\{ 1 \}=G_{1}< G_{2} < G_{3} = \mathcal {C\ell (O)}$ where $G_2$ is of size 37 and the largest quotient group is $|G_3/G_2| \approx 2^{251}$. For $\zeta _4$, $\zeta _{47}$ and $\zeta _{499}$ we have a chain $\{ 1 \}=G_{1}< G_{2} < G_{3} = \mathcal {C\ell (O)}$ where $G_2$ is of size 1407181 with the largest quotient group $|G_3/G_2| \approx 2^{236}$. Our cryptanalysis gives an upper bound of $\zeta _d$-${\textsf{rGAIP}}$ from the perspective of $\textsf{GAIP}$. Importantly, $\zeta _4$-$\textsf{rGAIP}$ which we use for our optimized blind signature only seems to lose 2 bits of security compared with $\zeta _2$-$\textsf{rGAIP}$, or equivalently, $\textsf{GAIP}$ over CSIDH-512.

**Table 1:** The upper row denotes $\zeta _d$-$\textsf{rGAIP}$ over CSIDH-512

$\zeta _d$-$\textsf{rGAIP}$	$\zeta _2$	$\zeta _3$	$\zeta _4$	$\zeta _5$	$\zeta _7$	$\zeta _8$	$\zeta _9$	$\zeta _{47}$	$\zeta _{499}$
$\textsf{GAIP}$ with Group Size in $\log _2$	257	251	255	236	161	134	251	236	236

Using our cryptanalysis in Sect. 8.2, we reduce each $\zeta _d$-$\textsf{rGAIP}$ instance into a $\textsf{GAIP}$ instance with a group size summarized in the lower row. Note that $\textsf{GAIP}$ over CSIDH-512 is equivalent to $\zeta _2$-$\textsf{rGAIP}$ over CSIDH-512

### Equivalence between $\textsf{GAIP}$ and $\textsf{rGAIP}$

We complement our cryptanalysis by showing that our attack is optimal for some parameters. Although a few instances of $\zeta _d$-$\textsf{rGAIP}$ were shown to be significantly weaker than the original $\textsf{GAIP}$ over CSIDH-512, we present a surprising condition that allows to reduce $\zeta _d$-$\textsf{rGAIP}$ to the original $\textsf{GAIP}$. This shows that the attack in Table 1 is optimal for those specific choices of $\zeta _d$. We note that though the condition does not cover all cases (including $\zeta _4$ which meets Requirement 1), the result gives us some guidance of the hardness of $\zeta _d$-$\textsf{rGAIP}$.

#### Large $\textsf{gcd}(\zeta _d-1,N) \approx N$

Note first that in this case we do not know how to have an efficient extractor in our optimized sigma protocol due to the large value of $\eta _d$ (see Lemma 19). Requirement 1 is not satisfied.

It is clear that $\textsf{GAIP}$ is never easier than $\zeta _d$-$\textsf{rGAIP}$. The key insight of the reverse reduction is that when $\textsf{gcd}(\zeta _d-1,N) \approx N$ (or $\textsf{gcd}(\zeta _d-1,N) = N/\textsf{poly}$ to be precise), given a $\textsf{GAIP}$ instance we can generate a $\zeta _d$-$\textsf{rGAIP}$ instance by trial and error. Additionally, the success rate can also be amplified by repeatedly invoking the $\zeta _d$-$\textsf{rGAIP}$ oracle and testing the correctness.

Concretely, given $X_0 = [\mathfrak {g}^{a}] *E_0$ and access to an $\zeta _d$-$\textsf{rGAIP}$ adversary $\mathcal {A}$ for a *d*-th root of unity $\zeta _d$, we can construct a $\textsf{GAIP}$ adversary $\mathcal {B}$ which invokes $\mathcal {A}$ on input $(X_0, [a'] *X_0, [a'^{\zeta _d}] *X_0, \ldots , [a'^{\zeta _d^{d-1}}] *X_0)$ where $a'$ is sampled uniformly at random from the subgroup $\{ r^{\zeta _d-1} | r \in \mathcal {C\ell (O)} \}$. Then, $\mathcal {B}$ outputs whatever $\mathcal {A}$ outputs. Since the subgroup is of size $N/\textsf{gcd}(\zeta _d-1,N) = ploy(n)$, the adversary $\mathcal {B}$ invokes $\mathcal {A}$ on a well-formed instance with probability $\textsf{gcd}(\zeta _d - 1, N)/N$, which is non-negligible. We thus obtain the following theorem.

##### Theorem 29

Given any $\zeta _d$-$\textsf{rGAIP}$ adversary $\mathcal {A}$ for a known-order effective group action of the group size *N*, there exists a $\textsf{GAIP}$ adversary $\mathcal {B}$ in time *d* over the same action such that $\textsf{Adv}^{\zeta _d\text {-}{\textsf{rGAIP}}}(\mathcal {A}) \le \frac{N}{\textsf{gcd}(\zeta _d-1,N)} \cdot \textsf{Adv}^{{\textsf{GAIP}}}(\mathcal {B}).$

As a consequence, we know that for CSIDH-512 we have $\zeta _3$, $\zeta _9$, $\zeta _5$, $\zeta _{47}$, $\zeta _{499}$-$\textsf{rGAIP}$ s are as hard as the original $\textsf{GAIP}$ with a reduction loss of factors 37, 37, 1407181, 1407181, 1407181 respectively. Similarly, $\zeta _{117265}= \zeta _5\zeta _{47}\zeta _{499}$ also has a reduction loss of a factor 1407181.

## Performance

We present an overall performance in Table 2 for our protocols instantiated using CSIDH-512. As explained in Sect. 8, we instantiate the $\zeta _d$-${\textsf{rGAIP}}$ assumption with the 4-th root of unity $\zeta _4$ as it is the only parameter that satisfies Requirement 1 while being presumably as hard as $\textsf{GAIP}$ over CSIDH-512. We also analyze the trade-off between our basic blind signature in Sect. 5 and the optimized blind signature using a *d*-th primitive root of unity in Sect. 7. This helps us illustrate the effect of the value *d* on our optimized scheme and may be useful in the future when new group actions where $\zeta _d$-$\textsf{rGAIP}$ is hard are discovered.

The public key is *d* times larger compared to the basic scheme in general, which can be halved when $$d$$ is even and $$\zeta ^\frac{d}{2} = -1$$. Let $$w = \log _2(N)/8$$ denote the byte size of a class group element in $$\mathbb {Z}_N$$ and approximately 2*w* for one elliptic curve in $$\mathcal {E}\hspace{-2.35pt}\ell \hspace{-1.30pt}\ell $$; for example $$w \approx 32$$ for a CSIDH-512 group. In Sect. 5, the sender and user bandwidths and the signature size of the basic blind signature are $$ 4w n$$ B, $$n/8$$ B (i.e., one hash), and $$ 2n(w + n/8)$$ B, respectively. On the other hand, in Sect. 7 the sender and user bandwidths and the signature size of the optimized blind signature are $$2\kappa (wd + w + \log _2 d)$$ B, $$(\kappa \log _2 d)/8$$ B, and $$2\kappa (w+\log _2 d)$$ B, respectively. Now, given the security parameter $$n$$, the number of repetitions $$\kappa $$ with a *d*-th primitive root of unity is required to satisfy $$d^\kappa =2^n$$, i.e., $$n= \kappa \log _2d$$. Therefore, the communication cost of the signer is increased by roughly $$\frac{d\kappa }{2n}$$, while the signature is decreased by roughly $$\frac{n}{\kappa }$$. The computation cost is increased by a factor of $$\frac{d\kappa }{2n}$$ in group action evaluations for both the signer and the user. Concretely, when $$d=4$$, we have $$n = 2 \kappa $$ and thus the signature size is reduced by approximately 50%.

**Table 2:** The overall performance of our blind signature family regarding the bandwidth, the secret key size, the public size, and the signature size using CSIDH-512

	Bandwidth.S	Bandwidth.U	$|\textsf{sk} |$	$|\textsf{pk} |$	$|\sigma |$	Assumption
Basic. (Fig. 3)	16 KB	16 B	16 B	128 B	8 KB	$\textsf{GAIP}$
Fig. 9 with $\zeta _4$	64 KB	16 B	16 B	512 B	4 KB	$\zeta _4$-$\textsf{rGAIP}$
PBS. (Fig. 6)	48 KB	16 B	16 B	128 B	24 KB	$\textsf{GAIP}$

We take $n=128$ and $\textsf{sk}$ is generated by a seed of $n$ bits. The first two rows are our blind signatures and the final row is our (unoptimized) partially blind signature

It takes roughly 40 ms to perform an action on a 2.70 GHz processor [12, 24], and we can estimate the running time in terms of the number of the isogeny action. Since the signing (respectively, verifying) process requires $6 \times 128$ (respectively, $2 \times 128$) actions in Sect. 5, it takes 30 s (respectively, 10 s) for the procedure.

## Data Availability

Data sharing is not applicable to this article as no datasets were generated or analyzed during the current study.

## Footnotes

1 For readability, we focus on blind signatures below when the distinction between the partial and non-partial is insignificant.

2 We note that *proving* the security of a partially blind signature is more subtle and difficult. Indeed, it was only recently that Kastner et al. [55] provided a corrected proof of the Abe-Okamoto (partially) blind signature [4].

3 The notation for the quadratic twist is not totally uniform in the literature. When $E/k :y^2 = x^3 + Ax^2 + x$ and $c \in k^\times {\setminus } k^{\times 2}$ one sometimes denotes $E^c/k :cy^2 = x^3 + Ax^2 + x$. In this work we will always have $-1 \in k^\times {\setminus } k^{\times 2}$ (since $k = \mathbb {F}_p$ and $p \equiv 3 \pmod {4}$), and we will have $E^{-1} \cong E':y^2 = x^3 - Ax^2 +x$ by the change of variables $(x,y) \mapsto (-x,y)$. So this notation—while not usually used in the CSIDH literature—is reasonable, and will be convenient for our protocol description.

4 Note that this is a standard (optimized variant of an) isogeny-based sigma protocol where 0 is removed from the challenge space (see for instance [12]).

5 Since parallel repetition is not required to show blindness, we only focus on the small challenge space for simplicity.

6 For the overview, we will ignore when such $\zeta _d$ exists and how to find them (see Sect. 8.1 for more details).

7 We assume without loss of generality that $\textsf{sk}$ includes $\textsf{pk}$ and $\textsf{state}_\textsf{S}$ includes $(\textsf{pk}, \textsf{sk})$ and omit it when the context is clear. Below, we also assume that $\textsf{state}_\textsf{U}$ includes $\textsf{M}$.

8 Note that the proof in [55] relies on the fact that there are two possible signing keys per public key. Therefore, their proof does not work for the original Schnorr blind signature [29], which is known to be secure if we further rely on the algebraic group model [54].

9 For those unfamiliar with Schnorr-type signatures, we encourage to look at our concrete construction, where the meaning would be clear from context.

10 [55] defined the witness extractors in such a way that it outputs only $(\textsf{sk} _0, \bot )$ or $(\bot , \textsf{sk} _1)$. However, this restriction is not required as long as Lemma 1 (i.e., [55, Corollary 3]) holds. We note that we need this extra relaxation for it to be useful in our *partially* blind signature. Moreover, note that the extractors are only required to output $\textsf{W} '_b$ included in $\textsf{sk} _b = (b, \textsf{W} '_b)$. We use $\textsf{W} '_b$ and $\textsf{sk} _b$ interchangeably for readability.

11 Recall that we assume $\textsf{state}_\textsf{S}$ includes $\textsf{sk}$ (cf. Footnote 7).

12 In CSIDH this is not necessary, since the choice of $p \equiv 3 \pmod {4}$ guarantees that $N = \# \mathcal {C\ell (O)}$ is odd. But this result extends to other groups, and so we treat it in full generality.

13 Since $p_j$ is odd, we have $\lambda (p_j^{e_j}) = \varphi (p_j^{e_j}) = p^{e_j-1}(p_j-1)$ so that $\mathbb {Z}_{p_j^{e_j}}^\times$ is cyclic of order divisible by $d$.

14 Over KOEGA $(\mathcal {C\ell (O)},\mathcal {E}\hspace{-2.35pt}\ell \hspace{-1.30pt}\ell ,E_0,*)$, the group *G* can be represented by $\oplus ^d_{i=1} \mathbb {Z}_{m_i}$. Therefore, it induces the KOEGA $(G_{\ell +1}/G_{\ell },\mathcal {E}\hspace{-2.35pt}\ell \hspace{-1.30pt}\ell , X'_\ell ,*)$ for any $\ell$ in a canonical way by using the operation from $(\mathcal {C\ell (O)},\mathcal {E}\hspace{-2.35pt}\ell \hspace{-1.30pt}\ell ,E_0,*)$.
